# Assessment of Human Immune Responses to H7 Avian Influenza Virus of Pandemic Potential: Results from a Placebo–Controlled, Randomized Double–Blind Phase I Study of Live Attenuated H7N3 Influenza Vaccine

**DOI:** 10.1371/journal.pone.0087962

**Published:** 2014-02-12

**Authors:** Larisa Rudenko, Irina Kiseleva, Anatoly N. Naykhin, Marianna Erofeeva, Marina Stukova, Svetlana Donina, Galina Petukhova, Maria Pisareva, Vera Krivitskaya, Michael Grudinin, Zhanna Buzitskaya, Irina Isakova–Sivak, Svetlana Kuznetsova, Natalie Larionova, Julia Desheva, Irina Dubrovina, Alexandra Nikiforova, John C. Victor, Kathy Neuzil, Jorge Flores, Vadim Tsvetnitsky, Oleg Kiselev

**Affiliations:** 1 Department of Virology, Institute of Experimental Medicine, Saint Petersburg, Russia; 2 Department of Epidemiology and Prophylaxis, Institute of Influenza, Saint Petersburg, Russia; 3 Department of Molecular Virology, Institute of Influenza, Saint Petersburg, Russia; 4 Department of Biotechnology, Institute of Influenza, Saint Petersburg, Russia; 5 Department of Preclinical Trials, Microgen, Moscow, Russia; 6 Program for Appropriate Technologies in Health, Seattle, Washington, United States of America; University of Pittsburgh, United States of America

## Abstract

**Introduction:**

Live attenuated influenza vaccines (LAIVs) are being developed to protect humans against future epidemics and pandemics. This study describes the results of a double–blinded randomized placebo–controlled phase I clinical trial of cold–adapted and temperature sensitive H7N3 live attenuated influenza vaccine candidate in healthy seronegative adults.

**Objective:**

The goal of the study was to evaluate the safety, tolerability, immunogenicity and potential shedding and transmission of H7N3 LAIV against H7 avian influenza virus of pandemic potential.

**Methods and Findings:**

Two doses of H7N3 LAIV or placebo were administered to 40 randomly divided subjects (30 received vaccine and 10 placebo). The presence of influenza A virus RNA in nasal swabs was detected in 60.0% and 51.7% of subjects after the first and second vaccination, respectively. In addition, vaccine virus was not detected among placebo recipients demonstrating the absence of person–to–person transmission. The H7N3 live attenuated influenza vaccine demonstrated a good safety profile and was well tolerated. The two–dose immunization resulted in measurable serum and local antibody production and in generation of antigen–specific CD4^+^ and CD8^+^ memory T cells. Composite analysis of the immune response which included hemagglutinin inhibition assay, microneutralization tests, and measures of IgG and IgA and virus–specific T cells showed that the majority (86.2%) of vaccine recipients developed serum and/or local antibodies responses and generated CD4^+^ and CD8^+^ memory T cells.

**Conclusions:**

The H7N3 LAIV was safe and well tolerated, immunogenic in healthy seronegative adults and elicited production of antibodies broadly reactive against the newly emerged H7N9 avian influenza virus.

**Trial registration:**

ClinicalTrials.gov NCT01511419

## Introduction

Influenza virus strains that commonly infect animals are infrequently transmitted to humans, and when they do, their transmissibility among humans is generally limited, however, when that happens, the chances for reassortment and generation of hybrid strains with human genes of enhanced transmissibility for humans could lead to pandemic situations, particularly when the exposed populations have no antibodies against the emerging strains. Live attenuated influenza vaccines (LAIVs) generated by Institute of Experimental Medicine (IEM) have been used in Russia in persons above 3 year old since 1987. Construction of LAIVs is based on classic reassortment methodology, i.e. six genes from an attenuated donor backbone cold–adapted, attenuated strain are combined with genes coding for hemagglutinin and neuraminidase of circulating influenza virus strains. Currently all licensed LAIVs are produced in embryonated eggs. Since 1997, when highly pathogenic avian influenza viruses began to circulate in Asia, IEM undertook the development of candidate pandemic LAIVs. The first pandemic H5N2 vaccine was registered in Russia in 2008 [1]. Further development related to the development of H5N1, H7N3 and H2N2–based candidate vaccines in consultation with the World Health Organization (WHO) and within a collaborative agreement with Program for Appropriate Technologies in Health (PATH) are in progress and at different stages.

For pandemic surge capacity, egg–based LAIV manufacturing technology has clear advantages over inactivated influenza vaccine (IIV) with its significantly higher yield, needle–free delivery and wider cross–protection. These factors make LAIV an attractive pandemic preparedness option for developing countries, particularly those with very large populations.

Over the last decade influenza viruses of H7 subtype have caused multiple outbreaks in poultry in Europe and Americas and sporadic human infections, prompting the development and evaluation of H7 vaccine candidates. Such pandemic candidate for H7 LAIV was prepared using low-pathogenic avian influenza virus A/mallard/Netherlands/12/00 (H7N3), which is closely related to the H7N7 viruses responsible for highly pathogenic avian influenza outbreaks in the Netherlands and Germany in 2003. The H7N3 LAIV candidate A/17/mallard/Netherlands/00/95 was developed by IEM and in preclinical studies was found to be similar to the master donor virus (MDV) in terms of replication in the respiratory organs of mice and failure to replicate in mouse brain. One dose of a H7N3 LAIV elicited measurable antibody response in mice which was further boosted with a second vaccine dose [2]. The attenuated phenotype of H7N3 LAIV has been confirmed in naïve ferrets, in which the vaccine elicited immune response and protection from subsequent infection with wild–type (*wt*) H7N3 influenza virus challenge.

The current clinical trial was set up to evaluate the safety and immunogenicity of H7N3 LAIV against H7 avian influenza virus of pandemic potential.

## Methods

The protocol for this trial, masking procedure, randomization plan and supporting CONSORT checklist are available as supporting information; see Protocol S1, Procedure S1, Randomization S1 and Checklist S1.

### Viruses employed

(i) Prepandemic vaccine candidate A/17/mallard/Netherlands/00/95 (H7N3) is a live attenuated, cold–adapted (*ca*), temperature–sensitive (*ts*) influenza reassortant virus generated in embryonated chicken eggs by classical reassortment between apathogenic avian influenza *wt* A/mallard/Netherlands/12/2000 (H7N3) virus (CDC, Atlanta, GA) of low pathogenicity to humans and A/Leningrad/134/17/57 (H2N2) *ca/ts* Russian MDV, as previously described [3]. The virus contains six gene segments encoding the internal proteins from the MDV and the HA and NA proteins from the *wt* virus (6∶2 genomic composition). (ii) Avian influenza virus A/Anhui/1/2013 (H7N9) obtained from CDC, Atlanta, GA was used in various assays to assess vaccine cross–reactivity.

Viruses were propagated in 10 to 11 day old embryonated hen's eggs at 32°C.

### Ethics statement

The study was approved by the Ethics Committee under the Ministry of health and social development of Russian Federation (Moscow, Russia, Research Institute of Influenza Ethics Committee (St Petersburg, Russia) and by the Western Institutional Review Board (WIRB) (WIRB PRO number 20111237) and was conducted in compliance with the Declaration of Helsinki. The clinical trial was registered on http://www.clinicaltrials.gov/, registry number NCT01511419.

### Study design, study vaccine

This randomized, double–blinded and placebo–controlled phase 1 study evaluated the safety, tolerability and immunogenicity of H7N3 LAIV in healthy adults. The proposed randomization is a permuted–block randomization (also known as random permuted block or blocked randomization). Subjects were randomly distributed into two groups to receive either vaccine or placebo at a 3∶1 vaccine/placebo ratio. 15 females and 15 mails in the mean age of 30.1 years received vaccine; 4 females and 6 mails in the mean age of 38.5 years received placebo. The randomization scheme was generated according to procedures described in http://www.randomization.com.

Vaccine and placebo were prepared by an unblinded study clinician, with a second unblinded clinician verifying proper labeling of the syringe with the corresponding allocation code. The vaccine/placebo, which had no other identifiers, was hand–delivered to a blinded study clinician who administered it to the participants and recorded the allocation on the case report forms. Vaccine/placebo were administrated intranasally (0.25 mL into each nostril) with a single–use dosing nasal sprayer in an inpatient isolation unit operated by the Research Institute of Influenza.

Both LAIV and placebo were supplied by MICROGEN (Irkutsk, Russia), the manufacturer. The vaccine was formulated to contain 7.0 log_10_ EID_50_ of H7N3 per dose (0.5 mL). The placebo was packaged and labeled to resemble the vaccine, however, in order to maintain the volunteers, the observers and the lab personnel blinded, it was necessary to have unblinded staff prepare and mask the vaccine just before administration. Placebo consisted of normal allantoic fluid from uninfected eggs harvested and purified in the same way as the vaccine. Two doses were given at an interval between doses of 28 days.

### Clinical study

Potential volunteers were screened within 2 weeks prior to the first application of study product. Forty healthy adults (both sexes) aged 18–49 years participated in the study; 30 received vaccine and 10 received placebo. All the participants were admitted to an isolation unit where they received the study product (day 0) and stayed for a total of 7 days. In order to minimize the potential for spread of vaccine virus and the occurrence of natural reassortment with circulating strains the following precautions were taken: 1) the study was conducted outside of the normal influenza season which typically occurs between October and April; 2) subjects were not enrolled if they had an acute illness or respiratory symptoms; 3) subjects were not allowed to leave the isolation unit from the day of vaccination and for the following 6 days; 4) plans were in place to provide antiviral agents (Oseltamivir) in case of prolonged shedding (i.e., beyond 6 days), LAIV H7N3 is susceptible to this antiviral; 5) subjects were tested for influenza virus in nasal swabs prior to vaccination; 6) isolation unit staff were all vaccinated with seasonal vaccine and agreed to take Oseltamivir in case of infection; 7) the study proceeded stepwise, initially enrolling only 12 subjects before the rest of the cohort was exposed to product; daily testing for presence of vaccine virus in nasal swabs was conducted and plans were in place to keep subjects in the isolation unit until shedding was no longer detected; 8) an independent Safety Monitoring Committee review the safety and shedding data for each cohort and recommended continuation of the study before the next cohort was enrolled.

**Figure 1:**
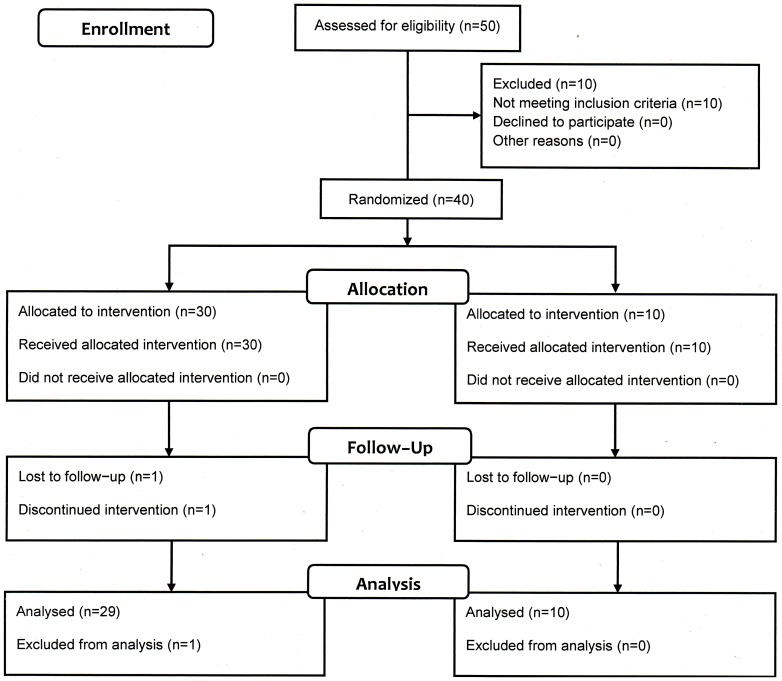
Trial profile. CONSORT 2010 Flow Diagram. The schema graphically outlines the design and conduct of the clinical study. One subject dropped out from the study prior to receiving the second dose of vaccine because of an adverse event not related to the vaccination (adenovirus infection on Day 28 confirmed by PCR).

All subjects were informed about purposes and procedures of the study and the potential risks of participation and signed informed consent forms. Individuals were not enrolled if they had an acute illness of fever at the beginning of the study or in the two previous weeks, or a history of egg allergy. To rule out the presence of other respiratory pathogens, it was necessary in some cases to conduct PCR–based tests. The CONSORT 2010 flow diagram of this study is shown in Figure 1.

### PCR–based virus detection

Nasal secretions for detection of viral shedding by PCR were obtained from each nostril and mixed with transport medium in the same tube. Collections were made on the day of vaccination (days 0 and 28), prior to vaccine administration, 6 hours after each vaccination, and on day 1 through 6 and 29 through 34, after the first and second vaccination, respectively, and tested by PCR for the presence of influenza A virus RNA and to determine virus subtype. RNA extraction from the nasal swabs was performed using “RIBO–sorb” reagent kit for RNA/DNA isolation from human specimens (InterLabService, Central Research Institute of Epidemiology under Rospotrebnadzor, Moscow, Russia). Real–time PCR testing was performed using SuperScript III Platinum One–step qRT–PCR System (Invitrogen) and primers and probes for influenza A virus RNA amplification test with reagents provided by the Centers for disease Control (CDC, Atlanta, USA) to detect and subtype virus RNA. Whenever the presence of other agents was suspected conventional tests were used to detect them.

### Vaccine virus isolation in embryonated chicken eggs

Samples from the nasal secretions obtained on days 1, 2, 3, 5, 7 after the first vaccination and on days 1 (29) and 3 (31) after the second vaccination, were tested for detection of viral shedding by inoculation in 10–11 day old embryonated chicken eggs (“Nazia” poultry plant, St Petersburg, Russia) and incubation at 32°C for 72 hours. The studies were conducted in accordance with the laws of the Russian Federation and complied with the official regulations: “Rules for working with experimental animals” approved on November 13, 1984 by Russian Ministry of Education. Eggs were chilled overnight before harvesting. The presence of influenza virus was detected by standard hemagglutination (HA) test with 1% chicken red blood cells (RBCs) [4]. Allantoic fluids positive for HA were harvested and frozen. If no HA was present after the first passage, the specimens were passaged again, and then for a 3^rd^ time, before finally reporting either inability to recover virus from the specimen or a positive recovery [4]. The isolates were then subtyped as A/H7N3 and their genomic composition was confirmed by partial sequencing.

### Phenotype of isolates

The isolates were characterized as *ca/ts* viruses by culturing in embryonated chicken eggs at the optimal (permissive) (32°C) and non–permissive temperatures (26°C, and 40°C) as previously described [5]. The log_10_ EID_50_/mL calculation was based on the Reed–Muench method [6]. Viruses were considered as *ts* if their titer at elevated temperature of 40°C was ≤4.2 log_10_ EID_50_/mL when titrated in 10–11 day old embryonated chicken eggs (*ts* phenotype). Viruses were considered as *ca* if their titer at low temperature of 26°C was ≥5.7 log_10_ EID_50_/mL (*ca* phenotype).

### Genomic analysis

RNA was isolated from clinical isolates using a QIAamp Viral RNA minikit following the manufacturer's instructions (Qiagen GmbH, Hilden, Germany). The parental origin of the RNA segments of each reassortant virus was determined by sequencing 450–550 nucleotides from each of the 8 genes. To this end viral RNA was subjected to RT-PCR using One–Step RT–PCR Kit (Qiagen GmbH, Hilden, Germany) and a panel of universal primer pairs to enable the amplification of influenza A virus genes of various origin [7]. Sequencing reaction was performed from the same universal primers using a BigDye Terminator v3.1 cycle sequencing kit and 3130×l Genetic Analyzer (Applied Biosystems, Carlsbad, CA, USA) according to manufacturer's instructions. The nucleotide sequences were compared to the consensus sequences of either A/Leningrad/134/17/57 (H2N2) MDV or A/H7N3 virus. To confirm the presence of attenuating mutations specific for the MDV, RNA extracted from clinical isolates was subjected to nucleotide sequencing of the region proximal to the nucleotide of interest. Primers used for RT-PCR and sequencing are listed in Table S1. Multiple sequence alignment analysis was performed using Lasergene version 7.1 sequence analysis software.

### Hemagglutinin inhibition (HAI)

HAI test was performed by standard procedure [4] with human red blood cells utilizing either 2 haemagglutinating units (HAU) or 4 HAU of H7N3. Serum samples were pretreated with RDE (Denka Seiken, Tokyo, Japan). The A/17/mallard/Netherlands/00/95 (H7N3) prepandemic vaccine candidate was used as an antigen to evaluate vaccine–induced responses; the avian influenza virus A/Anhui/1/2013 (H7N9) was used to explore potential cross–reactivity in HAI and microneutralization tests. Four–fold and more in antibody titer rise after vaccination was considered as reliable increase (seroconversion).

### Microneutralization (MN) test

Measurement of serum antibody titers by routine MN test [4] was performed using MDCK cell line. Four–fold or greater antibody titer rises after vaccination were considered as reliable increases.

### Enzyme–linked immunosorbent assay (ELISA)

Influenza virus–specific serum IgG antibodies and local (mucosal) IgA antibodies (Ab) in nasal secretions were tested by ELISA [8] using whole purified the A/17/mallard/Netherlands/00/95 (H7N3) virus at 16 HAU per 0.05 ml for absorption. Nasal secretions were collected using Merocel sponges inserted to each nostril of volunteer for 5 minutes. ELISA titers were expressed as the inversed highest dilution that gave an optical density (OD) equal or greater than twice the mean OD of the control (blank) wells. Four–fold and more antibody titer rises after vaccination were considered as reliable increases.

### Cytokine flow cytometry assay

The percentage of virus–specific CD4^+^IFNγ^+^ and CD8^+^IFNγ^+^ peripheral blood mononuclear cells (PBMC) were determined using flow cytometry cytokine assay as described previously [9], [10]. Overnight *in vitro* stimulation of PBMC was performed using purified A/17/mallard/Netherlands/00/95 (H7N3) virus, RMPI–1640 as a negative control, and Staphylococcal Enterotoxin B (SEB) as a positive control. Activity in the negative control (spontaneous IFNγ production) was subtracted from virus–stimulated PBMC activity for analysis. CD4^+^IFNγ^+^ and CD8^+^IFNγ^+^ T cell populations were analyzed within live cells gate with subsequent determination to central memory cells (Tcm – CCR7^+^CD45RA^−^) and effector memory cells (Tem – CCR7^−^CD45RA^−^). Live/Dead stain (APC), CD4 (AlexaFluor 700), CD8 (ECD), CCR7 (PC7), CD45RA (APC–AlexaFluor 750) and IFN–γ (FITC) were used for staining. Coulter EPICS Altra flow cytometer (Beckman Coulter, Miami, FL, USA) was used for the assay. Increases in cell levels were considered reliable if results exceeded three standard deviations (SD) of the mean values observed in the placebo group.

***Statistical analysis*** of the data was performed by Statistica 6 and GraphPad Prizm 5 software using the Wilcoxon Matched Pairs Test, Friedman ANOVA and Fisher exact test (two–tailed). All the protocol–specified analysis, including the results of the immunogenicity assays were conducted under blind. Unblinding of the study took place only after all the data had been locked.

## Results

### PCR–based virus detection

PCR detection of viral RNA in the nasal swabs obtained within the 7 day–period covering pre– and post–vaccination is shown in Table 1. Influenza A virus RNA was detected during the first 3 days post vaccination in the vaccine recipients. Importantly, the majority of PCR–positive specimens were detected on the 1st day following vaccination. No sizable differences between the first and the second vaccinations regarding frequency of detection of influenza virus RNA were revealed. Overall detection rates were 60% (18/30) and 51.7% (15/29) of subjects after the first and second vaccination, respectively. No viral RNA was detected in any placebo recipients over the 6 days follow–up after either the first or second dose (Table 1).

**Table 1:** Detection of influenza A virus in nasal swabs.

Vaccination	Test article	Virus isolation confirmed						
		By RT–PCR at						By isolation in eggs at Days 1–7^2^
		Before^1^	Day 1	Day 2	Day 3	Days 4–7	Days 1–7^2^	
Vaccination	Vaccine (30)	0/30	18/30	3/30	1/30	0/30	18/30 (60.0%)	4/30 (13.3%)
	Placebo (10)	0/10	0/10	0/10	0/10	0/10	0/10	0/10
Revaccination	Vaccine (29)	0/29	15/29	2/29	1/29	0/29	15/29 (51.7%)	0/29
	Placebo (10)	0/10	0/10	0/10	0/10	0/10	0/10	0/10

1 All subjects were negative before vaccination and revaccination, respectively.

2 Total number of positive subjects.

### Vaccine virus isolation

Of the thirty subjects receiving vaccine 4 (13.3%) shed the vaccine virus. Viral shedding occurred between days 1 and 7 post vaccination following the first vaccine dose: one subject shed virus on day 1, two on day 5, and one on day 7. Three of the four vaccine virus isolates were recovered after the initial passage of nasal swab specimens and one after additional passage in embryonated chicken eggs. The corresponding PCR tests conducted on nasal secretions from which the two day 5 and the one day 7 specimens derived had yielded negative results; no additional material was available to repeat the culture tests.

Replication of vaccine virus was not detected following the second dose in any of the subjects even after three amplification cycles in embryonated chicken eggs (Table 1). None of the 10 placebo recipients shed detectable vaccine virus after either the first or second dose.

### Phenotype of recovered viruses

The four isolates recovered from vaccine recipients retained the phenotypic characteristics (cold adaptation and temperature sensitivity) of the MDV (Table 2). Mean log_10_ reduction of virus titer (EID_50_/mL) at 32°C/40°C was 6.9–7.7. Mean log_10_ reduction of virus titer (EID_50_/mL) at 32°C/26°C was 2.5–3.0.

**Table 2:** Restriction of growth of H7N3 vaccine isolates at different temperatures.

Isolate/virus	Virus titer at 32°C, log_10_ EID_50_/mL	Mean log_10_ reduction of virus titer^1^ (EID_50_/mL) at:		Phenotype
		32°C/40°C	32°C/26°C	
Isolate # 1	8.7	8.7	2.6	*ts*, *ca*
Isolate # 2	9.2	9.2	2.0	*ts*, *ca*
Isolate # 3	8.2	8.2	2.3	*ts*, *ca*
Isolate # 4	9.9	9.9	2.5	*ts*, *ca*
MDV^2^	9.0	9.0	2.5	*ts*, *ca*

1 From the titer at permissive temperature (32°C);

2 A/Leningrad/134/17/57 (H2N2) master donor virus was used as a positive control of *ts* and *ca* markers.

### Genotype of recovered viruses

Nucleotide sequences from the four isolates recovered from vaccine recipients were compared to sequences of the A/Leningrad/134/17/57 (H2N2) and the A/mallard/Netherlands/12/2000 (H7N3) virus. Partial sequencing of all six internal genes showed 100% match with the sequence of corresponding genes of master donor virus A/Leningrad/134/17/57 (H2N2), whereas partial sequences of HA and NA genes 100% matched to A/mallard/Netherlands/12/2000 (H7N3) wild–type strain (Supplement S1).

### Confirmation of the presence of attenuating mutations in recovered viruses

To confirm the presence of attenuating mutations specific for A/Leningrad/134/17/57 (H2N2) MDV, RNA extracted from the four clinical isolates was subjected to partial sequencing of the region of interest. All mutations were confirmed by sequencing of both strands (5′ to 3′ and 3′ to 5′). All four clinical isolates tested were shown to preserve all attenuating mutations described for A/Leningrad/134/17/57 (H2N2) MDV (Table 3, Supplement S2). These data suggest that the vaccine is genetic stable after replication in humans.

**Table 3:** Genetic stability of attenuating mutations of LAIV vaccine reassortant A/17/mallard/Netherlands/00/95 (H7N3) isolates derived from vaccinated subjects.

Gene	N'd	Lwt^1^	L17^2^	H7N3 LAIV^3^	Isolate #				Prot	aa	Lwt	L17	H7N3 LAIV	Isolate #			
					1	2	3	4						1	2	3	4
PB2	1459	G	T	T	T	T	T	T	PB2	478	Val	Leu	Leu	Leu	Leu	Leu	Leu
PB1	819	G	T	T	T	T	T	T	PB1	265	Lys	Asn	Asn	Asn	Asn	Asn	Asn
	1795	G	A	A	A	A	A	A		591	Val	Ile	Ile	Ile	Ile	Ile	Ile
PA	107	T	C	C	C	C	C	C	PA	28	Leu	Pro	Pro	Pro	Pro	Pro	Pro
	1045	G	T	T	T	T	T	T		341	Val	Leu	Leu	Leu	Leu	Leu	Leu
NP	1066	C	C	A	A	A	A	A	NP	341	Leu	Leu	Ile	Ile	Ile	Ile	Ile
M	68	A	G	G	G	G	G	G	M1	15	Ile	Val	Val	Val	Val	Val	Val
	457	T	G	G	G	G	G	G		144	Phe	Leu	Leu	Leu	Leu	Leu	Leu
NS	798	G	A	A	A	A	A	A	NS2	100	Met	Ile	Ile	Ile	Ile	Ile	Ile

1 wild–type strain A/Leningrad/134/57 (H2N2);

2 cold–adapted MDV A/Leningrad/134/17/57 (H2N2);

3 LAIV A/17/mallard/Netherlands/00/95 (H7N3) strain.

### Clinical observations

The vaccine was generally well–tolerated. No serious adverse events occurred. After the first dose, 11 (36.7%) subjects from the vaccinated group and 4 (40.0%) from the placebo group presented an adverse event, mild in most of the cases. After the second dose, 5 (17.2%) subjects from the vaccinated group and 1 (10.0%) from the placebo presented adverse events, most of which were mild. The adverse events observed were limited to sore throat, fever, nasal congestion and catarrhal nasopharynx, sneezing and headache (Table 4). A single subject who presented for the second vaccination with a respiratory infection was diagnosed as having adenovirus in nasal secretions. The subject was not admitted to the isolation unit and thus, did not receive the second vaccination (Figure 1).

**Table 4:** Percentage of adult subjects vaccinated with H7N3 LAIV with solicited local and systemic reactions within 7 days of vaccination.

Reactogenicity event	Treatment group, n (%)			
	LAIV		Placebo	
After dose 1	n = 30	95% CI^3^	n = 10	95% CI
Any solicited local reactions	2 (6.7%)	0.8–22.1	1 (10.0%)	0.3–44.5
Any solicited systemic reactions	11 (36.7%)	19.9–56.1	4 (40.0%)	12.2–73.8
Any solicited local and systemic reactions^1^	11 (36.7%)	19.9–56.1	4 (40.0%)	12.2–73.8
After dose 2	n = 29	95% CI	n = 10	95% CI
Any solicited local reactions	1 (3.4%)	0.1–17.8	0	0
Any solicited systemic reactions	5 (17.2%)	5.8–35.8	1 (10.0%)	0.3–44.5
Any solicited local and systemic reactions^2^	5 (17.2%)	5.8–35.8	1 (10.0%)	0.3–44.5

1 All reactions observed were mild and included sore throat, fever, nasal congestion and catarrhial nasopharinx, sneeze and headache.

2 All reactions observed were mild and included sore throat, fever, cough and nasal congestion.

3 95% confidence interval.

### Antibody responses to H7N3

The assays to evaluate humoral immune responses included hemagglutination Inhibition (HAI) assay, microneutralization (MN), IgG and IgA in serum samples. Mucosal IgA antibody responses were evaluated by ELISA. All subjects had HAI antibody titer of ≤1∶10 against H7N3 vaccine virus prior to the first vaccination. Initially the HAI assay was performed using 4 HAU of the H7N3 vaccine virus. With this assay, 10.3% and 31.0% of the subjects exhibited 4–fold or greater rise in serum antibody titer after one or two doses of vaccine, respectively (Table 5). Because it has been published that sensitivity of the HAI assay may be increased by using 2 HAU [11], [12], we tested the sera using 2 HAU in addition to the pre–planned 4 HAU; 7 of 29 (24.1%) subjects had a 4–fold or greater rise in HAI titer after the first dose, and 13 of 29 (44.8%) subjects had a 4–fold or greater rise in after the second dose in this more sensitive test. Fourfold or greater rises in virus neutralizing antibody titer were observed in serum from 5 (17.2%) subjects following the first dose of vaccine and in 12 (41.4%) following the second dose. The ELISA assays employed A/17/mallard/Netherlands/00/95 (H7N3) virus as the antigen. After the first vaccine dose, fourfold or greater rises in serum IgG titer were observed in 3 (10.3%) subjects, in serum IgA titer in 1 (3.4%) subject, and in mucosal IgA titer in 12 (41.4%) subjects. After the second dose of LAIV the number of these rises increased to 8 (27.6%) for serum IgG, 3 (10.3%) for serum IgA, but did not change for mucosal IgA.

**Table 5:** Antibody responses to A/17/mallard/Netherlands/00/95 (H7N3) LAIV.

Assay	Test article	Number of Ab conversions		Reverse GMTs			GMT fold changes	
		After 1 dose	After 2 doses	Day 0	Day 28	Day 56	II/I	III/I
HAI (4 HAU): serum Ab	LAIV	3 (10.3%)	9 (31.0%)	2.8^1^	3.5	4.7^1^	1.3	1.7
	Placebo	0	0	3.3^2^	3.3^2^	3.5^2^	1.0	1.1
HAI (2 HAU): serum Ab	LAIV	7 (24.1%)	13 (44.8%)^16^	3.0^3^^,^ 4	5.5^3^	7.0^4^	1.8	2.3
	Placebo	0	0^16^	4.1^5^	4.1^5^	4.7^5^	1.0	1.1
MN: serum Ab	LAIV	5 (17.2%)	12 (41.4%)^17^	4.2^6^^,^ 7	6.2^6^^,^ 8	12.4^7^^,^ 8	1.5	3.0
	Placebo	0	0	4.4^9^	4.4^9^	5.0^9^	1.0	1.1
ELISA: serum IgA	LAIV	3 (10.3%)	8 (27.6%)	11.7^10^	13.9^10^	17.6^10^	1.2	1.5
	Placebo	0	0`	18.4^11^	16.0^11^	18.4^11^	0.9	1.0
ELISA: serum IgG	LAIV	1 (3.4%)	3 (10.3%)	12.0^12^	12.9	13.9^12^	1.1	1.2
	Placebo	0	0	13.0^13^	14.9^13^	13.9^13^	1.1	1.1
ELISA: mucosal IgA	LAIV	12 (41.4%)	12 (41.4%)	7.6^14^	14.9^14^	12.6	2.0	1.7
	Placebo	1 (10.0%)	1 (10.0%)	10.6^15^	9.8^15^	9.2^15^	0.9	0.9

1 The HAI antibody GMT after second vaccination was statistically significantly higher than pre–vaccination GMT (Wilcoxon Matched Pairs Test with Bonferroni adjustment: p = 0,0024);

2 There was no statistically significant difference between serum HAI antibody GMTs at three time points in placebo group (Friedman ANOVA: ANOVA Chi Sqr. (N = 10, df = 2) = 0,6667, p = 0,7165);

3 The HAI antibody GMT after first vaccination was statistically significantly higher than pre–vaccination GMT (Wilcoxon Matched Pairs Test with Bonferroni adjustment: p = 0,0012);

4 The HAI antibody GMT after second vaccination was statistically significantly higher than pre–vaccination GMT (Wilcoxon Matched Pairs Test with Bonferroni adjustment: p = 0,0004);

5 There was no statistically significant difference between serum HAI antibody GMTs at three time points in placebo group (Friedman ANOVA: ANOVA Chi Sqr. (N = 10, df = 2) = 2,6667; p = 0,2636);

6 The serum neutralizing antibody GMT after first vaccination was statistically significantly higher than pre–vaccination GMT (Wilcoxon Matched Pairs Test with Bonferroni adjustment: p = 0,0166);

7 The serum neutralizing antibody GMT after second vaccination was statistically significantly higher than pre–vaccination GMT (Wilcoxon Matched Pairs Test with Bonferroni adjustment: p = 0,0001);

8 The serum neutralizing antibody GMT after second vaccination was statistically significantly higher than GMT after first vaccination (Wilcoxon Matched Pairs Test with Bonferroni adjustment: p = 0,0025);

9 There was no statistically significant difference between serum neutralizing antibody GMTs at three time points in placebo group (Friedman ANOVA: ANOVA Chi Sqr. (N = 10, df = 2) = 2,000, p = 0,3679);

10 There was no statistically significant difference between serum IgG GMTs at three time points in vaccinated group (Friedman ANOVA: ANOVA Chi Sqr. N = 29, df = 2) = 0,2222, p = 0,8948);

11 There was no statistically significant difference between serum IgG GMTs at three time points in placebo group (Friedman ANOVA: ANOVA Chi Sqr. (N = 10, df = 2) = 3,000, p = 0,2231);

12 The serum IgA GMT after second vaccination was statistically significantly higher than pre–vaccination GMT (Wilcoxon Matched Pairs Test with Bonferroni adjustment: p = 0,0157);

13 There was no statistically significant difference between serum IgA GMTs at three time points in placebo group (Friedman ANOVA: ANOVA Chi Sqr. (N = 10, df = 2) = 2,6667, p = 0,2636);

14 The mucosal IgA GMT after first vaccination was statistically significantly higher than pre–vaccination GMT (Wilcoxon Matched Pairs Test with Bonferroni adjustment: p = 0,0031);

15 There was no statistically significant difference between mucosal IgA GMTs at three time points in placebo group (Friedman ANOVA: ANOVA Chi Sqr. (N = 10, df = 2) = 0,3077, p = 0,8574);

16 Percent with ≥fourfold HAI antibody titer rise after two doses of LAIV A(H7N3) was statistically significantly higher than in placebo group (Fisher exact (two–tailed) p = 0,0161).

17 Percent with ≥four fold serum neutralizing antibody titer rise after two doses of LAIV A(H7N3) was statistically significantly higher than in placebo group (Fisher exact (two–tailed) p = 0,0172).

Overall, the largest proportion of seroconversions was observed with the HAI assay, the MN and the mucosal IgA ELISA (44.8%, 41.4% and 41.4% respectively).

When measured by various assays, antibody geometric mean titers (GMT) increased by 1.3–2.0 fold after first and in 1.2–3.0 fold after second vaccination with the exception of mucosal IgA which did not increase after the second vaccination. Highest GMT rises were observed in the HAI assay when using 2 HAU of antigen in the test (2.3 fold), microneutralization (3.0 fold) and mucosal IgA ELISA (1.7 fold). Antibody GMT in the placebo group did not change significantly compared to Day 0 (0.9–1.3 fold for the various assays).

### T cell responses

H7N3–specific CD4^+^ and CD8^+^ T cell responses were examined in PBMCs obtained from all the study subjects before vaccination (Day 0), 28 days after the first vaccination (Day 28) and 28 days after revaccination (Day 56). To calculate the frequency of virus–specific CD4^+^ and CD8^+^ T cells we quantified all cells positive for IFNγ after *in vitro* stimulation with whole H7N3 virion. To address the somewhat broad variability observed in baseline levels we present the data as fold changes (FC) representing a ratio between the percentage of specific T–cell levels after vaccination in comparison with the pre–vaccination levels. Increases in the antigen–specific CD4+T–cell levels exceeding 3 standard deviations from the levels in the mean of the placebo–were considered positive responses. Such responses were detected in 2 of 29 (6.9%) subjects after the first dose, and 3 (10.3%) subjects after the second dose (Table 6). CD8^+^ T cell responses assessed in a similar way were observed in 5 (17.2%) of the subject after the second dose of A (H7N3) LAIV only.

**Table 6:** T cell induction after *in vitro* stimulation of PBMC from subjects vaccinated with LAIV A/17/Mallard/Netherlands/00/95 (H7N3).

Cell Populations	Group	Number (%) of persons with significant increases^1^		
		After dose 1	After dose 2	After 2 doses (1+2)
CD4^+^ IFNγ^+^	LAIV^2^	2 (6.9%)	3 (10.3%)	4 (13.8%)
	Placebo^2^	0	0	0
CD4^+^ IFNγ^+^ T cm	LAIV	5 (17.2%)	6 (20.7%)	7 (24.1%)
	Placebo	0	0	0
CD4^+^ IFNγ^+^ T em	LAIV	1 (3.5%)	3 (10.3%)	3 (10.3%)
	Placebo	0	0	0
CD8^+^ IFNγ^+^	LAIV	0	5 (17.2%)	5 (17.2%)
	Placebo	0	0	0
CD8^+^ IFNγ^+^ T cm	LAIV	1 (3.5%)	6 (20.7%)	6 (20.7%)
	Placebo	0	0	0
CD8^+^ IFNγ^+^ T em	LAIV	0	5 (17.2%)	5 (17.2%)
	Placebo	0	0	0

1 Data exceeding 3 SD of placebo mean value.

2 LAIV (n = 29), placebo (n = 9). When calculating placebo mean value the data from one of the subjects were not available because of the sample damage.

After two doses of vaccine H7N3 antigen–specific CD4^+^ central memory, T cells significantly increased in 6 (20.7%) of the subjects and effector memory T cells in 3 (10.3%) of the subjects; 6 of the subjects (20.7%) had significant increases in CD8^+^ central memory T cells, but not in CD8^+^ effector memory T cells. Overall, the percentage of subjects with significant CD4^+^ and CD8^+^ T cell increases, respectively, was 10 and 24% after dose 1 and dose 2/for CD4^+^IFNγ^+^, and 17 and 21% after dose 1 and dose 2 for CD8^+^IFNγ^+^ cells (Table 6). Thus, up to 24% of subjects responded with significant increases in virus–specific CD4^+^ and CD8^+^ T cells after two–dose vaccination with A/17/mallard/Netherlands/00/95 (H7N3) LAIV.

Table 7 contains cumulative data on all the immune responses observed in subjects in the clinical study. Occurrences of antibody conversions and cellular responses after first and/or second doses of A (H7N3) LAIV are designated by “+”. Altogether, 82.8% of persons responded to vaccination with antibody conversions by one or more tests. Significant increases in one or more T cell subpopulations were obtained in 41.4% of vaccinated subjects. Summarily, detected antibody and/or cellular immune responses occurred in 86.2% of vaccinated persons.

**Table 7:** Cumulative data on immune responses to A/17/Mallard/Netherlands/00/95 (H7N3) LAIV in vaccinated subjects after the first and/or the second doses.

Subject #	Antibody immune response (conversions)							Cellular immune response (≥3 SD of placebo mean value)							Any immune response
	HAI (4 HAU)	HAI (2 HAU)	MN	Serum IgA ELISA	Serum IgG ELISA	Mucosal IgA ELISA	Any antibody response	CD4^+^	CD4^+^ Tcm	CD4^+^ Tem	CD8^+^	CD8^+^ Tcm	CD8^+^ Tem	Total	
1		**+** 1					**+**	**+**	**+**			**+**		**+**	**+**
2															
3	**+**	**+**				**+**	**+**								**+**
4			**+**				**+**								**+**
5			**+**				**+**	**+**	**+**	**+**	**+**	**+**	**+**	**+**	**+**
6	**+**	**+**				**+**	**+**				**+**	**+**	**+**	**+**	**+**
7	**+**	**+**	**+**			**+**	**+**								**+**
8		**+**				**+**	**+**			**+**				**+**	**+**
9	**+**	**+**	**+**	**+**	**+**		**+**	**+**	**+**					**+**	**+**
10															
11				**+**		**+**	**+**								**+**
12															
13	**+**	**+**	**+**				**+**								**+**
14				**+**	**+**	**+**	**+**	**+**	**+**		**+**		**+**	**+**	**+**
15			**+**	**+**	**+**	**+**	**+**		**+**		**+**	**+**	**+**	**+**	**+**
16			**+**	**+**			**+**								**+**
17									**+**					**+**	**+**
18	**+**	**+**		**+**			**+**								**+**
19		**+**		**+**		**+**	**+**					**+**		**+**	**+**
20			**+**			**+**	**+**				**+**	**+**		**+**	**+**
21			**+**				**+**								**+**
22		**+**				**+**	**+**								**+**
23			**+**				**+**		**+**	**+**				**+**	**+**
24	**+**	**+**					**+**								**+**
25															
26	**+**	**+**		**+**		**+**	**+**								**+**
27			**+**				**+**								**+**
28			**+**				**+**						**+**	**+**	**+**
29	**+**	**+**				**+**	**+**								**+**
No	9	13	12	8	3	12	24	4	7	3	5	6	5	12	25
%	31.0	27.6	41.4	27.6	10.3	41.4	82.8	13.8	24.1	10.3	17.2	20.7	17.2	41.4	86.2

1 Occurrences of antibody conversions or cellular responses after the first and/or the second doses of A (H7N3) LAIV.

### Cross reactive antibody responses

There are reasons to believe that H7N3 LAIV may elicit production of broadly reactive antibodies which could neutralize the newly emerged A/Anhui/1/2013 (H7N9) avian influenza virus. When the serum samples obtained after the second H7N3 vaccinations were tested against H7N9 virus, seroconversions were observed by HAI and MN, in 34,5% and 17.2% of the vaccinated participants, respectively, with 44.8% of the subjects presenting a seroconversion by either assay in post second vaccination samples (Table 8).

**Table 8:** Cross–reactivity provided by H7N3 LAIV to H7N9 avian influenza virus.

Antigen	Assay	Test article	Dose	Number of subjects	Seroconversions	
					Number	%
N7N3^1^	HAI	H7N3 LAIV	1	30	8	26.7
			2	29	13	44.8
		Placebo	1	10	0	0
			2	10	0	0
	MN	H7N3 LAIV	1	30	5	16.7
			2	29	12	41.4
		Placebo	1	10	0	0
			2	10	0	0
	HAI+MN	H7N3 LAIV	1	30	13	43.3
			2	29	22	75.9
		Placebo	1	10	0	0
			2	10	0	0
N7N9^2^	HAI	H7N3 LAIV	1	30	6	20.0
			2	29	10	34.5
		Placebo	1	10	0	0
			2	10	0	0
	MN	H7N3 LAIV	1	30	2	6.7
			2	29	5	17.2
		Placebo	1	10	0	0
			2	10	0	0
	HAI+MN	H7N3 LAIV	1	30	8	26.7
			2	29	13	44.8
		Placebo	1	10	0	0
			2	10	0	0

1 A/17/mallard/Netherlands/00/95 (H7N3) LAIV;

2 A/Anhui/1/2013 (H7N9).

## Discussion

A number of H7 influenza vaccines have been developed and clinically tested over the years. The majority of these vaccines are inactivated whole virion or split vaccines formulated with or without adjuvant. Live influenza vaccine H7N3 derived from A/chicken/British Columbia/CN–6/2004 (H7N3) avian influenza virus and A/Ann Arbor/1/60ca as MDV have been produced and tested in phase I clinical trial on 21 healthy adults by the US manufacturer MedImmune. In that study, two doses of LAIV of wild–type low pathogenic avian influenza virus A/chicken/British Columbia/CN–6/2004 (H7N3) were tested and found to be generally well tolerated and immunogenic. Replication of the vaccine was restricted, especially after second dose. Despite that the majority of the subjects (90%) developed an immune response as measured by any assay [12]. The best immune response was detected by ELISA for HA–specific serum IgA (71%).

In our clinical trial the A/17/mallard/Netherlands/00/95 (H7N3) LAIV based on A/Leningrad/134/17/57 (H2N2) MDV elicited only mild adverse events. According to the Russian requirements on LAIV, moderate adverse events including body temperature rise in between 37,6–38,4°C after vaccination with LAIV must not exceed 2%. The number (percentage) of mild adverse events is not regulated. MedImmune paper [12] also reported that no serious adverse events occurred after vaccination with live attenuated H7N3 vaccine. In particular, after the first vaccination 19% of participants displayed mild adverse events like headache, myalgia (10%) and other mild adverse events (76%).

A/17/mallard/Netherlands/00/95 (H7N3) LAIV demonstrated a similar level of vaccine virus replication in humans when compared to data from H7N3 Ann Arbor LAIV study [12]. When tested by PCR in nasal secretions, the A/17/mallard/Netherlands/00/95 vaccine virus was shown to be able to replicate for up to three days post immunization in 18/30 (60.0%) of our subjects after the initial vaccination. This rate is comparable to that observed in the earlier MedImmune study with H7N3 Ann Arbor LAIV in which 17/21 (81%) of vaccinated subjects shed the virus after the first dose. However, upon re–vaccination, 15/29 (51.7%) of the subjects in our study shed virus compared to 0/17 (0%) in the MedImmune study. The reason for this difference might be related to either the different HA or the different backbones between the strains by the variations in HA sequence differences between the two H7N3 viruses: A/chicken/British Columbia/CN–6/2004 (H7N3) belongs to North American lineage of H7 viruses, whereas A/mallard/Netherland/12/00 belongs to a divergent Eurasian lineage [13], [14]; avian–human transmission of H7 viruses occurred mostly among viruses from Eurasian lineage [15].

Phenotypic (cold adaptation and temperature sensitivity) and genotypic (sequence analyses) conducted on the viruses recovered from four of the volunteers suggests that the vaccine is genetically stable after *in vivo* passage. However, given the small number of observations and the discordance between the PCR results and culture isolation for three of the four isolates, further studies will need to be conducted to confirm this observation.

Of note, vaccine virus was not detected in placebo group supporting a notion for the lack of person–to–person transmission of the vaccine virus. The vaccine also demonstrated good safety profile and was well tolerated.

One of the most crucial factors for licensing new influenza vaccines is the level of vaccine immunogenicity, as detected by standard assays accepted by regulatory agents in various places. For inactivated vaccines HAI titers are an accepted correlate of protection. However, HAI titers do not appear to correlate with protection against influenza by LAIV [16] yet for avian LAIV directed against pandemic strains where clinical efficacy cannot be measured in pre–pandemic period, a multiplicity of assay may reveal a better correlate. To further characterize the H7N3 vaccine, we tested the sera from all the volunteers using several additional assays, including microneutralization and detection of serum IgG and IgA antibodies as well as mucosal IgA antibody, by ELISA. Cumulative data on antibody immune responses showed that 25/29 (86.2%) of evaluable vaccinated subjects had serum and/or mucosal antibodies generated as a result of vaccination. However, to the rate of serum antibody conversions and the GMT values observed with A (H7N3) vaccine in this study were lower than typically seen for seasonal LAIV, which partially can be explained by the preferential binding of avian H7 virus with avian type receptors but not to those present in humans.

Regulatory requirements for live influenza vaccine, in effect since 1978 considered induction of serum antibodies revealed by the HAI assay as the only criterion for LAIV immunogenicity [17]. This approach was based on anti–influenza immunity data from the late 1960s–early 1970s when antibodies circulating in the blood were the only known factor that correlated with protection. Since then, our knowledge about anti–influenza immunity has greatly increased. It has been demonstrated that LAIV and inactivated influenza vaccine (IIV) do not induce the same type of immune response: LAIV induces humoral and cellular immune protection locally, at the initial site of infection, the respiratory tree, while IIV primary induces antibodies circulating in the blood [18]. The generation of local B and T cellular immune memory appears to be the principal anti–influenza protection mechanism with LAIV [10] while the serum immune responses are recognized as a good correlate of vaccine protection for IIV. WHO considers important to assess the potential efficacy of LAIV by measuring not only humoral response but also innate, mucosal and cellular immune responses [19]. LAIV is also known to induce local innate immune responses such as interferon and other cytokines as well as adaptive immune responses including secretory mucosal antibodies and, as noted above specific T–cell responses which mimic the T cell responses identified during naturally occurring influenza infection in humans.

LAIV strains developed at the IEM and based on seasonal [20], pandemic [21] or viruses with pandemic potential [22] were shown to be able to stimulate CD4^+^ and CD8^+^ T cells. The same observation is presented here for H7N3 LAIV. The percentage of subjects with increases in T cells levels after 2 doses was 14% when assessing interferon gamma–producing CD4^+^ cells and 17% for interferon gamma–producing CD8^+^ cells. A most important feature of the immunogenicity of LAIV is its ability to induce immunological memory which provides accelerated and rapid immune response to the second antigen encounter, especially in the case of drifted and shifted influenza viruses. For analysis of induction central and effector memory cells in this study we used anti CCR7 and anti CD45RA antibodies. Up to 24.1% of vaccinated subjects had significant response in virus–specific memory T cells.

Our study also shows the cross–reactive potential of the H7N3 LAIV strain against an H7N9 virus. Sera from some of the H7N3–vaccinated subjects elicited heterosubtypic antibodies able to neutralize newly emerged H7N9 virus which caused considerable public health impact in China in the spring of 2013. Furthermore, analysis of HA sequences using the latest bioinformatics tools, allowed us to identify conserved immune epitopes for cytotoxic T cells and B cells between H7N3 and H7N9 viruses [23]. Importantly, the A/mallard/Netherlands/12/2000 virus belongs to the Eurasian lineage of H7 viruses, same as the newly emerged H7N9 strains [24], [25], which means that the majority of B cells and cytotoxic T cells induced by H7N3 virus are likely able to recognize the new H7N9 virus. These data are especially important in the event that an H7N9 pandemic begins. The H7N3 LAIV strain could be readily used for prime immunization of the population in the face of the pandemic, until specific H7N9 vaccines are released for wider use. In addition, LAIV strain can be produced in large quantities during short period of time, which will be critical if the pandemic is announced.

## Conclusions

The administration of two doses of H7N3 LAIV to healthy adults demonstrated that the vaccine was generally safe and well–tolerated. No vaccine–related serious adverse events occurred in this phase 1 clinical trial. Vaccine virus was detected by PCR in nasal secretions for up to three days in 60.0% of the subjects after the first dose and 51.7% after the second dose. Whereas the vaccine and placebo recipients were allowed for some interaction within the isolation unit, vaccine virus was not detected in the placebo group, demonstrating the absence of person–to–person transmission.

Two–dose immunization of healthy adults with H7N3 LAIV resulted in serum and mucosal antibody responses and generation of CD4^+^ and CD8^+^ immunological memory T cells. Up to 24.1% of subjects from vaccinated group respond with serum antibody conversions after the first dose of LAIV and up to 44.8% after the second dose. Mucosal antibody rises were observed in 41.4% of persons after the first vaccination and up to 41.4% of subjects responded with increases in H7N3–specific CD4^+^ and/or CD8^+^ T cells after two–dose vaccination.

We also demonstrated that H7N3 LAIV elicited production of broadly reactive antibodies which recognized not only the wild–type avian H7N3 but also the newly emerged H7N9 influenza viruses. Subjects receiving H7N3 LAIV responded to the divergent H7N9 virus. 43.3% and 75.9% of subjects had enhanced HAI+MN titers against H7N3 after the first and the second vaccination, correspondingly. Interestingly, 26.7% and 44.8% of subjects had enhanced HAI+MN titers against H7N9 after the first and the second vaccination, correspondingly.

We believe that all the data presented is promising and may be warrant a future phase II clinical trial.

## Supporting Information

Checklist S1 **CONSORT 2010 checklist of information to include when reporting a randomized trial.**
(PDF)

Procedure S1 **Masking procedures for Protocol LAIV-H7N3-01.**
(PDF)

Protocol S1 **Protocol LAIV-H7N3-01 for clinical trial “Reactogenicity, safety, and immunogenicity of a live monovalent A/17/mallard/Netherlands/00/95 (H7N3) influenza vaccine”.**
(PDF)

Randomization S1 **Randomization Plan for PVS Protocol LAIV-H7N3-01.**
(PDF)

Supplement S1 **Representative results of partial sequencing of four H7N3 LAIV clinical isolates from universal primers designed by Hoffmann et al** [**7**] **.**
(PDF)

Supplement S2 **Results of detection of attenuating mutations in internal genes of four H7N3 LAIV clinical isolates using partial sequencing.**
(PDF)

Table S1 **List of primers used for RT–PCR and sequencing analysis of H7N3 LAIV clinical isolates.**
(PDF)
