# Supplementary material for: Assessment of Human Immune Responses to H7 Avian Influenza Virus of Pandemic Potential: Results from a Placebo–Controlled, Randomized Double–Blind Phase I Study of Live Attenuated H7N3 Influenza Vaccine
Source: PLoS One. 2014 Feb 12;9(2):e87962. doi: 10.1371/journal.pone.0087962 (PMC3922724; doi:10.1371/journal.pone.0087962)
Supplement: Checklist S1 — CONSORT 2010 checklist of information to include when reporting a randomized trial. (PDF) [file pone.0087962.s001.pdf]

CONSORT 2010 checklist of information to include when reporting a randomised trial*

Section/Topic	Item No	Checklist item	Reported in
**Title and abstract**			
	1a	**Identification as a randomised trial in the title**<br>Assessment of Human Immune Responses to H7 Avian Influenza Virus of Pandemic Potential: Results from Double–Blind Randomized Phase I Study of Live Attenuated H7N3 Influenza Vaccine	Manuscript, Title
	1b	**Structured summary of trial design, methods, results, and conclusions**<br>**INTRODUCTION.** Live attenuated influenza vaccines (LAIVs) are being developed to protect humans against future epidemics and pandemics. This study describes the results of a double–blinded randomized Phase I clinical trial of cold–adapted and temperature sensitive H7N3 live attenuated influenza vaccine candidate in healthy seronegative adults.<br>**OBJECTIVE.** The goal of the study was to evaluate the safety, tolerability, immunogenicity and potential shedding of H7N3 LAIV.<br>**METHODS, FINDINGS.** Two doses of H7N3 LAIV or placebo were administered to 40 randomly assigned subjects (30 received vaccine and 10 placebo) at Day 1 and Day 28. The presence of influenza A virus RNA in nasal swabs was detected in 60.0% and 51.7% of subjects after the first and second vaccination, respectively. Live vaccine virus was recovered in embryonated eggs from nasal samples obtained from 4 out of 30 vaccine recipients. The recovered virus in these subjects retained its cold–adapted and temperature sensitive phenotypic characteristics and did not revert at nucleotides known to confer the attenuating phenotype. In addition, vaccine virus was not detected in nasal samples obtained from placebo recipients demonstrating the absence of person–to–person transmission. The H7N3 live attenuated influenza vaccine demonstrated good safety profile and was well tolerated. The two–dose immunization resulted in measurable raises in serum and local antibody production and in generation of influenza–specific $CD4^+$ and $CD8^+$ memory T cells. Composite analysis of the immune response which included hemagglutinin inhibition assay, microneutralization tests, and measures of IgG and IgA and virus–specific T cells showed that the majority (86.2%) of vaccine recipients developed serum and/or local antibodies responses and generated $CD4^+$ and $CD8^+$ memory T cells.<br>**CONCLUSIONS.** The H7N3 LAIV was safe and well tolerated, immunogenic in healthy seronegative adults and elicited production of broadly reactive antibodies which recognized not only the wild–type avian H7N3 but also the newly emerged H7N9 influenza viruses.	Manuscript, Abstract section
**Introduction**			
Background and objectives	2a	**Scientific background and explanation of rationale**<br>For pandemic surge capacity, egg–based live attenuated influenza vaccine (LAIV) manufacturing technology has clear advantages over inactivated influenza vaccine with its significantly higher yield, needle–free delivery and wider cross–protection. These factors make LAIV an attractive pandemic preparedness option for	Manuscript, Introduction section

		developing countries, particularly those with very large populations. A number of H5, H7, H9 influenza vaccines have been developed and clinically tested over the years. The majority of these vaccines are inactivated. The study described in this paper was set up to evaluate the safety and immunogenicity of Russian H7N3 LAIV against H7 avian influenza virus of pandemic potential.	
	2b	**Specific objectives or hypotheses**<br>In the current study we evaluated post–vaccination viral shedding, genotype and phenotype of shed virus, and the safety and immunogenicity of H7N3 LAIV which was developed against H7 avian influenza virus of pandemic potential.	
**Methods**			
Trial design	3a	**Description of trial design (such as parallel, factorial) including allocation ratio**<br>This was double–blind, placebo–controlled, randomized (3:1) Phase I Study of H7N3 LAIV conducted in Russia.	Protocol S1, Page 25
	3b	**Important changes to methods after trial commencement (such as eligibility criteria), with reasons**<br>No changes were made to the methods after trial commencement.	
Participants	4a	**Eligibility criteria for participants**<br>The following criteria must be met before a subject may be enrolled for participation:<br>• Legal male or female adult 18 through 49 years of age at the enrolment visit.<br>• Literate and willing to provide written informed consent.<br>• Free of obvious health problems, as established by the medical history and screening evaluations, including physical examination.<br>• Capable and willing to complete diary cards and willing to return for all follow−up visits<br>• Willing to comply with the rules of the isolation unit (including willing and able to take Oseltamivir influenza antiviral medication, should that be recommended by a study physician).<br>• For females, willing to take reliable birth control measures throughout the entire period of participation in the study.	Protocol S1, Page 27
	4b	**Settings and locations where the data were collected**<br>The study took place at the inpatient isolation unit operated by the Research Institute of Influenza and was conducted outside of the normal influenza season. The subjects were separated in two cohorts. First cohort (12 subjects (9 vaccine and 3 placebo) received the first dose of vaccine and independent Safety Monitoring Committee subsequently reviewed blinded safety and virus shedding data to assess vaccine safety and allowed the second cohort (28 subjects, 21 vaccine and 7 placebo) to be enrolled. The procedure was repeated for the second dose administration.	Protocol S1, Page 1, 45
Interventions	5	**The interventions for each group with sufficient details to allow replication, including how and when they were actually administered**<br>Test articles (vaccine and placebo) were administrated intranasally (0.25 mL into each nostril, 0.5 ml total) with a single–use dosing nasal sprayer. Two doses were administered 28 days apart. Subjects were housed in isolator unit for a week after each dose and allowed to leave only if their nasal samples were PCR–negative for influenza	Protocol S1, Page 34

		virus for at least two days prior to the day of release.	
Outcomes	6a	**Completely defined pre–specified primary and secondary outcome measures, including how and when they were assessed**<br>**PRIMARY OUTCOME MEASURES**<br>The safety profile was parameterized as the proportion of subjects experiencing adverse events of the following four categories:<br>• Immediate reactions occurring within two hours of administration of any dose, measured as observed by study staff or reported by the subject to study staff.<br>• Adverse events commonly associated with intranasal vaccination (solicited local and systemic reactions) occurring greater than two hours after administration of any dose of study vaccine or placebo through 7 days following any dose, measured as observed by study staff or reported by the subject to study staff.<br>• All other adverse events (including unsolicited events) occurring during the 7 days following any dose, measured as observed by study staff or reported by the subject to study staff. This includes abnormal laboratory findings from blood and urine specimens collected on Days 7 and 35.<br>• All serious adverse events occurring within 4 weeks of receipt of any dose, as observed by study staff, reported by the subject to study staff, or noted by the subject on a diary card. This includes abnormal laboratory findings from blood and urine specimens collected on Days 28 (pre−vaccination) and 56.<br>**SECONDARY OUTCOME MEASURES**<br>Immune responses was parameterized as the proportion of subjects with at least a four−fold rise after each dose from baseline or as the mean titre after each dose in any of the following:<br>• Serum hemagglutination inhibition (HAI) antibodies.<br>• Serum neutralizing antibodies.<br>• Serum immunoglobulin class A (IgA) or immunoglobulin class G (IgG) antibodies measured by enzyme−linked immunoassay (EIA).<br>• Mucosal IgA antibodies in nasal wick specimen.<br>Virus shedding was parameterized as the proportion of subjects shedding virus (detected by RT−PCR in nasal or conjunctival swabs or by isolation in chicken embryos) at any time−point. Shedding data was reported for every subject at every measured time−point. Molecular characterization of any isolated shed virus was reported with sequence details of any loss of attenuation mutations.	Protocol S1, Page 23
	6b	**Any changes to trial outcomes after the trial commenced, with reasons**<br>No changes were made.	
Sample size	7a	**How sample size was determined**<br>There were no pre−set statistical hypotheses to be tested in this trial. All objectives were descriptive. As such, no sample size was calculated. Total numbers to be enrolled in the trial were consistent with those of phase 1 vaccine trials.	Protocol S1, Page ix
	7b	**When applicable, explanation of any interim analyses and stopping guidelines**<br>Not applicable.	

Section/Topic	Item No	Checklist item	Reported on page No
Randomisation:			
Sequence generation	8a	**Method used to generate the random allocation sequence**<br>Study vaccine or placebo was allocated to codes through the use of a computerized randomization scheme generator. A detailed randomization standard operating procedure (SOP) was developed prior to study initiation by PATH (Seattle, USA).	Manuscript, Methods section
	8b	**Type of randomisation; details of any restriction (such as blocking and block size)**<br>Regardless of exact plan, an allocation code was randomly assigned to each subject using a method which maintains the 3:1 ratio of study vaccine to placebo in each study cohort; no restrictions.	
Allocation concealment mechanism	9	**Mechanism used to implement the random allocation sequence (such as sequentially numbered containers), describing any steps taken to conceal the sequence until interventions were assigned**<br>According to Randomization standard operating procedure provided by PATH (Seattle, USA).	Protocol S1, Page 29
Implementation	10	**Who generated the random allocation sequence, who enrolled participants, and who assigned participants to interventions**<br>PATH (Seattle, USA) generated random allocation sequence, and Research Institute of Influenza (St Petersburg, Russia) enrolled subjects and assigned participants to intervention arms.	Protocol S1
Blinding	11a	**If done, who was blinded after assignment to interventions (for example, participants, care providers, those assessing outcomes) and how**<br>Participants, care providers, and those assessing outcomes were all blinded until the final database lock; this was done according to the Masking Procedures for Trial Study Protocol LAIV−H7N3−01; version 0.1, 23 Oct 2011 (attached as a supporting information file).	Protocol S1
	11b	**If relevant, description of the similarity of interventions**<br>Both LAIV and placebo were supplied by MICROGEN (Irkutsk, Russia), the manufacturer. Study vaccine was produced as lyophilizate (a light yellow–colored amorphous mass) of live attenuated influenza virus for the preparation of a solution for intranasal introduction. The active component was the vaccine strain A/17/Mallard/Netherlands/00/95(H7N3) virus obtained from the allantoic fluid of chicken embryos. LAIV H7N3 was supplied lyophilized in single–dose ampoules. The lyophilizate also contained the following stabilizers: sucrose, lactose, glycine, sodium glutamate, tris (hydroxymethyl) amino–methane, sodium chloride, and gelatin. Placebo was manufactured to have a similar formulation and presentation as study vaccine. The production process starts with allantoic fluid of chicken embryos that have not been inoculated with any influenza virus. A placebo lyophilizate also contained the same concentrations of stabilizers as LAIV.	Protocol S1, Page 33
Statistical methods	12a	**Statistical methods used to compare groups for primary and secondary outcomes**<br>Percentages of subjects experiencing each reaction or event or at least one reaction or event was calculated along with 95% confidence intervals (CIs) using exact statistical methods.<br>Percentages of subjects with each immune response was calculated along with 95% CIs using exact statistical methods. Geometric mean titres (GMTs) along with 95% CIs were also calculated using the t−test.	Protocol S1, Page 70, 71
	12b	**Methods for additional analyses, such as subgroup analyses and adjusted analyses**<br>No multiplicity adjustment to the error rate, alpha, were made because there were no statistical hypotheses and	

		all analyses were descriptive.	
**Results**			
Participant flow (a diagram is strongly recommended)	13a	**For each group, the numbers of participants who were randomly assigned, received intended treatment, and were analysed for the primary outcome**<br>50 participants were assessed for eligibility, 10 participants were excluded from the study because they didn't meet inclusion criteria. 40 health male and female adult volunteers were included in the full vaccine study. They were randomly divided in two groups (30 received vaccine and 10 placebo) and analyzed for the primary outcome. The flow diagram is attached to the manuscript as Figure 1.	Protocol S1, Page 27
	13b	**For each group, losses and exclusions after randomisation, together with reasons**<br>One subject from vaccine group dropped out from the study prior to receiving the second dose of vaccine because of an adverse event not related to the vaccination (adenovirus infection on Day 28 confirmed by PCR). The flow diagram is attached to the manuscript as Figure 1.	Manuscript, Figure 1
Recruitment	14a	**Dates defining the periods of recruitment and follow–up**<br>April 9, 2012 – June 29, 2012.	http://clinicaltrials.gov/show/NCT01511419
	14b	**Why the trial ended or was stopped**<br>The trial was completed according to the Trial Protocol.	
Baseline data	15	**A table showing baseline demographic and clinical characteristics for each group**<br>40 healthy adult volunteers were recruited for this study.	

		LAIV H7N3	PLACEBO
Age (years)	n	30	10
	Mean (SE)	30.1 (1.88)	38.5 (3.05)
	Median	26.5	43.5
Sex [n(%)]	Female	15 (50)	4 (40)
	Male	15 (50)	6 (60)

Numbers analysed	16	**For each group, number of participants (denominator) included in each analysis and whether the analysis was by original assigned groups**<br>After the first vaccination 30 subjects in vaccine group and 10 subjects in placebo group were included in each analysis. After the second vaccination 29 subjects in vaccine group and 10 subjects in placebo group were included in each analysis.	Manuscript, Figure 1
Outcomes and estimation	17a	**For each primary and secondary outcome, results for each group, and the estimated effect size and its precision (such as 95% confidence interval).**<br>Observed proportions of subjects exhibiting reactions, adverse events and clinical chemistry anomalies, as well as proportions of subjects seroconverting or seropositive for influenza antibodies using various methods, were	Manuscript, Table 4, 5

estimated with 95% confidence intervals. GMTs were estimated with 95% confidence intervals.
For primary outcome see below percentage of adult subjects vaccinated with H7N3 LAIV with solicited local and systemic reactions within 7 days of vaccination.

Reactogenicity event	Treatment group, n (%)			
	LAIV		Placebo	
After dose 1	n = 30	95% CI[3]	n = 10	95% CI
Any solicited local reactions	2 (6.7%)	0.8–22.1	1 (10.0%)	0.3–44.5
Any solicited systemic reactions	11 (36.7%)	19.9–56.1	4 (40.0%)	12.2–73.8
Any solicited local and systemic reactions[1]	11 (36.7%)	19.9–56.1	4 (40.0%)	12.2–73.8
After dose 2	n = 29	95% CI	n = 10	95% CI
Any solicited local reactions	1 (3.4%)	0.1–17.8	0	0
Any solicited systemic reactions	5 (17.2%)	5.8–35.8	1 (10.0%)	0.3–44.5
Any solicited local and systemic reactions[2]	5 (17.2%)	5.8–35.8	1 (10.0%)	0.3–44.5

[1]All reactions observed were mild and included sore throat, fever, nasal congestion and catarrhial nasopharinx, sneeze and headache. [2]All reactions observed were mild and included sore throat, fever, cough and nasal congestion. [3]95% confidence interval.

As for secondary outcome, two–dose immunization of healthy adults with H7N3 LAIV resulted in significant serum antibody responses and generation of $CD4^+$ and $CD8^+$ immunological memory T cells compared to placebo group. Up to 24.1% of subjects from vaccinated group respond with serum antibody conversions after the first dose of LAIV and up to 44.8% after second dose. Local antibody rises were observed in 41.4% of persons after first vaccination and up to 41.4% of subjects responded with increases in H7N3–specific $CD4^+$ and/or $CD8^+$ T cells after two–dose vaccination.
Cumulative data on all antibody and cell immune responses showed that 86.2% of vaccinated subjects had serum and/or local antibodies generated as a result of vaccination.
The results of primary and secondary outcomes demonstrated that H7N3 LAIV was well−tolerated and immunogenic in a Phase I Trial in healthy adults. Phase II study of this preparation may be started for its further registration.

17b **For binary outcomes, presentation of both absolute and relative effect sizes is recommended**
No binary outcomes were reported in the study.

Ancillary analyses 18 **Results of any other analyses performed, including subgroup analyses and adjusted analyses, distinguishing pre–specified from exploratory** — Manuscript, Table 8
In addition to the Trial Study Protocol the study of cross–reactive potential of the H7N3 LAIV against newly emerged H7N9 virus was performed. It was demonstrated that sera from some of the H7N3–vaccinated subjects elicited heterosubtypic antibodies able to neutralize H7N9 virus.

Harms 19 **All important harms or unintended effects in each group**

		No harms or unintended effects in each group were reported.	
**Discussion**			
Limitations	20	**Trial limitations, addressing sources of potential bias, imprecision, and, if relevant, multiplicity of analyses**<br>There were no limitations in the study.	
Generalisability	21	**Generalisability (external validity, applicability) of the trial findings**<br>Not applicable.	
Interpretation	22	**Interpretation consistent with results, balancing benefits and harms, and considering other relevant evidence**<br>It was shown in early publications that pre–pandemic LAIV based on caA/chicken/BC/CN–7/04 H7N3 strain (MEDIMMUNE LAIV) was safe and immunogenic. In our study we demonstrated that the A/mallard/Netherlands/12/2000–based H7N3 LAIV was also safe and immunogenic and replicated better than MEDIMMUNE H7N3 vaccine. In addition, the H7N3 LAIV produced broadly reactive antibodies, which can neutralize the newly emerged H7N9 avian influenza virus. Importantly, the A/mallard/Netherlands/12/2000 virus belongs to the Eurasian lineage of H7 viruses, same as the newly emerged H7N9 strains, whereas H7N3 LAIV strain caA/chicken/BC/CN–7/04 belongs to North American lineage. H7N3 LAIV with cross–reactive potential can be readily used for prime immunization of the population in the face of the H7N9 pandemic.	Manuscript, Discussion section
**Other information**			
Registration	23	**Registration number and name of trial registry**<br>ClinicalTrials.gov NCT01511419.	Manuscript, Methods section
Protocol	24	**Where the full trial protocol can be accessed, if available**<br>The full Trial Study Protocol LAIV−H7N3−01; version 3.0, 11 November 2011 is attached as a supporting information file.	Protocol S1
Funding	25	**Sources of funding and other support (such as supply of drugs), role of funders**<br>The study was supported by PATH. Some staff members from the funder also participated in designing the experiments and critically reviewed the manuscript.	Manuscript, Acknowledge ments section

*We strongly recommend reading this statement in conjunction with the CONSORT 2010 Explanation and Elaboration for important clarifications on all the items. If relevant, we also recommend reading CONSORT extensions for cluster randomised trials, non–inferiority and equivalence trials, non–pharmacological treatments, herbal interventions, and pragmatic trials. Additional extensions are forthcoming: for those and for up to date references relevant to this checklist, see www.consort–statement.org.
