# Supplementary material for: Assessment of Human Immune Responses to H7 Avian Influenza Virus of Pandemic Potential: Results from a Placebo–Controlled, Randomized Double–Blind Phase I Study of Live Attenuated H7N3 Influenza Vaccine
Source: PLoS One. 2014 Feb 12;9(2):e87962. doi: 10.1371/journal.pone.0087962 (PMC3922724; doi:10.1371/journal.pone.0087962)
Supplement: Procedure S1 — Masking procedures for Protocol LAIV-H7N3-01. (PDF) [file pone.0087962.s002.pdf]

# Masking procedures for Protocol LAIV-H7N3-01

**Responsibility**

Two unblinded study clinicians will be responsible for preparation and masking of study vaccine and placebo for this study. They will not be allowed to administer the preparations or interact with participants or conduct any other activities in this study. These unblinded clinicians will not reveal preparation identity to any other study staff or to the subject.

**Acquisition of study vaccine and placebo**

The investigator or study coordinator, if applicable, will be personally responsible for vaccine receipt and management or will designate a person who will be responsible for these activities. PVS (or its designate, PSI) and IEM will determine with the investigator or the person in charge, the date(s) and time(s) of delivery of study vaccine and placebo to the study site.

The person in charge of vaccine receipt will check that the cold chain was maintained during shipment: continuous verification of temperature and/or cold chain monitoring card. In case of any problem, he/she must alert PVS/PSI and IEM and Microgen immediately.

The acknowledgments of receipt of study vaccine and placebo will be dated and signed by the person in charge of vaccine at RII. Copies will be kept for archiving at the investigator's site and copies will be returned to PVS/PSI (for study vaccine and placebo) and IEM.

**Packaging and Labeling**

Study vaccine and placebo will be packaged identically, but each package will clearly identify each study vaccine and placebo as such on the label. Ampoules within each package will be clearly labeled as study vaccine or placebo. Spray devices will be provided unlabeled.

**Storage and Stability**

LAIV H7N3 and placebo should be stored at a temperature between +2 degrees Celsius (°C) to +8°C (in a refrigerator). Storage temperature should be monitored daily and documented on an appropriate form. Back-up power or storage should be available in case of primary power failure. Study vaccine and placebo should never be frozen. The study vaccine or placebo should not be used if the ampoule is broken, the label is illegible or the physical properties (color and transparency) are altered.

In case of accidental disruption of the cold chain, the products should not be administered and the investigator or the responsible person should contact the Sponsor and PVS/PSI to receive further instructions. In such cases, the investigator must receive written consent of the PVS/PSI before any study vaccine or placebo may be used.

## Delivery of Allocation Sequences to Unblinded Study Clinicians

Dr. John Victor, PATH/PVS clinical lead, will deliver the Allocation Sequence in a sealed envelope to the unblinded study clinicians. This Allocation Sequence will be separated into four separate documents, one for each of the following:

- cohort 1, dose 1
- cohort 2, dose 1
- cohort 1, dose 2
- cohort 2, dose 2.

Each document will contain a sequential list of allocation codes (3-digit numbers) matched to a treatment, study vaccine or placebo. On the documents will also be columns for unblinded study clinicians to sign their initials attesting to proper treatment preparation and labeling for each study vaccine or placebo prepared. On the document will also be a column to write the participant's study ID number. An example of such a document is provided here:

**ПРОТОКОЛ ЖГВ-H7N3-01 ПЕРВАЯ доза**

| Code | Allocation | Prepared by | Verified by | Participant ID |
|---|---|---|---|---|
| код препарата | препарат | медсестра, которая подготовила и вводят препарат | Медсестра которые подтвердили, что препарат был назначен правильно заданных. | Участник ID |
| 101 | Live Influenza Vaccine (H7N3)<br>живая гриппозная вакцина | | | |
| 102 | Live Influenza Vaccine (H7N3)<br>живая гриппозная вакцина | | | |
| 103 | Placebo<br>плацебо | | | |
| 104 | Live Influenza Vaccine (H7N3)<br>живая гриппозная вакцина | | | |
| 105 | Live Influenza Vaccine (H7N3)<br>живая гриппозная вакцина | | | |
| 106 | Live Influenza Vaccine (H7N3)<br>живая гриппозная вакцина | | | |
| 107 | Placebo<br>плацебо | | | |
| 108 | Live Influenza Vaccine (H7N3)<br>живая гриппозная вакцина | | | |
| 109 | Live Influenza Vaccine (H7N3)<br>живая гриппозная вакцина | | | |
| 110 | Live Influenza Vaccine (H7N3)<br>живая гриппозная вакцина | | | |
| 111 | Live Influenza Vaccine (H7N3)<br>живая гриппозная вакцина | | | |
| 112 | Placebo<br>плацебо | | | |

## Post-preparation Labels

PVS/PSI will print post-preparation labels (stickers) for 40 3-digit allocation codes, from 101 to 140, and provide them to the two unblinded study clinicians. Each set of labels for each allocation code will have sufficient identical stickers in order to label two prepared spray devices for inhalation (one for each dose in the study), two ampoules of study vaccine or placebo (one for each dose in the study), and two appropriate locations in the CRF. Post-preparation labels will not identify study treatment.

## Assignment of Allocation Code to each Study Participant

*To preserve the randomization scheme, allocations codes must be sequentially assigned to study participants, with study participants selected for allocation consecutively based on their study identification number.* Allocation codes and study participant numbers will not necessarily appear to match for all subjects. For instance, if a study participant with subject ID number 01-04 is successfully enrolled but withdraws prior to allocation to treatment, the study participant's identification number will be skipped when allocation is considered. Graphically this is as follows:

| Allocation Code | Subject ID number |
|---|---|
| 101 | 01-01 |
| 102 | 01-02 |
| 103 | 01-03 |
| 104 | 01-05 |
| 105 | 01-06 |
| etc | Etc |

Before preparation of study vaccine or placebo, a blinded clinician will inform an unblinded clinician of the next consecutive subject ID number for the next participant for assignment to treatment. The unblinded clinician will then obtain the proper study treatment based on the proper Allocation Sequence document. Post-preparation labels with the proper allocation code will also be selected.

## Dosage, Preparation and Administration of Study Products

## Dosage

Dosage of study vaccine or placebo will be two doses given 28 days apart. Each dose will be a total of 0.5 ml of prepared study vaccine or placebo, with 0.25 ml delivered intranasally into each nasal passage using a spray dosing device.

## Preparation and Administration

Preparation of study vaccine or placebo and filling of spray dosing devices will be done by the unblinded clinician behind a screen or in a separate room from where blinded clinicians and study participants will be located. For each intranasal spray, filled spray devices will be carefully handed to the blinded study clinician administering the preparation.

Preparation of study vaccine and placebo and filling of spray dosing devices will be closely monitored to assure that the randomization scheme and study blinding are not broken. Study vaccine or placebo given to each individual subject will be documented on the CRF with the appropriate allocation code.

Directly before inoculation of study vaccine or placebo, the contents of an ampoule must be dissolved in 0.5 ml of water for injections for 3 minutes. The dissolved product should be used within 30 minutes.

For preparation and intranasal administration, the spray dosing device with disposable applicator provided in the packaging will be used. The spray dosing device consists of a sterile syringe with scales of 40 and 100 units (GOST R ISO 10993, GOST 24861-91, GOST 25026-81), a sterile needle, and one adjustable end cap actuator-183.016 for the creation of a fine spray mist.

The study vaccine or placebo will then be prepared by an unblinded study clinician as follows (the other unblinded study clinician will assist by verifying the process):

1. The needle will be placed on the syringe, and 0.5 ml of water for injections will be drawn (to the mark of 20 on the scale of 40 units or to the mark of 50 on the scale of 100 units).
2. The ampoule of study vaccine or placebo will be opened, and the 0.5 ml of water added from the syringe.
3. After 3 minutes for dissolution of study vaccine or placebo, 0.25 ml of the now-liquid preparation will be drawn into the syringe (to the mark of 10 on the scale of 40 units or the mark of 25 on the scale of 100 units).
4. The needle will be removed and placed on a clean or sterile surface, and the spray actuator will be place tightly on the syringe.
5. Using two of the post-preparation label stickers, the syringe and ampoule will be both be labeled.

The unblinded study clinician will then hand-deliver the prepared syringe with proper treatment to a blinded study clinician who will continue the process as follows:

6. With the subject seated in a comfortable position with the head slightly tilted back, the tip of the spray actuator will be brought up to the subject's **right** nostril and inserted 0.5 centimeters (cm) into the nasal passage, and the hub of the syringe strongly depressed to inject the product into the nasal passage. After the spray of vaccine into the nostril, the subject should remain in the seated position with the head slightly tilted back for 1 minute.

After the first intranasal spray is administered, the blinded study clinician will hand-deliver the prepared treatment syringe back to the unblinded study clinician who will refill the sprayer as follows:

7. After the first spray, the spray actuator will be removed and placed on a clean or sterile surface, and the needle will be replaced on the syringe. Another 0.25 ml of liquid preparation will be drawn into the syringe.
8. The needle will once again be removed, and the spray actuator again placed on the syringe.

The unblinded study clinician will then again hand-deliver the prepared syringe (and a post-preparation sticker to attach to the appropriate place in the CRF) to the blinded study clinician who will complete the intranasal administration of treatment as follows:

9. With the subject seated in a comfortable position with the head slightly tilted back, the tip of the spray actuator will be brought up to the subject's **left** nostril and inserted 0.5 cm into the nasal passage, and the hub of the syringe strongly depressed to inject the product into the nasal passage. Again, after the spray of vaccine into the nostril, the subject should remain in the seated position with the head slightly tilted back for 1 minute.

The blinded study clinician will then return the used syringe back to the unblinded study clinician who will maintain all used materials for later monitoring by PVS/PSI. The unblinded study clinicians will initial the appropriate places on the Allocation Sequence document.

**Accountability Procedures for Study Products**

Study vaccine and placebo must be kept in a secure place. The investigator or the person in charge of study product management will maintain records of delivery of the products to the trial site, the inventory at the site, the dose(s) given to each participant, and the destruction or return of unused doses.

**Return of Allocation Sequence documents**

After preparation, labeling and administration of all doses of study vaccine and placebo to all participants, the four completed documents with allocation numbers should be placed in an envelope and sealed and signed. The sealed envelope should be provided to the unblinded PSI study monitor for return to PATH.
