# Supplementary material for: Assessment of Human Immune Responses to H7 Avian Influenza Virus of Pandemic Potential: Results from a Placebo–Controlled, Randomized Double–Blind Phase I Study of Live Attenuated H7N3 Influenza Vaccine
Source: PLoS One. 2014 Feb 12;9(2):e87962. doi: 10.1371/journal.pone.0087962 (PMC3922724; doi:10.1371/journal.pone.0087962)
Supplement: Protocol S1 — Protocol LAIV-H7N3-01 for clinical trial “Reactogenicity, safety, and immunogenicity of a live monovalent A/17/mallard/Netherlands/00/95 (H7N3) influenza vaccine”. (PDF) [file pone.0087962.s003.pdf]

REACTOGENICITY, SAFETY, AND IMMUNOGENICITY OF A LIVE MONOVALENT A/17/MALLARD/NETHERLANDS/00/95 (H7N3) INFLUENZA VACCINE

Protocol Number: LAIV-H7N3-01

Sponsor: Federal State Unitary Company "Microgen Scientific Industrial Company for Immunobiological Medicines" of the Ministry of Health and Social Development of the Russian Federation

Funding Agency: PATH Vaccine Solutions

Primary Investigator: Prof. Oleg I. Kiselev

Federal State Government-financed Institution "Research Institute of Influenza"
Ministry of Healthcare and Social Development of the Russian Federation

Version Number: 3.0

Date: 11 November 2011

STATEMENT OF COMPLIANCE

This study will be carried out in accordance with Good Clinical Practice (GCP) as required by applicable rules of the Russian Federation: "Rules of the Quality of Clinical Trials in the Russian Federation", OST 42-511-1999, and "Good Clinical Practice", OST 52-379-2005, and the federal law "On the Circulation of Drugs" of April 12, 2010, #61-FZ.

The study informed consent documents will embody the elements of consent as described in the Declaration of Helsinki.

All key personnel (all investigators responsible for the design and conduct of this study) will have completed Human Subjects Protection Training prior to interaction with any participants or to having access to their confidential study data.

SIGNATURE PAGE

The signatures below constitute the approval of this protocol and all attachments and provide the necessary assurances that this trial will be conducted according to all stipulations of the protocol, including all statements regarding confidentiality, and according to local legal and regulatory requirements and applicable guidelines.

By signing below, I agree:

1. to assume responsibility for the proper conduct of the trial at this center.
2. that I am familiar with and will comply with the guidelines on Good Clinical Practice (GCP) and all the applicable regulatory requirements.
3. to conduct the current trial in accordance with this protocol, along with any amendments to the protocol adopted in the future and with any procedures for the conduct of the trial, proposed by the Sponsor and PVS.
4. to guarantee that all personnel assisting me in the trial are sufficiently informed on the trial products and other responsibilities and functions outlined in the protocol.
5. to not make any changes to the protocol without the consent of the Sponsor and PVS, and also preliminary acquaintance with them and their written approval by the Ethics Committee under the Federal Agency for Quality Control of Medicines, except in situations involving the need for the immediate reduction of risk to trial participants.
6. that I am thoroughly acquainted with the proper rules for the use of the products described in this Protocol, and any other information provided by the Sponsor and PVS.
7. that I have been informed about the conditions for confidentiality of information, including medical, clinical or other data, and also information concerning the Sponsor's and PVS's business.
8. that all the data, documents, any records and information transmitted by the Sponsor or obtained or prepared by me, by my personnel or my consultants during the trials belongs to the Sponsor, IEM, and PVS.
9. that I, my personnel and my consultants or other staff persons participating in the trial do not have the right to publish any data related to or obtained during the clinical trial without first following the agreed upon publication rules agreed to contractually.
10. that I, my personnel and my consultants or other staff persons participating in the trial do not have the right to use the name of the Sponsor or PVS in any advertising or promotional sales materials without the prior written consent of the Sponsor or PVS, respectively.
11. to provide a version of my biography and all other documents required by the Sponsor

Signed: Date:

11 November 2011

Professor Oleg I. Kiselev, MD, PhD, DSc
Director, Research Institute of Influenza

Day/Month/Year

TABLE OF CONTENTS

Protocol Summary viii

1 Key Roles 16
2 Background Information and Scientific Rationale 19
2.1 Background Information 19
2.2 Dose Rationale 21
2.3 Potential Risks and Benefits 21
2.3.1 Potential Risks 21
2.3.2 Known Potential Benefits 23
3 Objectives 23
3.1 Study Objectives 23
3.1.1 Primary Objective 23
3.1.2 Secondary and Exploratory Objectives: 23
3.2 Study Outcome Measures 23
3.2.1 Primary Outcome Measures 23
3.2.2 Secondary Outcome Measures 24
3.2.3 Exploratory Outcome Measures 24
4 Study Design 25
5 Study Enrollment and Withdrawal 27
5.1 Subject Inclusion Criteria 27
5.2 Subject Exclusion Criteria 27
5.3 Treatment Assignment Procedures 29
5.3.1 Randomization Procedures 29
5.3.2 Masking Procedures 29
5.4 Subject Withdrawal from the Trial 30
5.4.1 Reasons for Withdrawal (Premature Termination) 30
5.4.2 Withdrawals at Specific Time-Points 30
5.4.3 Handling of Withdrawals 31
5.5 Termination of the Trial 32
5.5.1 Termination According to the Protocol 32
5.5.2 Suspension and/or Premature Termination of the Trial 32
6 Study Products 33
6.1 Study Product Descriptions 33
6.1.1 Study Vaccine 33
6.1.2 Placebo 33
6.1.3 Acquisition 33
6.1.4 Packaging and Labeling 34
6.1.5 Storage and Stability 34
6.2 Dosage, Preparation and Administration of Study Products 34
6.2.1 Dosage 34
6.2.2 Preparation and Administration 34
6.3 Accountability Procedures for Study Products 35
6.4 Concomitant and Unauthorized Products 35

6.4.1 Concomitant Medications/Treatment 35
6.4.2 Unauthorized Products 36
6.4.3 Use of Unauthorized Products or Products Not Stipulated by the Protocol 36
7 Study Schedule 36
7.1 Screening 36
7.1.1 First Screening Visit (14 to 4 Days Prior to Trial Enrollment, Day S1) 37
7.1.2 Second Screening Visit (Day S2) / Trial Enrollment Day (Day 0) 37
7.2 Vaccination and Follow-up Periods 38
7.2.1 Day of First Admission into the Isolation Unit (Day 0) 38
7.2.2 First Day after Administration of Dose One (Day 1) 39
7.2.3 Second Day after Administration of Dose One (Day 2) 39
7.2.4 Third Day after Administration of Dose One (Day 3) 39
7.2.5 Fourth Day after Administration of Dose One (Day 4) 39
7.2.6 Fifth Day after Administration of Dose One (Day 5) 39
7.2.7 Sixth Day after Administration of Dose One (Day 6) 39
7.2.8 Seventh Day after Administration of Dose One (Day 7) 39
7.2.9 Days after First Discharge from the Isolation Unit (Days 8 to 27) 41
7.2.10 Day of Second Admission into the Isolation Unit (Day 28) 41
7.2.11 First Day after Administration of Dose Two (Day 29) 42
7.2.12 Second Day after Administration of Dose Two (Day 30) 43
7.2.13 Third Day after Administration of Dose Two (Day 31) 43
7.2.14 Fourth Day after Administration of Dose Two (Day 32) 43
7.2.15 Fifth Day after Administration of Dose Two (Day 33) 43
7.2.16 Sixth Day after Administration of Dose Two (Day 34) 43
7.2.17 Seventh Day after Administration of Dose Two (Day 35) 43
7.2.18 Days after Second Discharge from the Isolation Unit (Days 36 to 55) 44
7.2.19 Day of Final Visit to the Study Center (Day 56) 44
7.3 Unscheduled Visits 45
8 Study Evaluations 45
8.1 Clinical Evaluations 45
8.1.1 Definition and Categorization of AEs 45
8.1.2 Specific Clinical Signs and Symptoms of Interest 45
8.1.3 Medical History 46
8.1.4 Physical Examination 47
8.1.5 ENT Examination 47
8.2 Laboratory Evaluations 48
8.2.1 Clinical Laboratory Evaluations 48
8.2.2 Special Assays 50
8.2.3 Nasal and Conjunctival Swab Specimens 56
8.2.4 Nasal Wick Specimens 57
8.2.5 Blood Specimens 57
8.2.6 Urine Specimens 60
9 Assessment of Safety 60
9.1 Specification of Safety Parameters 60
9.2 Methods and Timing for Assessing and Recording Safety Parameters 61

9.2.1 Post-administration Reactions 61
9.2.2 Adverse Events 62
9.2.3 Serious Adverse Events 63
9.2.4 Procedures to be Followed in the Event that of Abnormal Laboratory Test Values 64
9.3 Reporting Procedures 65
9.3.1 Serious Adverse Events 65
9.3.2 Regulatory Reporting 65
9.3.3 Reporting of Pregnancy 65
9.4 Type and Duration of Follow-up of Subjects after Adverse Events 65
9.5 Halting Rules 66
9.6 Safety Oversight by a Safety Monitoring Committee 66
9.6.1 SMC Roles and Responsibilities 66
9.6.2 Study Materials for SMC Review 67
9.6.3 Reports from the SMC 67
10 Clinical Monitoring 68
10.1 Site Monitoring Plan 68
10.1.1 Set-up Visit 68
10.1.2 Follow-up Visits 68
10.1.3 Close-out Visit 69
10.2 Audits and Inspections 69
10.3 Archiving 69
11 Statistical Considerations 70
11.1 Study Hypotheses 70
11.2 Definition of Analysis Sets 70
11.3 Analysis Plan 70
11.3.1 Safety 70
11.3.2 Immunogenicity 71
12 Source Documents and Source Document Access 71
13 Quality Control and Quality Assurance 72
14 Ethics/Protection of Human Subjects 72
14.1 Ethical Standard 72
14.2 Institutional Review Boards and Independent Ethics Committee 72
14.3 Informed Consent Process 73
14.4 Inclusion of Women, Minorities and Children 74
14.5 Insurance 74
14.6 Financing 75
14.7 Subject Confidentiality 75
14.7.1 Confidentiality of Data 75
14.7.2 Confidentiality of Patient Records 75
14.7.3 Notification of Primary Care Physician 76
14.8 Study Discontinuation 76
14.9 Sharing of Study Results with Subjects 76
14.10 Future Use of Stored Specimens 76
15 Data Handling and Record Keeping 77
15.1 Data Management Responsibilities 77

15.2 Data Capture Methods 77
15.3 Types of Data 78
15.4 Timing/Reports 78
15.5 Study Records Retention 78
15.6 Protocol Deviations 78
16 Final Report on the Trial and Publication Policy 79
17 Literature References 80
18 Attachments 82

PROTOCOL SUMMARY

Title: Reactogenicity, safety and immunogenicity of a live monovalent A/17/Mallard/Netherlands/00/95 (H7N3) influenza vaccine

Phase: I

Study Population: 40 healthy male and female adults, 18 to 49 years of age.

Number of Sites: Study is single-site and single-center.

Study Duration: Approximately 3 months.

Participation Duration: Each participant will be enrolled for approximately 10 weeks.

Description of Agent or Intervention: Monovalent H7N3 live monovalent influenza vaccine (LAIV H7N3), (FSUC «Microgen SIC for Immunobiological Medicines» of the Ministry of Health and Social Development of the Russian Federation. Moscow) or matched placebo (FSUC «Microgen SIC for Immunobiological Medicines» of the Ministry of Health and Social Development of the Russian Federation. Moscow)*)*. Subjects will receive two, 0.50 ml intranasal doses of study vaccine or placebo at study entry and four weeks post-dose one.

Objectives: **Primary Objective:** To describe the safety profile of two intranasal doses of LAIV A/17/Mallard/Netherlands/00/95 (H7N3) in healthy adults.

The safety profile will be parameterized as the proportion of subjects experiencing adverse events (AEs) of the following four categories:

- Immediate reactions occurring within two hours of administration of any dose, measured as observed by study staff or reported by the subject to study staff.
- Adverse events commonly associated with intranasal vaccination (solicited local and systemic reactions) occurring greater than two hours after administration of any dose of study vaccine or placebo through 7 days following any dose, measured as observed by study staff or reported by the subject to study staff.
- All other adverse events (including unsolicited events) occurring during the 7 days following any dose, measured as observed by study staff or reported by the subject to study staff. This includes abnormal

Objectives, cont.:

laboratory findings from blood and urine specimens collected on Days 7 and 35.

- All serious adverse events (SAEs) occurring within 4 weeks of receipt of any dose, as observed by study staff, reported by the subject to study staff, or noted by the subject on a diary card. This includes abnormal laboratory findings from blood and urine specimens collected on Days 28 (pre-vaccination) and 56.

Secondary Objectives:
To describe the post-vaccination immune responses to influenza vaccine and virus shedding by the following:

- Serum hemagglutination-inhibition antibodies
- Serum neutralizing antibodies using microneutralization assay
- Serum immunoglobulin class A (IgA) or class G (IgG) antibodies using enzyme-linked immunoassay (EIA)
- Mucosal IgA antibodies in nasal wick specimen
- Virus shedding with virus detected by real-time reverse transcriptase polymerase chain reaction rRTPCR in nasal swabs or conjunctival swabs or by isolation in chicken embryos at any time-point.
- Virus infectivity and stability (virus detected and sequenced after inoculation into chicken eggs)

Immune responses and virus shedding will be assessed after each dose. Besides, cellular immune responses (cytokines and T cells) will also be assessed by analysis of peripheral blood lymphocytes using flow cytometry and ELISPOT techniques.

Description of Study Design:

This is a phase I, double-blind, individually-randomized (3:1, vaccine:placebo), controlled trial with two groups, LAIV H7N3 and matched placebo. Healthy male and female adults 18 through 49 years of age will be invited to participate. For feasibility reasons and in order for an independent Safety Monitoring Committee (SMC) to review safety data in a small group of subjects initially, the total cohort of 40 subjects will be enrolled in two sub-cohorts: one cohort of 12 subjects, randomized at 3:1 (9 vaccine and 3 placebo), followed two weeks later by a second cohort of 28 subjects randomized at 3:1 (21 vaccine and 7 placebo). After all 12 volunteers of the

Description of Study Design, cont.:

first sub-cohort have been observed for the first isolation period (Day 1 to Day 7), an interim safety review will be performed by the SMC. The SMC will review all AEs, including clinical laboratory evaluations (pre- and post-vaccination) and shedding data, for all subjects and will advise if the volunteers of the first sub-cohort may receive dose two of study vaccine or placebo and if the additional 28 volunteers of the second sub-cohort may be enrolled into the study. As for the first sub-cohort, the SMC will also review all safety data for the second sub-cohort and for the entire participant population of the trial. For each sub-cohort, the procedures and timelines are here summarized.

On the day of first screening (S1), about 7 days (between 4 and 14 days) prior to administration of dose one of study vaccine or placebo, subjects will be screened for eligibility through medical history review, physical examination, testing for serologic evidence of chronic viral infection [human immunodeficiency virus (HIV), hepatitis B virus (HBV) or hepatitis C virus (HCV)], routine biochemical and hematological blood tests and urinalysis by dipstick.

Subject screening for eligibility will continue and be completed on the second screening day (S2). This second screening day will occur the same day as scheduled admission to the isolation unit and administration of study vaccine or placebo (Day 0). Women will undergo pregnancy tests using urine samples. All subjects will undergo an ear, nose and throat (ENT) examination. Fully eligible subjects will be admitted to the isolation unit. At that time, nasal swab, nasal wick, and blood specimens will be collected for virologic and immunological testing prior to administration of study vaccine or placebo. Blood and urine specimens will be again collected for routine biochemical and hematological blood tests and urinalysis by dipstick; these results will serve to define baseline status for subject prior to receipt of study vaccine or placebo but will not be used for screening purposes. Subjects and investigators conducting assessments of safety will be unaware of which allocation, LAIV H7N3 or matched placebo, is received; study vaccine and placebo will be masked. Subjects will be carefully monitored for adverse reactions while in the isolation unit.

All subjects will remain in the isolation unit for at least 7 days after receipt of study vaccine or placebo. Nasal swabs will be collected daily while subjects are in isolation to test for

Description of Study Design, cont.:

presence of influenza virus shed in the nasal passage. Any subject exhibiting conjunctivitis will also have a conjunctival swab collected on the day of appearance of the sign. Any subject exhibiting influenza A virus shedding, as determined by real-time RT-PCR positivity on a nasal swab specimen, in the 2 days prior to each planned discharge day after each dose (Days 6 or 7 or Days 34 or 35) will be kept in the isolation until PCR-diagnosis results confirm that no influenza virus is present in a tested clinical specimen for at least two consecutive days.

Any subject still exhibiting evidence of influenza virus shedding in a nasal swab on Days 6 or 7 or Days 34 or 35 post-administration with each dose might be placed on influenza antiviral (oseltamivir) treatment at the standard dose for treatment of 75 milligrams (mg) twice a day for a course of 5 days.

After discharge from the isolation unit, subjects will complete diary cards for AEs and use of concomitant medications. Subjects will return to the isolation unit at four weeks (Day 28) after administration of dose one of study vaccine or placebo. At that time, similar procedures will be used for admittance to the isolation unit, for receipt of dose two of study vaccine or placebo and for isolation and follow-up, with the additional procedure of review of interim histories (and diary cards) since first discharge after dose one.

After second discharge from the isolation unit, subjects will again complete diary cards for AEs and use of concomitant medications. Subjects will then return to the study center at four weeks (Day 56) after administration of dose two of study vaccine or placebo for their final study visit. Interim histories (and diary cards) will again be reviewed and final blood and nasal wick specimens will be collected. Women will also undergo a final pregnancy screen. Subjects will complete the study at this time.

For assessment of safety, subjects will be observed for two hours after each administration of study vaccine or placebo. Twice daily (early morning and late afternoon) examination will be also used to assess reactions for 7 days after each administration of study vaccine or placebo. ENT examination will also occur once per day on Days 7, 28, 35 and 56. Subjects will complete diary cards for unsolicited AEs from the day of each discharge until return to the isolation unit for dose two (at Day 28) or until return to the study center for the

Description of Study Design, cont.:

final study visit at four weeks post dose two (at Day 56). To assess safety, blood and urine specimens will also be collected on days 7, 28 (prior to administration of dose two of study vaccine or placebo), 35 and 56, for testing by routine biochemical and hematological blood tests and by urinalysis by dipstick.

For the evaluation of mucosal IgA antibody, nasal wick specimens will be collected on Day 0 (prior to administration of dose one of study vaccine or placebo), on Day 28 (prior to administration of dose two of study vaccine or placebo) and on Day 56. For the evaluation of serum antibodies (by HAI, microneutralization and IgA and IgG EIA), serum specimens will be collected on Day 0 (prior to administration of dose one of study vaccine or placebo), on Day 28 (prior to administration of dose two of study vaccine or placebo) and on Day 56. To study virus infectivity (by isolation in chicken embryos) and viral genetic stability (by molecular sequencing of any isolated virus), nasal swab specimens will be taken on Days 1, 2, 3, 5, 7, 29, and 31. To assess priming and stimulation of cytotoxic T lymphocytes and other cytokine indicators, whole blood for isolation of PBMCs will be collected on Days 0 (prior to administration of dose one of study vaccine or placebo), on Day 28 (prior to administration of dose two of study vaccine or placebo) and on Day 56.

Estimated Time to Complete Enrollment:

Full enrollment in the study is anticipated within 4 weeks of study initiation.

Statistical Analyses:

No formal statistical hypotheses are proposed for this study. Observed proportions of subjects exhibiting reactions, adverse events and clinical chemistry anomalies, as well as proportions of subjects seroconverting or seropositive for influenza antibodies using various methods, will estimated with 95% confidence intervals. Geometric mean titers (GMTs) will be estimated with 95% confidence intervals.

Trial Scheme

Study Activities (for each sub-cohort)	S1 (-14 to -4 before D0)	Study Day (in number of days from day of administration of dose one or D0)													
		S2/D0	D1	D2	D3	D4	D5	D6	D7	D8 to D27	D28	D29 to D34*	D35	D36 to D55	D56
Information process and written informed consent	X (ICF A)	X (ICF B)													
Collect baseline demographic data	X														
Collect/review medical history	X										X				
Perform targeted physical examination	X	X	X	X	X	X	X	X	X		X	X	X		X
Perform ear nose and throat examination		X							X		X		X		X
Collect serum and whole blood for comprehensive metabolic panel and complete blood count with differential	X	X							X		X		X		X
Collect serum for HIV, HBV and HCV testing	X														
Collect urine for urinalysis via dipstick	X	X							X		X		X		X
Perform pregnancy check (women)		X									X				X
Check/confirm inclusion/exclusion criteria	X	X													
Admission to isolation unit		X									X				
Collect serum for influenza serology (pre-vaccination on days of study vaccination)		X									X				X
Collect blood for isolation of PBMCs		X									X				X
Collect nasal swabs for rRTPCR testing (at 2 hours pre-vaccination and at 6 hours post-vaccination on days of study vaccination)		X	X	X	X	X	X	X	X	*X***	X	X	X	*X***	
Nasal swabs used for isolation of shed virus and subsequent characterization by sequencing			X	X	X		X		X			only D29, D31			
Collect nasal wick specimens for IgA assay (at 1 hour pre-vaccination on days of study vaccination)		X									X				X
**Administer one dose of study vaccine or placebo**		**X**									**X**				
Observe for immediate reactions for 2 hours		X									X				
Record local and systemic reactions		X	X	X	X	X	X	X	X		X	X	X		
Instruct participant on use of diary card									X				X		
Participant records unsolicited AEs									X	X	X		X	X	X
Possible oseltamivir antiviral treatment									*X****				*X****		
Contingency to remain in isolation if nasal swabs (Day 6, 7, 34, or 35) are not free of influenza A virus										*X*				*X*	
Discharge from isolation unit									X				X		
Review interim adverse events****											X				X
Report SAEs		X	X	X	X	X	X	X	X	X	X	X	X	X	X
Subject completion of study															X

*Some screening activities will be repeated on D28. All D0 to D7 activities will be identically repeated from D28 to D35.

**Nasal swab collection will continue for each day subject remains in isolation after planned discharge day. Planned discharge day is D7 post-dose one and D35 post-dose two.

***For subjects with evidence of viral shedding in nasal swabs during the final two days of each stay in the isolation unit (D6 or D7 post-dose one and D34 or D35 post-dose two).

****Safety Monitoring Committtee review on or soon after Days 8 and 36 for each sub-cohort.

LIST OF ABBREVIATIONS

AE	Adverse Event
ALP	Alkaline Phosphatase
ALT	Alanine Aminotransferase
AST	Aspartate Aminotransferase
BUN	Blood Urea Nitrogen
CBC	Complete Blood Count
CI	Confidence Interval
cm	centimeter
CMP	Comprehensive Metabolic Panel
CRF	Case Report Form
°C	Degrees Celsius
D	Day
EDTA	Ethylenediaminetetraacetic acid
EIA	Enzyme Immunoassay
EID	Egg Infectious Dose
ELISPOT	Enzyme-Linked Immunosorbent Spot
ENT	Ear Nose and Throat
FSUE	Federal State Unitary Enterprise
GCP	Good Clinical Practice
GMT	Geometric Mean Titer
HA	Hemagglutinin
HAI	Hemagglutination Inhibition
HBsAg	Hepatitis B Surface Antigen
HBV	Hepatitis B Virus
hCG	human Chorionic Gonadotropin
HCV	Hepatitis C Virus
HIV	Human Immunodeficiency Virus
ICF	Informed Consent Form
ICH	International Conference on Harmonization
ICMJE	International Committee of Medical Journal Editors
IEC	Independent Ethics Committee
IEM	Institute for Experimental Medicine
IgA	Immunoglobulin class A
IgG	Immunoglobulin class G
IRB	Institutional Review Board
l	liter
LAIV	Live Attenuated Influenza Vaccine
MDCK	Madin-Darby Canine Kidney
mg	milligram
ml	milliliter
mm	millimeter
MoH	Ministry of Health and Social Development
MoP	Manual of Procedures
n	number (typically refers to number of subjects)

NA	Neuraminidase
PBMC	Peripheral Blood Mononuclear Cell
PI	Principal Investigator
PCR	Polymerase Chain Reaction
PVS	PATH Vaccine Solutions
RBC	Red Blood Cell
RF	Russian Federation
RII	Research Institute of Influenza
RNA	Ribonucleic Acid
rRTPCR	Real-time Reverse Transcriptase Polymerase Chain Reaction
SAE	Serious Adverse Event
SMC	Safety Monitoring Committee
SOP	Standard Operating Procedure
TCID	Tissue Culture Infectious Dose
US	United States
WBC	White Blood Cell
WHO	World Health Organization

1 Key Roles

Individuals

Microgen Clinical Lead:
D.S. Bushmenkov, Deputy Head of Clinical Research Administration and Authorization
Microgen SIC
10, 2nd Volkonsky Lane,
Moscow, Russia 127473
phone: +7 (495) 790.7773 x2098
mobile: +7 (916) 641.8104
fax: +7 (495) 783.8804
email: d.s.bushmenkov@microgen.ru

PVS Clinical Lead:
John C. Victor, PhD, MPH
Program for Appropriate Technology in Health (PATH)
2201 Westlake Ave, Suite 200
Seattle, WA 98121, USA
phone: +1 (206) 285.3500
fax: +1 (206) 285.6619
mobile: +1 (206) 427.9887
email: cvictor@path.org

IEM Laboratory Scientist / Study Coordinator:
Andrey R. Rekstin, PhD
Institute of Experimental Medicine
Ulitsa Akademika Pavlova 12
St. Petersburg, Russia 197376
phone/fax: +7 (812) 234.9214
mobile: +7 (911) 732.1842
email: arekstin@yandex.ru

RII Principal Investigator:
Oleg I. Kiselev, MD, PhD, DSc
Research Institute of Influenza
Ulitsa Professora Popova 15/17
St. Petersburg, Russia 197376
phone/fax: +7 (812) 234.6200
mobile: +7 (812) 996.6804
email: office@influenza.spb.ru

RII Research Supervisor:
Mariana K. Yerofeyeva, MD, PhD, DSc
Research Institute of Influenza
Ulitsa Professora Popova 15/17
St. Petersburg, Russia 197376

phone/fax: +7 (812) 234.6032
mobile: +7 (905) 265.9109
email: erofeeva@influenza.spb.ru

RII Research Coordinator:
Marina A. Stukova, MD, PhD
Research Institute of Influenza
Ulitsa Professora Popova 15/17
St. Petersburg, Russia 197376
phone/fax: +7 (812) 234.4251
mobile: +7 (921) 308.9707
email: stukova@influenza.spb.ru

Institutions

Sponsor:
Federal State Unitary Company «Microgen Scientific Industrial Company for Immunobiological Medicines» of the Ministry of Health and Social Development of the Russian Federation
15, 1st Dubrovskaya Ulitsa, Moscow, Russia 115088
Contact: Igor Victorevich
phone: +7 (495) 790.7773 x2005
email: i.v.krasilnikov@microgen.ru or igor.krasilnikov@biorosinfo.ru

Funder:
PATH Vaccine Solutions
2201 Westlake Ave, Suite 200
Seattle, WA 98121 USA
Contact: Kathleen M. Neuzil
phone: +1 (206) 285.3500
fax: +1 (206) 285.6619
email: kneuzil@path.org

Research Site and Clinical Laboratory:
Research Institute of Influenza
Ulitsa Professora Popova 15/17
St Petersburg, Russia 197376
Contact: Lyudmila M. Tsybalova, PhD, DMS
phone: +7 (812) 234.6261
email: sovet@influenza.spb.ru

Laboratory Responsible for Immunology and Virology:
Institute of Experimental Medicine
Ulitsa Akademika Pavlova 12
St Petersburg, Russia 197376
Contact: Larisa G. Rudenko, MD, PhD, DSc
phone/fax: +7 (812) 234.9214
mobile: +61 (41) 1070.6698
email: vaccine@mail.ru

**Institutional Review Boards:**	Western Institutional Review Board<br>P.O. Box 12029<br>Olympia, WA 98508-2029<br>phone: +1 360 252.2500 or +1 800 562.4789<br>fax: +1 360 252.2498<br>email: clientservices@wirb.com
	Ethics Committee of the Research Institute of Influenza<br>Ulitsa Professora Popova 15/17<br>St Petersburg, Russia 197376<br>Contact: Velichka Maksakova, PhD (secretary)<br>phone/fax: +7 (812) 234.6032<br>mobile : +7 (909) 584.6680<br>email: maksakova_v@mail.ru
**Independent Ethics Committee:**	Ethics Committee of Ministry of Healthcare and Social Development of the Russian Federation<br>Petrovskiy Ave., 8<br>Moscow, Russia 127051<br>Contact: Elena Baibarina, Head of the Ethics Committee<br>phone: +7 (495) 628 44 53 or +7 (495) 627 29 44

2 Background Information and Scientific Rationale

2.1 Background Information

Vaccines are the most important intervention for the control and prevention of influenza and for reducing morbidity and mortality. Effective vaccines against avian A (H5N1) and other avian influenza viruses are a high public health priority.[1] Production of live-attenuated and inactivated vaccine seed viruses against avian influenza viruses, which have the potential to cause pandemics, may establish proof of concept and will increase manufacturing experience that will be critical in the event of the emergence of such a virus into the human population. The observed efficacy of live-attenuated vaccines for human inter-pandemic influenza, together with findings to date that inactivated or subunit avian influenza vaccines are suboptimally immunogenic in humans, strongly suggest that use of live-attenuated influenza vaccines (LAIVs) against pandemic influenza are worth exploring.[2] LAIVs have higher yields and simpler manufacturing processes as compared to inactivated vaccines, may provide superior immunogenicity in seronegative populations, and have the potential to elicit broader immune responses than inactivated or subunit vaccines.[3] Since LAIVs are generally delivered via intranasal inoculation, another appealing aspect of this approach in a pandemic situation is a simplified, needle-free delivery system. Because LAIV technology requires less complex downstream processing than inactivated vaccines, WHO considers LAIVs to be possibly more appropriate for production of pandemic influenza vaccines and thus, the WHO Global Action Plan encourages increased development, production and technology transfer of LAIV.[4]

From the policy perspective, the decision to use a live-attenuated vaccine prior to a pandemic would be partially influenced by characteristics of the vaccine strain and concerns for reassortment with circulating influenza strains. Such a reassortment event would be particularly concerning for an A/H5N1 strain, for example, which has a demonstrated high case-fatality rate, but is not yet circulating widely and has not acquired the ability to transmit efficiently from person-to-person. Thus, it is important to understand the characteristics of potential pandemic vaccines based on avian strains, to understand their immunogenicity and reactogenicity in humans, and to understand their shedding characteristics, the latter of which could influence their propensity to reassort with circulating human strains.

LAIVs have been used for seasonal influenza vaccines for over 40 years in Russia. The Institute of Experimental Medicine (IEM) in St. Petersburg has been collaborating with PATH Vaccine Solutions (PVS), a nonprofit corporation that is a supporting organization of the nongovernmental global health organization, PATH, to develop prototype live-attenuated cold-adapted reassortant pandemic influenza vaccines based on avian strains. The overall plan includes the generation of a set of live-attenuated cold-adapted reassortant viruses bearing an H5N1 and H7N3 on the attenuated A/Leningrad/134/17/57 (H2N2) master donor strain background. The cold-adapted, live monovalent A/17/mallard/Netherlands/00/95 (H7N3) influenza vaccine will be tested in this study. The virus strain in this vaccine was constructed in the laboratories of IEM by classical reassortment of

A/mallard/Netherlands/12/00(H7N3) virus with a cold-adapted temperature sensitive A/Leningrad/134/17/57(H2N2) virus master strain in developing chicken embryos. The 6:2 genomic reassortant was characterized for acceptable phenotype and genotype [presence of cold-adapted, temperature sensitive, hemagglutinin (HA), and neuraminidase (NA) markers], with genetic sequencing used to confirm the molecular basis of the attenuated phenotype. The developed vaccine strain was transferred to Microgen to produce a bulk lot of vaccine for use in preclinical immunogenicity and challenge studies and for use in clinical trials. Additional information on the development and preclinical testing of this candidate vaccine may be found in the Confidential Investigator's Brochure.

The World Health Organization (WHO) acknowledged advantages of LAIV use compared to inactivated vaccine in case of pandemic events. The main goal stated by WHO in the Global Influenza Preparedness Plan was to establish a collection of prepandemic influenza vaccines which could be used as a basis for vaccine manufacturing in a pandemic situation.[5]

In September of 2003 at an international workshop on pandemic influenza vaccines hosted by the United States National Institutes of Health, a clinical trial plan for pandemic influenza vaccines was developed and levels of priority for evaluation of different influenza candidate vaccine strains were proposed.[6] Influenza H1, H2 and H3 virus subtypes were evaluated as belonging to the "highest priority" level, and influenza viruses of H5, H6, H7 (such as that proposed in this trial), and H9 subtypes were assigned to a "high priority" level. In addition to this, the importance of our proposed trial is supported by WHO's decision to include LAIVs in the WHO global action plan to increase vaccine supply for influenza vaccines.[7]

Prior clinical trials have demonstrated that the Russian monovalent LAIV "Ultragrivac" (manufacturered by Microgen) based on 7:1 reassortant of A/duck/Potsdam/1402-6/86 (H5N2) apathogenic avian influenza virus with A/Leningrad/134/17/57 (H2N2) master donor virus was well-tolerated and immunogenic in healthy adult volunteers.[8] Results showed that the vaccine was minimally reactogenic, safe and genetically stable. The seroconversion rate after two doses of the vaccine, defined by a four-fold rise in HAI titre, was approximately 50%. Based on these promising results, WHO stated that avian-human reassortants with the Leningrad 17 strain deserve additional study.[9]

Also of importance is that the Russian LAIV, based on a 6:2 reassortant of A/PR/8/34 (H1N1) with the master donor strain, has been shown in studies in mice to induce not only humoral responses, but also cellular immune responses at the initial site of infection, including $CD4^+$, $CD8^+$ and $CD19^+$ responses.[10]

A number of LAIVs, derived from cold-adapted A/AnnArbor/6/60 *ca* (H2N2) Master Donor Virus (MDV-A; Medimmune, Gaithersburg, MD, USA) and avian influenza virus strains containing H5, H6, H7, and H9, have been produced and tested in healthy adult volunteers in phase I trials in the United States. All candidates were well-tolerated.[11,12,13,14] However, these LAIVs were highly restricted in replication in healthy seronegative adults and induced variable immune responses. The seroconversion rates after two doses of the vaccine, defined by a four-fold rise in HAI titre, were 92% for Medimmune's H7N3 candidate, 62% for the H9N2 candidate, 0-10% for two different H5N1 candidates (one of which was produced at different virus doses), and 5% for the H6N1 candidate. Although HAI seroconversion rates

were moderate for some of the candidate vaccines, mean HAI titres were consistently low. Interestingly, serum IgA responses appeared to be most consistent measure of an immune response in these studies. In summary, live attenuated influenza vaccines based on H7N3, H9N2, H6N1 and H5N1 avian viruses and the American A/Ann Arbor/6/60ca (H2N2) master donor virus were also well tolerated but displayed varying degrees of immunogenicity.

2.2 Dose Rationale

According to the WHO data influenza A/H7N3 virus is a potentially pandemic subtype to which the human population is immunologically “naïve”, i.e., has no preexisting antibodies to this agent and lacks immune memory against the said virus. Results obtained by the FSUC “SIC Microgen” MOHSD RF within clinical evaluation of pre-pandemic (H5N1) and pandemic (H1N1) live influenza vaccines showed inability of single-dose vaccination to generate adequate protective immune responses in “naïve” humans. Hence, in this study volunteers will be immunized with two doses given intranasally 28 days apart (by delivery of 0.25 ml into each nasal passage).

2.3 Potential Risks and Benefits

2.3.1 Potential Risks

2.3.1.1 To Participant Well-Being

Administration of study vaccine or placebo may cause the subject only mild discomfort or a tickling sensation in the nasal passages. The study vaccine to be used for this trial is produced by the same classical reassortant method as is seasonal trivalent LAIV currently approved for use in Russia; therefore, side effects may be similar. These may include, minimally, mild flu-like symptoms. The avian influenza virus strain [A/mallard/Netherlands/12/00(H7N3)] utilized to construct the study vaccine virus, is a low-pathogenicity strain. Moreover, it has been reassorted with the same cold-adapted master strain [A/Leningrad/134/17/57(H2N2)] utilized to construct seasonal LAIV. All attenuation mutations have been confirmed by molecular sequencing and attenuated phenotype was confirmed through preclinical testing in animals (See Confidential Investigator’s Brochure). Thus, no increased pathogenicity is expected in humans. The World Health Organization’s Quality, Safety and Standards department considers the likelihood of direct harm from reassortants derived from H5 or H7 strains in which the multiple basic amino acid cleavage site has been removed to be “very remote.”[15] However, serious or allergic reactions may be possible. These might include symptoms of pneumonia in the unlikely event that the attenuated phenotype is not sufficiently preserved when administered to humans. Study subjects will be observed closely by qualified clinicians, and emergency care will be immediately available to subjects in the isolation unit throughout their stay in the unit. If additional urgent care or resources are needed, the subject will be transported to a local hospital. This hospital will be identified by the investigator prior to study initiation, and appropriate contingencies for isolation of the subject will be made. The study will provide this care to the subject at no cost to the subject.

It is theoretically possible that vaccine virus that is replicating in a subject's upper respiratory tract may be shed and transmitted to those around the subject. If a subject decides to leave the isolation unit prematurely, the subject will be counseled on the theoretical risks to their household contacts and the community. The subject will also be told that local public health authorities might be notified of the subject's premature exiting from the isolation unit and that such authorities might quarantine the subject. A subject who is shedding virus is also potentially infectious to placebo recipients in the isolation facility. All subjects will be monitored closely for infection. (Study staff will be closely monitored outside of the study protocol and use of antivirals prophylactically during the dosing stages may be part of their terms of employment.)

Besides administration of study vaccine or placebo, collection of biological specimens (nasal swab, nasal wick, and blood specimens) may cause some discomfort to subjects. Collection of nasal swab and nasal wick specimens may cause mild discomfort or a tickling sensation in the nasal passages during the collection procedures. Venipuncture is sometimes associated with discomfort or pain, redness, swelling, and/or local hardness at the puncture site.

2.3.1.2 To Participant Privacy

Personal identifiers, including name, birth date, sex, location/address of residence will be collected and recorded on some study data collection forms. As a result, a potential risk for "loss of confidentiality" exists. To avoid this risk, subjects will be assigned a unique study participant number that will be used to identify the participant and link, using a master linking document, an individual to his/her study data and/or biological specimens. Whenever feasible, use of identifiers will be avoided and the unique study participant numbers will be used instead. Case report forms (CRF) to be sent to the Sponsor or PVS (or their representatives) for data management will contain only unique study participant numbers to identify the participant. Paper-based records will be kept in a secure location and only be accessible to authorized personnel involved in the study. Computer-based files will only be made available to personnel involved in the study through the use of access privileges and passwords. Individual participants will not be identified in any study related reports. Study staff will treat all study data, including subject identifiers and laboratory testing results, as confidential and not to be shared with anyone not authorized to view such data.

Biological specimens will be identified by study participant number; no personal identifiers will be utilized on any biological specimen. Likewise, laboratory reports will utilize only study participant numbers. Biological specimens will be stored in the laboratories of the Research Institute of Influenza (RII) and IEM, as appropriate to where that biological specimen will be tested using assays specified in this study protocol.

Biological specimens will be utilized only for testing in assays specified in this study protocol and approved by overseeing ethical review committees. However, biological specimens may become an extremely valuable resource for future development of improved influenza vaccines against avian and/or pandemic influenza. If written informed consent for long-term storage of any subject's specimens is obtained, those specimens will not be used for

unrelated, future studies without appropriate ethical review and approval and consent from study subjects, if deemed necessary by the overseeing ethical review committees.

2.3.2 Known Potential Benefits

By participating in this study, subjects will be screened by qualified clinicians for health status and through laboratory testing. This screening will be free of cost to the subject and may provide important information to the subject on the subject’s health status.

By participating in this study, subjects will contribute information on the safety and immunogenicity of this new vaccine against an avian influenza virus of pandemic potential. Development of new influenza vaccines, such as this LAIV, that are easier and faster to produce, and potentially less expensive, would be a great contribution to global public health.

There are no other known potential benefits to the subject for volunteering for participation in this trial. However, given the recent history of good safety profiles of LAIVs containing avian reassortants, the risk-benefit ratio is considered favorable, especially considering that all subjects will be under constant care of qualified medical personnel.

3 Objectives

3.1 Study Objectives

3.1.1 Primary Objective

To describe the safety profile of two intranasal doses of LAIV A/17/mallard/Netherlands/00/95 (H7N3) in healthy adults.

3.1.2 Secondary and Exploratory Objectives:

To describe the post-vaccination serologic immune responses to influenza vaccine.

To describe the post-vaccination shedding of vaccine virus and genetic stability of any shed virus.

Exploratory: To describe the post-vaccination cellular immune responses to influenza vaccine.

3.2 Study Outcome Measures

3.2.1 Primary Outcome Measures

The safety profile will be parameterized as the proportion of subjects experiencing adverse events (AEs) of the following four categories:

- Immediate reactions occurring within two hours of administration of any dose, measured as observed by study staff or reported by the subject to study staff.
- Adverse events commonly associated with intranasal vaccination (solicited local and systemic reactions) occurring greater than two hours after administration of any dose of study vaccine or placebo through 7 days following any dose, measured as observed by study staff or reported by the subject to study staff.
- All other adverse events (including unsolicited events) occurring during the 7 days following any dose, measured as observed by study staff or reported by the subject to study staff. This includes abnormal laboratory findings from blood and urine specimens collected on Days 7 and 35.
- All serious adverse events (SAEs) occurring within 4 weeks of receipt of any dose, as observed by study staff, reported by the subject to study staff, or noted by the subject on a diary card. This includes abnormal laboratory findings from blood and urine specimens collected on Days 28 (pre-vaccination) and 56.

3.2.2 Secondary Outcome Measures

Immune responses will be parameterized as the proportion of subjects with at least a four-fold rise after each dose from baseline or as the mean titer after each dose in any of the following:

- Serum hemagglutination inhibition (HAI) antibodies
- Serum neutralizing antibodies
- Serum immunoglobulin class A (IgA) or immunoglobulin class G (IgG) antibodies measured by enzyme-linked immunoassay (EIA)
- Mucosal IgA antibodies in nasal wick specimen

Virus shedding will be parameterized as the proportion of subjects shedding virus [detected by real-time reverse transcriptase polymerase chain reaction (rRTPCR) in nasal or conjunctival swabs or by isolation in chicken embryos] at any time-point. Shedding data will be reported for every subject at every measured time-point. Molecular characterization of any isolated shed virus will be reported with sequence details of any loss of attenuation mutations.

3.2.3 Exploratory Outcome Measures

Cellular immune responses (cytokine and T-cell) will also be measured using isolated peripheral blood mononuclear cells (PBMCs) tested by flow cytometry and/or enzyme-linked immunosorbent spot (ELISPOT) assay. These analyses will be exploratory and descriptive only.

4 Study Design

This is a phase I, double-blind, individually-randomized (3:1, vaccine:placebo), controlled trial with two groups, LAIV H7N3 and matched placebo. Healthy male and female adults 18 through 49 years of age will be invited to participate. For feasibility reasons and in order for an independent Safety Monitoring Committee (SMC) to review safety data in a small group of subjects initially, the total cohort of 40 subjects will be enrolled in two sub-cohorts: one cohort of 12 subjects, randomized at 3:1 (9 vaccine and 3 placebo), followed two weeks later by a second cohort of 28 subjects randomized at 3:1 (21 vaccine and 7 placebo). After all 12 volunteers of the first sub-cohort have been observed for the first isolation period (Day 1 to Day 7), an interim safety review will be performed by the SMC. The SMC will review all AEs, including clinical laboratory evaluations (pre- and post-vaccination) and shedding data, for all subjects and will advise if the volunteers of the first sub-cohort may receive dose two of study vaccine or placebo and if the additional 28 volunteers of the second sub-cohort may be enrolled into the study. As for the first sub-cohort, the SMC will also review all safety data for the second sub-cohort and for the entire participant population of the trial. For each sub-cohort, the procedures and timelines are here summarized.

On the day of first screening, about 7 days (between 4 and 14 days) prior to administration of dose one of study vaccine or placebo, subjects will be screened for eligibility through medical history review, physical examination, testing for serologic evidence of chronic viral infection [human immunodeficiency virus (HIV), hepatitis B virus (HBV) or hepatitis C virus (HCV), with proper pre- and post-test counseling], routine biochemical and hematological blood tests and urinalysis by dipstick.

Subject screening for eligibility will continue and be completed on the second screening day (S2). This second screening day will occur the same day as scheduled admission to the isolation unit and administration of study vaccine or placebo (Day 0). Women will undergo pregnancy tests using urine samples. All subjects will undergo an ear, nose and throat (ENT) examination. Fully eligible subjects will be admitted to the isolation unit. At that time, nasal swab, nasal wick, and blood specimens will be collected for virologic and immunological testing prior to administration of study vaccine or placebo. Blood and urine specimens will be again collected for routine biochemical and hematological blood tests and urinalysis by dipstick; these results will serve to define baseline status for subject prior to receipt of study vaccine or placebo but will not be used for screening purposes. Subjects will be unaware of which allocation, LAIV H7N3 or matched placebo, is received; study vaccine and placebo will be masked. Subjects will be carefully monitored for adverse reactions while in the isolation unit.

All subjects will remain in the isolation unit for at least 7 days after receipt of study vaccine or placebo. Nasal swabs will be collected daily while subjects are in isolation to test for presence of influenza virus shed in the nasal passage. Any subject exhibiting conjunctivitis will also have a conjunctival swab collected on the day of appearance of the sign. Any subject exhibiting influenza A virus shedding, as determined by real-time RT-PCR positivity on a nasal swab specimen, in the 2 days prior to each planned discharge day after each dose (Days 6 or 7 or Days 34 or 35) will be kept in the isolation until PCR-diagnosis results

confirm that no influenza virus is present in a tested clinical specimen for at least two consecutive days.
Any subject still exhibiting evidence of influenza virus shedding in a nasal swab on Days 6 or 7 or Days 34 or 35 post-administration with each dose might be placed on influenza antiviral (oseltamivir) treatment at the standard dose for treatment of 75 milligrams (mg) twice a day for a course of 5 days.

After discharge from the isolation unit, subjects will complete diary cards for AEs and use of concomitant medications. Subjects will return to the isolation unit at four weeks (Day 28) after administration of dose one of study vaccine or placebo. At that time, similar procedures will be used for admittance to the isolation unit, for receipt of dose two of study vaccine or placebo and for isolation and follow-up, with the additional procedure of review of interim histories (and diary cards) since first discharge after dose one.

After second discharge from the isolation unit, subjects will again complete diary cards for AEs and use of concomitant medications. Subjects will then return to the study center at four weeks (Day 56) after administration of dose two of study vaccine or placebo for their final study visit. Interim histories (and diary cards) will again be reviewed and final blood and nasal wick specimens will be collected. Women will also undergo a final pregnancy screen. Subjects will complete the study at this time.

For assessment of safety, subjects will be observed for two hours after each administration of study vaccine or placebo. Twice daily (early morning and late afternoon) examination will be also used to assess reactions for 7 days after each administration of study vaccine or placebo. ENT examination will also occur once per day on Days 7, 28, 35 and 56. Subjects will complete diary cards for unsolicited AEs from the day of each discharge until return to the isolation unit for dose two (at Day 28) or until return to the study center for the final study visit at four weeks post dose two (at Day 56). To assess safety, blood and urine specimens will also be collected on days 7, 28 (prior to administration of dose two of study vaccine or placebo), 35 and 56, for testing by routine biochemical and hematological blood tests and by urinalysis by dipstick.

For the evaluation of mucosal IgA antibody, nasal wick specimens will be collected on Day 0 (prior to administration of dose one of study vaccine or placebo), on Day 28 (prior to administration of dose two of study vaccine or placebo) and on Day 56. For the evaluation of serum antibodies (by HAI, microneutralization and IgA and IgG EIA), serum specimens will be collected on Day 0 (prior to administration of dose one of study vaccine or placebo), on Day 28 (prior to administration of dose two of study vaccine or placebo) and on Day 56. To study virus infectivity (by isolation in chicken embryos) and stability (by molecular sequencing of any isolated virus), nasal swab specimens will be taken on Days 1, 2, 3, 5, 7, 29, and 31. To assess priming and stimulation of cytotoxic T lymphocytes and other cytokine indicators, whole blood for isolation of PBMCs will be collected on Days 0 (prior to administration of dose one of study vaccine or placebo), on Day 28 (prior to administration of dose two of study vaccine or placebo) and on Day 56.

5 Study Enrollment and Withdrawal

Forty health male and female adult volunteers will be included in the full vaccine study. It is expected that up to 50 persons may need to be screened in order to confirm eligibility for 40 subjects.

5.1 Subject Inclusion Criteria

Questions about eligibility must be addressed prior to enrollment. The following criteria must be met before a subject may be enrolled for participation:

- Legal male or female adult 18 through 49 years of age at the enrollment visit.
- Literate and willing to provide written informed consent.
- Free of obvious health problems, as established by the medical history and screening evaluations, including physical examination.
- Capable and willing to complete diary cards and willing to return for all follow-up visits
- Willing to comply with the rules of the isolation unit (including willing and able to take oseltamivir influenza antiviral medication, should that be recommended by a study physician).
- For females, willing to take reliable birth control measures throughout the entire period of participation in the study.

5.2 Subject Exclusion Criteria

Subjects with any of the following conditions will be excluded from participation:

- Participation in another clinical trial involving any therapy within the previous three months or planned enrollment in such a trial during the period of this study.
- Receipt of any non-study vaccine within four weeks prior to enrollment or refusal to postpone receipt of such vaccines until four weeks after study completion.
- Practice of nasal irrigation on a regular basis within the past six months or has engaged in nasal irrigation within two weeks prior to enrollment.
- Recent history of frequent nose bleeds (>5 within the past year).
- Clinically relevant abnormal paranasal anatomy.
- Recent history (within the past month) of rhino or sinus surgery, or surgery for any traumatic injury of the nose.
- Current or recent (within two weeks of enrollment) acute respiratory illness with or without fever.
- Other acute illness at the time of study enrollment.

- Receipt of immune globulin or other blood products within three months prior to study enrollment or planned receipt of such products during the period of subject participation in the study.
- Chronic administration (defined as more than 14 consecutively-prescribed days) of immunosuppressants or other immune-modulating therapy within six months prior to study enrollment. (For corticosteroids, this means prednisone or equivalent, ≥0.5 mg per kg per day; topical steroids are allowed, exclusive of nasal.)
- Participation in any previous trial of any H5 or H7 containing influenza vaccine.
- History of asthma.
- Hypersensitivity after previous administration of any influenza vaccine.
- History of wheezing after past receipt of any live influenza vaccine.
- Other AE following immunization, at least possibly related to previous receipt of any influenza vaccine.
- Suspected or known hypersensitivity to any of the study vaccine components, including chicken or egg protein.
- Seasonal (autumnal) hypersensitivity to the natural environment.
- Acute or chronic clinically significant pulmonary, cardiovascular, hepatic, metabolic, neurologic, psychiatric or renal functional abnormality, as determined by medical history, physical examination or clinical laboratory screening tests, which in the opinion of the investigator, might interfere with the study objectives. Subjects with physical examination findings or clinical laboratory screening results which would be graded 2 or higher on the AE severity grading scale (see Attachments) will be excluded from entry into the study and will be excluded from receipt of dose two of study vaccine or placebo.
- History of leukemia or any other blood or solid organ cancer.
- History of thrombocytopenic purpura or known bleeding disorder.
- History of seizures.
- Known or suspected immunosuppressive or immunodeficient condition of any kind, including HIV infection.
- Known chronic HBV or HCV infection.
- Known tuberculosis infection or evidence of previous tuberculosis exposure.
- History of chronic alcohol abuse and/or illegal drug use.
- Claustrophobia or sociophobia.
- Pregnancy or lactation. (A negative pregnancy test will be required before administration of study vaccine or placebo for all women of childbearing potential.)
- Any condition that, in the opinion of the investigator, would increase the health risk to the subject if he/she participates in the study or would interfere with the evaluation of the study objectives.

5.3 Treatment Assignment Procedures

5.3.1 Randomization Procedures

This is a phase I, double-blind, individually-randomized (3:1, study vaccine:placebo), controlled trial with two groups, LAIV H7N3 and placebo. Forty healthy adults 18 through 49 years of age will be included in the trial. Study vaccine or placebo will be allocated to codes through the use of a computerized randomization scheme generator. A detailed *Randomization* standard operating procedure (SOP) will be developed prior to study initiation. Regardless of exact plan, an allocation code will be randomly assigned to each subject using a method which maintains the 3:1 ratio of study vaccine to placebo in each study sub-cohort.

5.3.2 Masking Procedures

Study vaccine and placebo will have similar formulations (LAIV H7N3 is a lyophilized formulation which must be reconstituted with sterile water for injections and placebo is the same lyophilized material without vaccine virus). However, because study vaccine and placebo will be clearly labeled as such, an unblinded study clinician will be assigned to prepare all study vaccine and placebo preparations. This study clinician will not administer the preparations and will not reveal to either other study staff or to the subject which preparation was given. Preparation of study vaccine or placebo and filling of spray dosing devices will be done by the unblinded clinician behind a screen. Filled devices will be carefully handed to the blinded study clinician administering the preparation. Preparation of study vaccine and placebo and filling of spray dosing devices will be closely monitored by an independent monitor from PATH Vaccine Solutions (PVS) and a Sponsor representative. Adherence to randomization will also be closely monitored to confirm that the randomization scheme is not broken. Study vaccine or placebo given to each individual subject will be documented on the CRF with the appropriate allocation code. Full details will be specified in a *Masking* SOP prior to trial initiation.

All blinded study staff will remain blinded throughout the trial. If any subject develops an SAE possibly related to receipt of study treatment, treatment allocation to the subject may be communicated to the investigator only if that information is deemed necessary to properly treat the subject for the SAE. In such cases, PVS must be consulted prior to any unblinding.

In order to maintain isolation unit staff blinding throughout the study, one designated investigator will receive all daily results of rRTPCR testing. This investigator will maintain these results in a secure location and will not share results with other investigators or study staff. Likewise, laboratory personnel conducting the rRTPCR testing will not share the results with others not designated to receive these results. Only in the event that a subject prematurely leaves the isolation unit for any reason may any subject’s influenza A positivity be revealed. The designated person holding these results will not participate in study evaluations of safety. This designated person will be consulted on Day 7 and Day 35 in order to confirm that no subject has influenza A positivity in a nasal swab specimen collected in either of the final two days of planned stay in the isolation unit after each dose.

5.4 Subject Withdrawal from the Trial

5.4.1 Reasons for Withdrawal (Premature Termination)

After enrollment, a subject might be withdrawn from participation for several reasons:

- The investigator requests withdrawal of participation of the subject because the subject is not eligible or is no longer eligible, because the subject has developed an AE requiring withdrawal, or because the subject is non-compliant;
- A subject voluntarily withdrawals participation at his/her own request;
- A subject is lost to follow-up; or
- The trial is terminated prematurely by the Sponsor or PVS.

5.4.2 Withdrawals at Specific Time-Points

5.4.2.1 Withdrawal of a Subject during Screening

If after signing by the volunteer of the informed consent form (ICF) for screening (ICF A), the investigator ascertains during the screening period (from S1 to S2/Day 0) that the subject does not meet the inclusion/exclusion criteria, the subject should be assigned a study participant number but the CRF pages need not be completed. Only the reason for subject exclusion should be noted on an enrollment log.

5.4.2.2 Withdrawal of a Subject as a Result of Erroneous Inclusion

If a subject who does not meet the inclusion/exclusion criteria is inadvertently included in the trial, the investigator must terminate the subject’s participation in the trial and the Sponsor and PVS should be informed. If the subject was randomized and was administered study vaccine or placebo, the subject should be maintained in the isolation unit for the specified period, if possible. However, the subject’s data will be excluded from the analysis.

5.4.2.3 Withdrawal of a Subject as a Result of Appearance of Exclusion Criteria After Administration of Study Vaccine or Placebo

If a subject develops a concomitant illness that is an exclusion criterion or, for any reason, requires treatment with unauthorized products, the subject may be withdrawn from the study. See Handling of Withdrawals and Concomitant and Unauthorized Products. In all such cases, the Sponsor and PVS should be consulted immediately.

5.4.2.4 Withdrawal of a Subject for Violation of the Protocol

A subject will be considered as largely not in compliance with the requirements of the protocol if any of the following conditions apply:

- Violation of the rules of the isolation unit;
- Substantially incorrect or incomplete completion of the self-observation diary;

- Skipping a schedule visit;
- Non-compliance at a scheduled visit (except cases of exclusion of volunteers from the trial) of any planned procedures; or
- Failure to inform the investigator of the appearance of any AE or SAE in the subject.

5.4.2.5 Withdrawal of a Subject as a Result of Withdrawal of Informed Consent

If a subject wishes to withdraw consent for participation in the trial after the subject has been admitted to the isolation unit and has been administered dose one or dose two of study vaccine or placebo, the subject will be requested to complete evaluations for that day and to complete either Day 28 or Day 56 evaluations, as applicable. Besides, to ensure safety any subject exhibiting influenza virus shedding in a nasal swab specimen will remain in the isolation unit for a more prolonged period until results confirming that no influenza virus is detected in a nasal swab for at least two consecutive days are obtained.

If possible, the subject should indicate the reasons for the termination of participation. The subject will be informed that the public health authorities will be notified of the subject's premature discharge from the isolation unit, as well as of the results of rRTPCR testing of the subject's nasal swab specimens for detection of viral shedding. The subject will be advised to remain at home and avoid close contact with other persons until 7 days post-administration of study vaccine or placebo have passed.

If a subject wishes to withdrawal consent for participation in the trial after the subject has been discharged from the isolation unit, the subject will be requested to return the self-observation diary card and complete either Day 28 or Day 56 evaluations, as applicable. If possible, the subject should indicate the reasons for the termination of participation.

5.4.2.6 Withdrawal of a Subject as a Result of Occurrence of a Serious Adverse Event

Follow-up visits will be carried out for all subjects who are withdrawn due to occurrence of an AE or in connection with a change in any other safety indicator (vital sign or clinical laboratory result).

If any subject develops an SAE leading to premature withdrawal, the event will be fully followed-up to resolution of the problem, to acknowledgement of the diagnosis of the SAE or to change in status of the acute SAE as chronic or stable or as long as such a change is justified from a clinical point of view. However, for premature withdrawal in connection with the emergence an SAE that is considered to be clinically relatively favorable (for example, the diagnosis is known, and it is expected to be resolved completely within a week), intermittent follow-up may be accepted as long as the plan for follow-up of the event is fully described in the notes section of the subject's CRF.

5.4.3 Handling of Withdrawals

In case of premature withdrawal for any reason, the investigator should exert his/her best effort to:

- Conduct an interview to determine if the subject has had any reaction or AE (serious or non-serious) during the period one month after any study vaccination that has led to the withdrawal. Where possible, the investigator should visibly or physically assess any reported adverse reaction or AE.
- Attempt to complete all scheduled procedures and evaluations planned for a) Day 28 (exclusive of administration of study vaccine or placebo) if premature withdrawal occurs prior to Day 28 but after administration of dose one of study vaccine or placebo or b) Day 56 if premature withdrawal occurs prior to Day 56 but after administration of dose two of study vaccine or placebo.
- Document the reason for premature withdrawal on the CRF.

The trial Sponsor and PVS must be informed within 24 hours about all instances of the premature termination of a volunteer's participation in the trial. Any withdrawal that occurs while a subject is scheduled to be in the isolation unit requires immediate notification to the Sponsor and PVS. While participation in the trial is voluntary and subjects are free to withdraw consent at any time, subjects will be told that local public health authorities might be notified of any subject's premature exiting from the isolation unit. An SOP for the isolation unit will detail procedures to be followed for any withdrawal that occurs while a subject is scheduled to be in the isolation unit.

Withdrawal of a subject *by the investigator* may occur only if the subject is identified to have one of the listed exclusion criteria or if continued participation of the subject threatens the subject's well-being or the integrity of the study (or the well-being of other subjects in the isolation unit). After administration of dose one (or dose two) of study vaccine, a subject who is scheduled to be in the isolation unit may be withdrawn *by the investigator* only if the subject develops a reaction to study vaccine which the investigator believes threatens the subject's well-being; the withdrawn subject must be treated or transferred to a treatment facility.

5.5 Termination of the Trial

5.5.1 Termination According to the Protocol

Termination of the trial according to the protocol is the date of the last visit of the last subject participating in the trial, according to the trial scheme. Of note, each sub-cohort will have a different termination date for that sub-cohort.

5.5.2 Suspension and/or Premature Termination of the Trial

The trial might be suspended at any time by the Sponsor, PVS, the Russian Ministry of Health, the SMC, any ethical review committee overseeing this study, or by the investigator for any safety concern. This includes, for example, and without limitation, an SAE resulting in death, an unexpectedly high number of persons shedding virus after receipt of any dose, an unusually high rate of SAEs, or an unexpectedly early onset of substantial seasonal influenza virus circulation in the surrounding community. PVS may suspend the study in the event that study conduct is found to be below Good Clinical Practice (GCP) standards.

In the event of the appearance of new data that indicate an increased level of risk to participating subjects, the clinical trial will be suspended until the Sponsor, PVS, the Russian Ministry of Health, the SMC, and all ethical review committees have reviewed relevant data and agreed that the trial may proceed.

6 Study Products

6.1 Study Product Descriptions

6.1.1 Study Vaccine

Study vaccine for this trial is a cold-adapted, live attenuated monovalent A/17/mallard/Netherlands/00/95(H7N3) influenza vaccine. The virus strain in this vaccine was constructed in the laboratories of IEM by classical reassortment of A/mallard/Netherlands/12/00(H7N3) virus with a cold-adapted temperature sensitive A/Leningrad/134/17/57(H2N2) virus master donor strain in developing chicken embryos. The 6:2 genomic reassortant was characterized for acceptable phenotype and genotype [presence of cold-adapted, temperature sensitive, HA, and NA markers], with genetic sequencing used to confirm the molecular basis of the attenuated phenotype. The developed vaccine strain was transferred to Microgen, to produce a bulk lot of vaccine for use in preclinical immunogenicity and challenge studies and for use in clinical trials. Manufactured vaccine was obtained from virus-containing allantoic fluid of chicken embryos. Microgen has transferred the manufactured vaccine back to IEM where it has been securely stored.

LAIV H7N3 is a lyophilizate (a light yellow-colored amorphous mass) of live attenuated influenza virus for the preparation of a solution for intranasal introduction. The active component is the vaccine strain A/17/Mallard/Netherlands/00/95(H7N3) virus obtained from the allantoic fluid of chicken embryos. LAIV H7N3 is supplied lyophilized in single-dose ampoules [specific activity of the vaccine is not less than 7.5 log EID_{50}/0.5 ml dose]. The lyophilizate also contains the following stabilizers: sucrose, lactose, glycine, sodium glutamate, tris (hydroxymethyl) amino-methane, sodium chloride, and gelatin.

6.1.2 Placebo

Placebo will be manufactured to have a similar formulation and presentation as study vaccine. The production process starts with allantoic fluid of chicken embryos that have not been inoculated with any influenza virus. A lyophilizate also containing the same concentrations of stabilizers as LAIV is then made and filled in ampoules. Microgen has transferred the manufactured placebo back to IEM where it has been securely stored.

6.1.3 Acquisition

The investigator or study coordinator, if applicable, will be personally responsible for vaccine receipt and management or will designate a person who will be responsible for these activities. PVS and IEM will determine with the investigator or the person in charge, the

date(s) and time(s) of delivery of vaccine to the study site. Study vaccine and placebo, manufactured by Microgen, will be supplied to the study site by IEM.

The person in charge of vaccine receipt will check that the cold chain was maintained during shipment: continuous verification of temperature and/or cold chain monitoring card. In case of any problem, he/she must alert PVS and IEM and Microgen immediately.

The acknowledgments of receipt of study vaccine and placebo will be dated and signed by the person in charge of vaccine at RII. Copies will be kept for archiving at the investigator's site and copies will be returned to PVS (for study vaccine and placebo) and IEM.

6.1.4 Packaging and Labeling

Study vaccine and placebo will be packed identically, but each package will clearly identify each study vaccine and placebo as such.

6.1.5 Storage and Stability

LAIV H7N3 and placebo should be stored at a temperature between +2 degrees Celsius (°C) to +8°C (in a refrigerator). Storage temperature should be monitored daily and documented on an appropriate form. Back-up power or storage should be available in case of primary power failure. Study vaccine and placebo should never be frozen. The study vaccine or placebo should not be used if the ampoule is broken, the label is illegible or the physical properties (color and transparency) are altered.

In case of accidental disruption of the cold chain, the products should not be administered and the investigator or the responsible person should contact the Sponsor and PVS to receive further instructions. In such cases, the investigator must receive written consent of the PVS before any study vaccine or placebo may be used.

6.2 Dosage, Preparation and Administration of Study Products

6.2.1 Dosage

Dosage of study vaccine or placebo will be two doses given 28 days apart. Each dose will be a total of 0.5 ml of prepared study vaccine or placebo, with 0.25 ml delivered intranasally into each nasal passage using a spray dosing device.

6.2.2 Preparation and Administration

Directly before inoculation of study vaccine or placebo, the contents of an ampoule must be dissolved in 0.5 ml of water for injections for 3 minutes. The dissolved product is a pale yellow, slightly opalescent liquid. The dissolved product should be used within 30 minutes.

For preparation and intranasal administration, a spray dosing device with disposable applicator is used. The spray dosing device consists of a sterile syringe with scales of 40 and 100 units (GOST R ISO 10993, GOST 24861-91, GOST 25026-81), a sterile needle, and one adjustable end cap actuator-183.016 for the creation of a fine spray mist.

To prepare and administer study vaccine or placebo, the complete process is as follows:

- The needle will be placed on the syringe, and 0.5 ml of water for injections will be drawn (to the mark of 20 on the scale of 40 units or to the mark of 50 on the scale of 100 units).
- The ampoule of study vaccine or placebo will be opened, and the 0.5 ml of water added from the syringe.
- After 3 minutes for dissolution of study vaccine or placebo, 0.25 ml of the now-liquid preparation will be drawn into the syringe (to the mark of 10 on the scale of 40 units or the mark of 25 on the scale of 100 units).
- The needle will be removed and placed on a clean or sterile surface, and the spray actuator will be place tightly on the syringe.
- With the subject seated in a comfortable position with the head slightly tilted back, the tip of the spray actuator will be brought up to the subject’s **right** nostril and inserted 0.5 centimeters (cm) into the nasal passage, and the hub of the syringe strongly depressed to inject the product into the nasal passage. After the spray of vaccine into the nostril, the subject should remain in the seated position with the head slightly tilted back for 1 minute.
- After the first spray, the spray actuator will be removed and placed on a clean or sterile surface, and the needle will be replaced on the syringe. Another 0.25 ml of liquid preparation will be drawn into the syringe.
- The needle will once again be removed, and the spray actuator again placed on the syringe.
- With the subject seated in a comfortable position with the head slightly tilted back, the tip of the spray actuator will be brought up to the subject’s **left** nostril and inserted 0.5 cm into the nasal passage, and the hub of the syringe strongly depressed to inject the product into the nasal passage. Again, after the spray of vaccine into the nostril, the subject should remain in the seated position with the head slightly tilted back for 1 minute.

6.3 Accountability Procedures for Study Products

Study vaccine and placebo must be kept in a secure place. The investigator or the person in charge of study product management will maintain records of delivery of the products to the trial site, the inventory at the site, the dose(s) given to each participant, and the destruction or return of unused doses.

6.4 Concomitant and Unauthorized Products

6.4.1 Concomitant Medications/Treatment

If an accompanying pathology is other than a criterion for exclusion of a volunteer from participation in the trial, then the relevant treatment should be performed under its usual scheme. All concomitant medicines used should be allowed by the Protocol (see Section 6.4.2 “Unauthorized products”). Information on the concomitant products (trade name, dosage or change in dosing, indications, start date, and termination date) must be reflected

in the appropriate section in the CRF. All subsequent changes in concomitant therapy during the trial must also be reflected in the CRF.

Women included in the trial who are using oral contraceptives for pregnancy prevention should continue taking these products during the entire trial. Use of such products must also be documented in the CRF.

6.4.2 Unauthorized Products

Any concomitant MIBPs (medical immunobiological products) (including blood preparations), as well as other immune-modulating drugs are contraindicated.

The said products may be prescribed for life-saving purposes.

6.4.3 Use of Unauthorized Products or Products Not Stipulated by the Protocol

If during the trial a situation arises where there is an adverse reaction or AE requiring treatment and prescription of unauthorized products or products not stipulated by the protocol, then such products may be prescribed. However, PVS and Microgen must be informed within 24 hours. Information on the products (trade name, dosing or change in dosing, indications, start date, and termination date) must be recorded in the CRF. All subsequent changes in treatment during the trial must also be reflected in the CRF. Follow-up of the subject by the investigator will continue until the end of the subject's condition which was the cause for this violation of the protocol (in the case of early withdrawal from the trial) or to the end of the subject's schedule period of participation (to termination).

If any subject is required to take unauthorized products or products not stipulated by the protocol in order to treat an adverse reaction requiring withdrawal, then the investigator should follow procedures for Handling of Withdrawals.

7 Study Schedule

It is the responsibility of the investigator to strictly observe the intervals between visits/procedures. These intervals are precisely defined for each subject in accordance with the protocol and are reflected in the study schedule outlined below. Where not specified, time windows (for example, plus or minus a certain number of days) are not allowed.

7.1 Screening

Prior to inclusion into the vaccine study, subjects will be screened through multiple procedures, including laboratory testing, medical history interview and physical examination. Because screening procedures are required to assess eligibility, they will be performed under a screening ICF (ICF A) prior to the subject providing informed consent for study participation in vaccination and follow-up activities under a second vaccine trial informed consent form (ICF B) (see Section 14).

7.1.1 First Screening Visit (14 to 4 Days Prior to Trial Enrollment, Day S1)

After subjects are consented into the screening process (using ICF A), the following activities will occur:

1. The subject will be interviewed to collect baseline demographic data (name, date of birth, gender, race/ethnicity, etc.)
2. A study clinician will interview the subject to collect a detailed medical history. Study staff will review the information to confirm eligibility prior to conduct of further procedures.
3. A study clinician will perform a physical examination. Results will be reviewed by study staff and with the subject to confirm eligibility prior to conduct of further procedures.
4. Blood (serum and whole) specimens will be collected for testing in a comprehensive metabolic panel (CMP) and by complete blood count (CBC) with white blood cell (WBC) differential. Serum specimens will also be collected for testing for chronic viral infections. Specimen collection information must be documented in specimen collection logs.
5. A urine specimen will be collected for urinalysis testing via dipstick. Specimen collection information must be documented in specimen collection logs.
6. Study staff will instruct the subject on when to return for screening results and for official enrollment into the study and admittance to the isolation unit.

7.1.2 Second Screening Visit (Day S2) / Trial Enrollment Day (Day 0)

For subjects continuing with the screening process, the following activities will occur on Day 0:

1. Subjects must present to the study center by 08:00. Study staff will confirm subject identity.
2. Results of the CMP and CBC with differential will be reviewed by a study clinician and with the subject. Results of serologic testing for chronic viral infections will also be reviewed by a study clinician and with the subject. All results will be reviewed to confirm eligibility prior to conduct of further procedures.
3. For women only, a urine specimen will be collected for pregnancy testing.
4. Study staff will inquire about any new medical events since medical histories were recorded and confirm eligibility prior to conduct of further procedures.
5. A study clinician will perform a physical examination. Results will be reviewed by study staff and with the subject to confirm eligibility prior to conduct of further procedures.
6. A study otolaryngologist will perform an ear, nose, and throat (ENT) examination. Results will be reviewed by study staff and with the subject to confirm eligibility prior to conduct of further procedures.

7. If eligibility is confirmed, all information since the beginning of screening must be recorded on the study CRF.

7.2 Vaccination and Follow-up Periods

Only subjects consented and admitted into the isolation unit may undergo subsequent procedures.

7.2.1 Day of First Admission into the Isolation Unit (Day 0)

1. For eligible subjects, a study clinician will review the procedures for the vaccination study and admittance into the isolation unit. A vaccine study ICF (ICF B) should be completed at this time and the subject admitted into the isolation unit. Upon admittance into the isolation unit, a subject may proceed to undergo procedures related to administration of study vaccine or placebo and evaluation of vaccine safety and immunogenicity.
2. At least two hours prior to administration of study vaccine or placebo, a nasal swab specimen should be collected, followed one hour later by collection of a nasal wick specimen. Specimen collection information must be documented on the CRF and in specimen collection logs. Nasal wick specimens will be sent to IEM for mucosal anti-influenza IgA testing.
3. Prior to administration of study vaccine or placebo, a serum specimen will be collected for anti-influenza serologic assays. A whole blood specimen for isolating PBMCs for assessing cellular immune responses will also be collected at this time. Blood specimens will also be collected for testing a CMP and a CBC with differential. Specimen collection information must be documented on the CRF and in specimen collection logs. CMP and CBC with differential test results from specimens collected on Day 0 need not be obtained and reviewed prior to administration of study vaccine or placebo; these results will only served to define baseline status for subjects.
4. The subject will be administered one dose of study vaccine or placebo. Administration of study vaccine or placebo must occur at least one hour after collection of any nasal swab or nasal wick specimens from the subject.
5. The subject will be observed for two hours after administration in case of any immediate reactions. If the subject experiences an immediate adverse reaction, he/she will be treated and the event will be recorded on the CRF.
6. The subject will be observed throughout the day and any AEs that occur will be recorded in the appropriate section(s) of the CRF.
7. At least 6 hours after administration of study vaccine or placebo, a nasal swab specimen will be collected. Specimen collection information must be documented on the CRF and in specimen collection logs.

7.2.2 First Day after Administration of Dose One (Day 1)

1. In the morning between 09:00 and 10:00, a nasal swab specimen will be collected. Specimen collection information must be documented on the CRF and in specimen collection logs. Nasal swab specimens will be split for testing at RII and IEM. Nasal swab specimens should be tested at RII by rRTPCR for influenza A positivity within 30 hours of collection (results reported back to one designated investigator by 14:00 the next day). Nasal swab specimens sent to IEM will be used to isolate potentially shed virus in chicken embryos.
2. Once in the morning and once in the afternoon, study staff will interview the subject about any signs and symptoms the subject might be experiencing. Subjects will be encouraged to spontaneously report any concern or symptom they are experiencing. Any AEs that have occurred will be recorded in the appropriate section(s) of the CRF.
3. A study clinician will perform one scheduled physical examination and record the information on the CRF. Results will be reviewed by study staff and with the subject and any AEs that have occurred will be recorded in the appropriate section(s) of the CRF. Any AEs that have occurred will be recorded in the appropriate section(s) of the CRF and may necessitate additional exams throughout the day.

7.2.3 Second Day after Administration of Dose One (Day 2)

Planned procedures on this day are identical to those on Day 1.

7.2.4 Third Day after Administration of Dose One (Day 3)

Planned procedures on this day are identical to those on Day 1.

7.2.5 Fourth Day after Administration of Dose One (Day 4)

Planned procedures on this day are identical to those on Day 1, except that no nasal swab specimen will be sent to IEM for isolation of potentially shed virus in chicken embryos.

7.2.6 Fifth Day after Administration of Dose One (Day 5)

Planned procedures on this day are identical to those on Day 1.

7.2.7 Sixth Day after Administration of Dose One (Day 6)

Planned procedures on this day are identical to those on Day 1, except that no nasal swab specimen will be sent to IEM for isolation of potentially shed virus in chicken embryos.

7.2.8 Seventh Day after Administration of Dose One (Day 7)

1. In the morning between 09:00 and 10:00, a nasal swab specimen will be collected. Specimen collection information must be documented on the CRF and in specimen collection logs. Nasal swab specimens will be split for testing at RII and IEM. Nasal swab specimens should be tested at RII by rRTPCR for influenza A positivity on this

day with results reported back to one designated investigator by 16:00. Nasal swab specimens sent to IEM will be used to isolate potentially shed virus in chicken embryos.

2. Once in the morning and once in the afternoon, study staff will interview the subject about any signs and symptoms the subject might be experiencing. Subjects will be encouraged to spontaneously report any concern or symptom they are experiencing. Any AEs that have occurred will be recorded in the appropriate section(s) of the CRF.
3. A study clinician will perform one scheduled physical examination and record the information on the CRF. Results will be reviewed by study staff and with the subject and any AEs that have occurred will be recorded in the appropriate section(s) of the CRF. Any AEs that have occurred will be recorded in the appropriate section(s) of the CRF and may necessitate additional exams throughout the day.
4. A study otolaryngologist will perform an ENT examination. Results will be reviewed by study staff and with the subject and any AEs that have occurred will be recorded in the appropriate section(s) of the CRF.
5. Blood specimens will be collected for testing a CMP and a CBC with differential. Specimen collection information must be documented on the CRF and in specimen collection logs. Any out-of-normal-range result will be recorded as an AE and discussed with the investigator to determined continued eligibility for dose two of study vaccine or placebo.
6. A urine specimen will be collected for urinalysis testing via dipstick. Specimen collection information must be documented on the CRF and in specimen collection logs. Any out-of-normal-range result will be recorded as an AE and discussed with the investigator to determined continued eligibility for dose two of study vaccine or placebo.
7. Subjects will be given a diary card in which they will be asked to record any local and/or systemic reactions that might appear from Days 7 (after discharge from the isolation unit) to Day 28 (Day 28 being the scheduled day of readmission to the isolation unit for dose two of study vaccine or placebo) and to record any concomitant medications. Subjects will be instructed how to use the diary card on this day. All relevant explanations should be included in the diary card. The diary card will also have contact information for the investigators, should the subject have any questions.
8. The subject will be instructed that if the subject later experiences an AE requiring medical care, the subject should inform the investigator as soon as possible and seek medical care as appropriate. If the subject visits a health care provider, the subject should be sure to inform the health care providers of participation in this study and provide the provider with contact information for the investigator.
9. If the subject’s Day 6 and Day 7 nasal swab specimens are influenza A negative by rRTPCR, the subject will be discharged from the isolation unit and informed of the scheduled date of return to the unit.

7.2.9 Days after First Discharge from the Isolation Unit (Days 8 to 27)

1. Subjects will complete diary cards of any local or systemic reactions experienced.
2. The subject will be instructed that if he/she later experiences an AE requiring medical care, the subject should inform the investigator as soon as possible and seek medical care as appropriate. If the subject visits a health care provider, the subject should be sure to inform the health care providers of participation in this study and provide the provider with contact information for the investigator.

7.2.10 Day of Second Admission into the Isolation Unit (Day 28)

1. Subjects must present to the study center by 08:00. Study staff will confirm subject identity.
2. Study staff will review subject diary cards and interim histories with the subject and inquire about any new medical events since medical histories were last updated.
3. Blood specimens will be collected for testing a CMP and a CBC with differential. Specimen collection information must be documented on the CRF and in specimen collection logs.
4. A urine specimen will be collected for urinalysis testing via dipstick. Specimen collection information must be documented on the CRF and in specimen collection logs. For women only, the urine specimen will be collected for pregnancy testing. Any out-of-normal-range result will be recorded as an AE and discussed with the investigator to determined continued eligibility for dose two of study vaccine or placebo.
5. While blood specimens are being tested, a study clinician will perform a physical examination and record the information on the CRF. Results will be reviewed by study staff and with the subject and any AEs that have occurred will be recorded in the appropriate section(s) of the CRF
6. A study otolaryngologist will perform an ENT examination and record the information on the CRF. Results will be reviewed by study staff and with the subject and any AEs that have occurred will be recorded in the appropriate section(s) of the CRF.
7. Upon receipt of results of CMP and CBC with differential testing, results will be reviewed by study staff and with the subject. Any out-of-normal-range result will be recorded as an AE and discussed with the investigator to determined continued eligibility for dose two of study vaccine or placebo.
8. If continued eligibility is confirmed, a study clinician will again review the procedures for the vaccination study and admission into the isolation unit. Continued informed consent will be confirmed and the subject admitted into the isolation unit. Upon admission into the isolation unit, a subject may proceed to undergo procedures related to administration of study vaccine or placebo and evaluation of vaccine safety and immunogenicity.

If the subject is no longer eligible for receipt of dose two of study vaccine or placebo, the investigator should not admit the subject into the isolation unit but should conduct steps 9 and 10, below, prior to withdrawing the subject from the study.

9. At least two hours prior to administration of study vaccine or placebo, a nasal swab specimen should be collected, followed one hour later by collection of a nasal wick specimen. Specimen collection information must be documented on the CRF and in specimen collection logs. Nasal wick specimens will be sent to IEM for mucosal anti-influenza IgA testing.
10. Prior to administration of study vaccine or placebo, a serum specimen will be collected for anti-influenza serologic assays. A whole blood specimen for isolating PBMCs for assessing cellular immune responses will also be collected at this time.
11. The subject will be administered one dose of study vaccine or placebo. Administration of study vaccine or placebo must occur at least one hour after collection of any nasal swab or nasal wick specimens from the subject.
12. The subject will be observed for two hours after administration in case of any immediate reactions. If the subject experiences an immediate adverse reaction, he/she will be treated and the event will be recorded on the CRF.
13. The subject will be observed throughout the day and any AEs that occur will be recorded in the appropriate section(s) of the CRF.
14. At least 6 hours after administration of study vaccine or placebo, a nasal swab specimen will be collected. Specimen collection information must be documented on the CRF and in specimen collection logs.

Because many procedures must be completed on Day 28, an example of the schedule of procedures for Day 28 is provided in the Attachments.

7.2.11 First Day after Administration of Dose Two (Day 29)

1. In the morning between 09:00 and 10:00, a nasal swab specimen will be collected. Specimen collection information must be documented on the CRF and in specimen collection logs. Nasal swab specimens will be split for testing at RII and IEM. Nasal swab specimens should be tested at RII by rRTPCR for influenza A positivity within 30 hours of collection (results reported back to one designated investigator by 16:00 the day after collection). Nasal swab specimens sent to IEM will be used to isolate potentially shed virus in chicken embryos.
2. Once in the morning and once in the afternoon, study staff will interview the subject about any signs and symptoms the subject might be experiencing. Subjects will be encouraged to spontaneously report any concern or symptom they are experiencing. Any AEs that have occurred will be recorded in the appropriate section(s) of the CRF.
3. A study clinician will perform one scheduled physical examination and record the information on the CRF. Results will be reviewed by study staff and with the subject. Any AEs that have occurred will be recorded in the appropriate section(s) of the CRF and may necessitate additional exams throughout the day.

7.2.12 Second Day after Administration of Dose Two (Day 30)

Planned procedures on this day are identical to those on Day 29, except that no nasal swab specimen will be sent to IEM for isolation of potentially shed virus in chicken embryos.

7.2.13 Third Day after Administration of Dose Two (Day 31)

Planned procedures on this day are identical to those on Day 29.

7.2.14 Fourth Day after Administration of Dose Two (Day 32)

Planned procedures on this day are identical to those on Day 29, except that no nasal swab specimen will be sent to IEM for isolation of potentially shed virus in chicken embryos.

7.2.15 Fifth Day after Administration of Dose Two (Day 33)

Planned procedures on this day are identical to those on Day 29, except that no nasal swab specimen will be sent to IEM for isolation of potentially shed virus in chicken embryos.

7.2.16 Sixth Day after Administration of Dose Two (Day 34)

Planned procedures on this day are identical to those on Day 29, except that no nasal swab specimen will be sent to IEM for isolation of potentially shed virus in chicken embryos.

7.2.17 Seventh Day after Administration of Dose Two (Day 35)

1. In the morning between 09:00 and 10:00, a nasal swab specimen will be collected. Specimen collection information must be documented on the CRF and in specimen collection logs. Nasal swab specimens should be tested at RII by rRTPCR for influenza A positivity on this day with results reported back to one designated investigator by 16:00.
2. Once in the morning and once in the afternoon, study staff will interview the subject about any signs and symptoms the subject might be experiencing. Subjects will be encouraged to spontaneously report any concern or symptom they are experiencing. Any AEs that have occurred will be recorded in the appropriate section(s) of the CRF.
3. A study clinician will perform one scheduled physical examination and record the information on the CRF. Results will be reviewed by study staff and with the subject. Any AEs that have occurred will be recorded in the appropriate section(s) of the CRF and may necessitate additional exams throughout the day.
4. A study otolaryngologist will perform an ENT examination and record the information on the CRF. Results will be reviewed by study staff and with the subject and any AEs that have occurred will be recorded in the appropriate section(s) of the CRF.
5. Blood specimens will be collected for testing a CMP and a CBC with differential. Specimen collection information must be documented on the CRF and in specimen collection logs. Any out-of-normal-range result will be recorded as an AE.

6. A urine specimen will be collected for urinalysis testing via dipstick. Specimen collection information must be documented on the CRF and in specimen collection logs. Any out-of-normal-range result will be recorded as an AE.
7. Subjects will be given a second diary card in which they will be asked to record any local and/or systemic reactions that might appear from days 35 (after discharge from the isolation unit) to 56 and to record any concomitant medications. Subjects will be instructed how to use the second diary card on this day. All relevant explanations should be included in the second diary card. The second diary card will also have contact information for the investigators, should the subject have any questions.
8. The subject will be instructed that if the subject later experiences an AE requiring medical care, the subject should inform the investigator as soon as possible and seek medical care as appropriate. If the subject visits a health care provider, the subject should be sure to inform the health care providers of participation in this study and provide the provider with contact information for the investigator.
9. If the subject's Day 34 and Day 35 nasal swab specimens are influenza A negative by rRTPCR, the subject will be discharged from the isolation unit and informed of the scheduled date of return to the unit.

7.2.18 Days after Second Discharge from the Isolation Unit (Days 36 to 55)

1. Subjects will complete the second diary cards for any local or systemic reactions experienced.
2. The subject will be instructed that if he/she later experiences an AE requiring medical care, the subject should inform the investigator as soon as possible and seek medical care as appropriate. If the subject visits a health care provider, the subject should be sure to inform the health care providers of participation in this study and provide the provider with contact information for the investigator.

7.2.19 Day of Final Visit to the Study Center (Day 56)

1. Subjects will present to the study center. Study staff will confirm subject identity.
2. Study staff will review second subject diary cards and interim histories with the subject and inquire about any new medical events since medical histories were last updated.
3. Blood specimens will be collected for testing a CMP and a CBC with differential, for anti-influenza serologic assays, and for assessing cellular immune responses. Specimen collection information must be documented on the CRF and in specimen collection logs. Any out-of-normal-range result will be recorded as an AE. (If results of blood testing may be sent to the subject if the subject so requests.)
4. A urine specimen will be collected for urinalysis testing via dipstick. Specimen collection information must be documented in the CRF and in specimen collection logs. For women only, the urine specimen will be collected for pregnancy testing. Any out-of-normal-range result will be recorded as an AE.

5. A nasal wick specimen will be collected. Specimen collection information must be documented on the CRF and in specimen collection logs. Nasal wick specimens will be sent to IEM for mucosal anti-influenza IgA testing.
6. A study clinician will perform a physical examination and record the information on the CRF. Results will be reviewed by study staff and with the subject and any AEs that have occurred will be recorded in the appropriate section(s) of the CRF.
7. A study otolaryngologist will perform an ENT examination and record the information on the CRF. Results will be reviewed by study staff and with the subject and any AEs that have occurred will be recorded in the appropriate section(s) of the CRF.
8. Study staff will complete the final visit CRF and terminate the subject's participation in the study.

7.3 Unscheduled Visits

Subjects may present to the study center during operating hours for an unscheduled visit should they experience any AE, or if the subject's condition requires medical intervention. Data for any examinations performed on the subject at an unscheduled visit must be recorded in the CRF. If an unscheduled visit is performed, the procedures for the next following visit should not be made earlier than scheduled above.

8 Study Evaluations

8.1 Clinical Evaluations

8.1.1 Definition and Categorization of AEs

The primary objective of this study is to describe the safety profile of two intranasal doses of LAIV H7N3. AEs and SAEs are defined in Section 9. Grading of AEs is also described in Section 9. Included below are brief listings of specific clinical and laboratory safety measurements to be made. All clinical safety evaluations must be made by a qualified clinician (physician, physician assistant, or nurse practitioner) or will be self-reported by the subject.

Any clinical sign or symptom or laboratory finding at any time-point might be categorized as an SAE (see Section 9.2.3).

8.1.2 Specific Clinical Signs and Symptoms of Interest

All clinical signs and symptoms must be document. However, the following signs and symptoms, and their time and date of onset/occurrence and resolution, will be solicited and recorded for all subjects from the time of signing ICF B until termination of participation:

- Body temperature (and body location of measurement)
- Feverishness/subjective fever
- Chills

- Cough (and whether productive or nonproductive)
- Difficulty breathing
- Runny nose
- Nasal congestion
- Sneeze
- Nose dryness
- Nose bleed
- Sore throat
- Catarrhal nasopharynx
- Headache
- Confusion
- Convulsions/seizures
- Fatigue/malaise
- Muscle aches
- Pink or red eyes
- Drainage from eyes
- Swollen eyelids (conjunctivitis)
- Ear pain or discharge
- Rash
- Abdominal pain
- Diarrhea
- Vomiting

Evaluations will be made daily by a clinician on Day S1, Days 0-7 and Days 28-35 while a subject is admitted in the isolation unit and on Day 56. Evaluation must be made both prior to and after administration of dose one of study vaccine or placebo on Days 0 and 28. Reported signs and symptoms will be recorded by the subject on diary card on days after discharge from the isolation unit until termination of participation in the study.

8.1.3 Medical History

At enrollment, medical histories must be thoroughly reviewed through interview with the subject. The following medical conditions, especially, will be assessed:

- Current or recent (within two weeks of enrollment) acute respiratory illness with or without fever.
- Recent vaccination history.
- Practice of nasal irrigation on a regular basis within the past six months or has engaged in nasal irrigation within two weeks prior to enrollment.
- Recent history of frequent nose bleeds (>5 within the past year).
- Clinically relevant abnormal paranasal anatomy.
- Recent history (within the past month) of rhino or sinus surgery, or surgery for any traumatic injury of the nose.
- Recent receipt of immune globulin or other blood products, or injected or oral corticosteroids or other immune modulator therapy within 6 weeks (before) of enrollment.

- Hypersensitivity of any kind
- Asthma
- Tuberculosis
- Clinically relevant history of renal, gastrointestinal, cardiovascular, hematological, dermatological, endocrine, neurological or immunological diseases.
- Seizures, including history of febrile seizures, or any other neurologic disorder.
- Known or suspected immunologic function impairment of any kind and/or known HIV infection.
- Known HBV or HCV infection.
- Mental illness.
- Alcohol or drug use.
- Medications taken in the past year (including trade name, dosing or change in dosing, indications, start date, and termination date)
- For women, pregnancy, menstrual and contraceptive history and/or history of surgical sterility.

8.1.4 Physical Examination

Qualified study clinicians will conduct a physical examination of all subjects. This physical examination will include the following:

- Recording of general subject appearance
- Physical examination of all organ systems. This includes, without limitation, the following:
    - neurologic examination, including cranial nerve examination
    - chest auscultation
- Measurement of the following vital signs:
    - height
    - weight
    - body temperature (and body location of measurement)
    - blood pressure
    - pulse/heart rate
    - respiratory rate

Physical examinations will be made daily by a clinician on Day S1, Days 0-7 and Days 28-35 while a subject is admitted to the isolation unit and on Day 56. Evaluation must be made prior to administration of dose of study vaccine or placebo on Days 0 and 28.

8.1.5 ENT Examination

Qualified ENT physicians will conduct an ENT examination of all subjects. This examination will include the following:

- Inspection of the external ear.
- Examination of the nasal cavity with a Thudichum speculum
- Examination of the pharynx with a bright torch.
- Examination of the ear canal and tympanic membranes by otoscopy

ENT examination will be made on Days 0, 7, 28, 35 and 56. Evaluation must be made prior to administration of study vaccine or placebo on Days 0 and 28. Basic ENT examination may also be made by regular clinic staff during daily physical examinations of subjects; however, ENT specialists will only perform ENT exams on days specified.

8.2 Laboratory Evaluations

8.2.1 Clinical Laboratory Evaluations

Procedures for all laboratory assays will be specified in detailed protocols maintained at the RII. Normal values or ranges will be specified for all tests prior to enrollment of any subject.

8.2.1.1 Serology for Chronic Viral Infections

Random blood specimens will be evaluated for the following using tests qualified in the Russian Federation:

- HIV antibodies (ImmunoComb® II HIV 1&2 BiSpot Orgenics (Israel)
- HBV surface antigen (HBsAg) (Test-system for HBsAg detection: Access 2 Analyzer manufactured by BeckmanCoulter Inc.(USA))
- HCV antibodies using quality analysis with positive results confirmed by ribonucleic acid (RNA) determination in quantitative PCR analysis (Hepatitis C virus antibodies: Access 2 Analyzer (CLUA) manufacuctured by BIO-RAD Laboratories (CLUA).

Specimen collection will occur on Day S1. Determinant results of a subject's testing must be obtained from the laboratory prior to administration of any study vaccine or placebo.

8.2.1.2 Pregnancy Test

In order to confirm pregnancy status of females, qualitative human chorionic gonadotropin (hCG) test will be done on a random urine sample collected in the morning.

Specimen collection will occur on Days 0, 28 and 56. Results of testing for a subject must be complete and determinant prior to administration of any study vaccine or placebo on that day or prior to termination of subject participation in the study.

8.2.1.3 Comprehensive Metabolic Panel

Random blood specimens will be evaluated for the following using

Thermo Fisher Scientific Konelab PRIME 30 (USA, Finland) automated biochemical analyzer licensed for application in the Russian Federation:

Glucose

- Calcium
- Albumin
- Total Protein
- Sodium
- Potassium

- Carbon dioxide, bicarbonate
- Chloride
- Blood Urea Nitrogen (BUN)
- Creatinine
- Alkaline phosphatase (ALP)
- Alanine aminotransferase (ALT, also called SGPT)
- Aspartate aminotransferase (AST, also called SGOT)
- Total bilirubin

Test systems:

- Creatinine, Thermo Fisher
- ALaT, Thermo Fisher
- ASaT, Thermo Fisher
- Alkaline phosphatase, Thermo Fisher
- Sodium, Thermo Fisher
- Calcium, Thermo Fisher
- Potassium, Thermo Fisher
- Clorides, Thermo Fisher
- Calibrator -1, Ion-Selective Electrodes (ISE), Thermo Fisher
- Specitrol, Thermo Fisher
- Total bilirubin, Thermo Fisher
- Albumin, Thermo Fisher
- Total protein, Thermo Fisher
- Multicuvettes for Konelab, Thermo Fisher
- Glucose, Thermo Fisher
- Blood urea nitrogen (BUN), Thermo Fisher

Specimen collection will occur on Days S1, 0, 7, 28, 35 and 56. Results of testing for subject must be complete and determinant prior to administration of any study vaccine or placebo on that day or prior to termination of subject participation in the study.

8.2.1.4 Complete Blood Count with Differential

Random blood specimens will be evaluated for the following using tests qualified in the Russian Federation:

- Number of white blood cells (WBC), with differential (percentage of neutrophils, eosinophils, basophils, lymphocytes, and monocytes)
- Number of red blood cells (RBC)
- Hemoglobin content (Hgb)
- Hematocrit (Hct)
- Mean corpuscular volume (MCV)
- Mean corpuscular hemoglobin (MCH)
- Mean corpuscular hemoglobin concentration (MCHC)
- Platelet count and volume

- Erythrocyte sedimentation rate (ESR)

Test-systems:

- Diluent to be utilized in the automated hematological analyzer Sysmex KX-21, CELLPAC PK-30 L, Sysmex Europe GMBH
- Lysis reagent - STROMATOLYSER-WH, CE 500 mlx3, SWH-200A, R52/5300, Sysmex Europe GMBH for the hematological analyzer Sysmex KX-21
- Detergent - CELLCLEAN-CL-50 Cat. 834-0162-1, Sysmex Europe GMBH for the hematological analyzer Sysmex KX-21
- Calibration blood for the automated analyzer KX-21 REF-82003-1

Specimen collection will occur on Days S1, 0, 7, 28, 35 and 56. Results of testing for a subject must be complete and determinant prior to administration of any study vaccine or placebo on that day or prior to termination of subject participation in the study.

8.2.1.5 Urinalysis via Dipstick

Midstream urine specimens will be evaluated for the following using dipstick tests provided by PVS:

- pH
- Specific gravity
- Protein
- Glucose
- Ketones
- Nitrites
- Leukocyte Esterase

Specimen collection will occur on Days S1, 0, 7, 28, 35 and 56. Results of testing for a subject must be complete and determinant prior to administration of any study vaccine or placebo on that day or prior to termination of subject participation in the study.

8.2.2 Special Assays

Procedures for all laboratory assays will be specified in detailed protocols maintained at the either RII or IEM.

8.2.2.1 Detection of Shed Virus by rRTPCR

Nasal swab (and conjunctival) swab specimens will be tested for evidence of influenza virus using rRTPCR. These assays will be conducted by RII.

Nasal swab specimen collection for this purpose will occur daily on Days 0-7 and Days 28-35, while a subject is admitted to the isolation unit. Collection must occur both prior to and at least 6 hours after administration of study vaccine or placebo on Days 0 and 28. For nasal swabs collected on Days 1-6 and Days 29-34, results (influenza A positive or negative) of testing for a subject must be complete and determinant within 30 hours of specimen

collection. For nasal swabs collected on Day 7 and Day 28, results (influenza A positive or negative) of testing for a subject must be complete and determinant by 16:00 on that day. Confirmation of subtype should occur within 72 hours of specimen collection.

Conjunctival swab specimen collection will occur only for those subjects who develop conjunctivitis. Conjunctival swab specimens will be collected only on the first day of appearance of conjunctivitis. Results (influenza A positive or negative) of testing for a subject must be complete and determinant within 30 hours of specimen collection. Confirmation of subtype should occur within 72 hours of specimen collection.

Nasal swab specimens collection for rRTPCR testing will only occur past Day 7 or past Day 28 if a subject is requested to remain admitted to the isolation unit because a nasal (or conjunctival) swab specimen was influenza A positive during the final two planned days of stay in the isolation unit after each dose (Days 6 or 7 or Days 34 or 35).

In order to maintain isolation unit staff blinding throughout the study, one designated investigator will receive all daily results of rRTPCR testing. This investigator will maintain these results in a secure location and will not share results with other investigators or study staff. Likewise, laboratory personnel conducting the rRTPCR testing will not share the results with others not designated to receive these results. Only in the event that a subject prematurely leaves the isolation unit for any reason may any subject’s influenza A positivity be revealed. The designated person holding these results will not participate in study evaluations of safety. This designated person will be consulted on Day 7 and Day 35 in order to confirm that no subject has influenza A positivity in a nasal swab specimen collected in either of the previous two days.

Nasal swab specimen collection must ALWAYS occur at least one hour prior to any nasal wick specimen collection that occurs thereafter on that day and at least 7 hours after any previous nasal wick specimen collection on that day.

8.2.2.2 Isolation of Shed Influenza Virus in Chicken Embryos

Nasal swab specimens will be tested for presence of shed influenza virus by inoculation into 10-day old chicken embryos. Three passages in eggs will be performed – initial passage with nasal swab specimens and two blind passages if necessary. Positive samples will be tested for their infection titre in eggs. These assays will be conducted by IEM.

For these assays, nasal swab specimens collected on Days 1, 2, 3, 5, 7, 29 and 31 will be utilized.

Virus isolated from chicken embryos will be amplified by polymerase chain reaction (PCR) and sequenced in order to determine whether attenuation mutations have been maintained. These procedures will also be conducted by IEM.

Virological assays

Collection of nasal swab specimens will be performed with sterile dry applicators (COPAN Diagnostics, Inc.) using standard procedure in accordance with MG 4.2.2136-06. Following

collection of the material the applicator will be placed into the sterile tube containing transport medium (COPAN).

Virus isolation will be performed in accordance with the WHO manual on animal influenza, 2002). Normally developing 10-11-day-old chicken embryos (Bred Livestock Farm Ltd. "Nasia", Leningrad Region) will be used. Embryos will be inoculated through allantoic cavity route (each specimen applied for 3 embryos) and incubated for 3 days at temperature 32-34°C. The HA positive allantoic fluid with 1% chicken erythrocytes will be further used for typing and sequencing of the virus isolate obtained. In case of negative HA results, blind passage will be performed (3 days incubation at temperature 32- 34°C). When getting negative results the second blind passage will be performed and after that the sample will be evaluated as negative.

Testing of clinical specimens (nasal and conjunctival swabs) will be done by real-time RT-PCR (Rotor-Gene 6000, Australia) using AmpliSense reagent kit for influenza A virus DNA amplification, and by real-time PCR with hybridization fluorescent detection - INFLUENZA Test-System for PCR Detection and Differentiation of Avian Influenza Virus (the Federal Central Research Institute of Epidemiology under Ministry of Public Health of Russia), for identification of influenza A virus H5, H7 and H9 subtypes in biological material.

8.2.2.3 Mucosal Anti-influenza IgA

Nasal wick specimens will be tested by quantitative EIA for the presence of anti-influenza IgA according to the CDC protocol (Centers for Disease Control and Prevention, Atlanta, USA).. These assays will be conducted by IEM.

For EIA procedure the following will be used:

1. Antigen – 16 HAU of the vaccine strain A/17/Mallard/ Netherlands/00/95 (H7N3);
2. Phosphate buffer solution (PBS) ("Biolot", Russia);
3. TMB-substrate («BD bioscience», USA)
4. Tween-20 ("Biolot", Russia);
5. Bovine serum albumin (BSA, fraction V) ("Biolot", Russia);
6. Horseradish peroxidase-conjugated rabbit affinity purified anti-human IgA antibodies (Sigma, США);
7. Disposable EIA polysterene plates («Medpolymer», Russia).

Vaccine immunogenicity will be evaluated due to the following criteria:

1. Changes in serum IgG and secretory IgA geometric mean titers (GMT) pre- and post- vaccination;
2. Determination of proportions of subjects with antibody response (seroconversions) (4-fold and greater increase in influenza antibody titer).

For these assays, nasal wick specimen collection will occur on Days 0, 28, and 56. Collection must occur at least one hour prior to administration of study vaccine or placebo on Days 0 and 28.

8.2.2.4 Serum Anti-influenza IgA and IgG

Serum specimens will be tested for the presence of anti-influenza IgA and IgG using quantitative EIA according to the CDC protocol (Centers for Disease Control and Prevention, Atlanta, USA). These assays will be conducted by IEM.

For EIA procedure the following will be used:

1. Antigen - 16 HAU of the vaccine strain A/17/Mallard/ Netherlands/00/95 (H7N3);
2. Phosphate buffer solution (PBS) ("Biolot", Russia);
3. TMB-substrate («BD bioscience», USA)
4. Tween-20 ("Biolot", Russia);
5. Bovine serum albumin (BSA, fraction V) ("Biolot", Russia);
6. Horseradish peroxidase-conjugated rabbit affinity purified anti-human IgA antibodies (Sigma, США);
7. Disposable EIA polysterene plates («Medpolymer», Russia).

Vaccine immunogenicity will be evaluated due to the following criteria:

1. Changes in serum IgG and secretory IgA geometric mean titers (GMT) pre- and post- vaccination;
2. Determination of proportions of subjects with antibody response (seroconversions) (4-fold and greater increase in influenza antibody titer).

For these assays, serum specimen collection will occur on Days 0, 28 and 56. Collection must prior to administration of study vaccine or placebo on Days 0 and 28.

8.2.2.5 Serum Antibody to Influenza Virus Detected by HAI

The HAI assay is the most frequently used serologic test for determining immunologic response to influenza vaccination. Briefly, sera are pretreated with heat and receptor-destroying enzyme to reduce non-specific inhibition. In 96-well microtiter plates, serum specimens are then serially diluted with doubling dilutions and incubated with standardized titers of influenza virus antigens representing vaccine strains. After incubation, chicken- or human-derived red blood cells are added to individual wells and allowed to sediment. Plates are tipped, and RBCs that have settled will produce a streak (RBCs run or flow), indicating inhibition of hemagglutination and the presence of serum antibodies against the influenza test strain. Wells with lattice formation (hemagglutination) will not streak. When processing paired sera (pre- and post-vaccination), an increase in antibody titer indicates response to vaccination. Serum specimens will be tested for the presence of HAI antibodies to influenza by IEM. Determination of specific anti-influenza antibodies using hemagglutination inhibition test (HI) test will be performed by IEM in accordance with the conventional technique (MG 3.3.2.1758-03. Methods to determine quality characteristics of immunobiological preparations for influenza prevention).

For HI test procedure the following will be used:

1. Antigen - 4 HAU of the vaccine strain A/17/Mallard/ Netherlands/00/95 (H7N3);

2. Human erythrocytes 0(I) Rh+ from erythrocyte concentrates unsuitable for transfusion and acquired in the Municipal blood transfusion station under St.-Petersburg Health Department;
3. Phosphate buffer solution (PBS) ("Biolot", Russia);
4. Disposable polysterene plates for immunological assays («Medpolymer», Russia).

Vaccine immunogenicity will be evaluated due to the following criteria:

1. Seroconversion factor – increase of antibody geometric mean titers (GMT) on Day 21 in comparison with initial antibody levels in terms of "fold" increase.
2. Seroconversion level – percent of volunteers showing higher than 4-fold increase of antibody titer in comparison with initial values.
3. Seroprotection level – percent of volunteers showing antibody titer exceeding 1:40 on Day 21 post-vaccination.

For these assays, serum specimen collection will occur on Days 0, 28 and 56. Collection must prior to administration of study vaccine or placebo on Days 0 and 28.

8.2.2.6 Serum Antibody to Influenza Virus Detected by Microneutralization Assay

The microneutralization assay is an alternative test for determining immunologic response to vaccination. Serum, along with live influenza virus, is added to a culture of Madin-Darby Canine Kidney (MDCK) cells. Titers of neutralizing antibodies are expressed as an amount of the greatest dilution giving a neutralization of 50% of tissue cytopathic effects of the virus in the tissue culture ($TCID_{50}$). Serum specimens will be tested for the presence of neutralizing antibodies to influenza by microneutralization by IEM.

Vaccine immunogenicity will be evaluated due to the following criteria:

1. Seroconversion factor – increase of antibody geometric mean titers (GMT) on Day 21 in comparison with initial antibody levels in terms of "fold" increase.
2. Seroconversion level – percent of volunteers showing higher than 4-fold increase of antibody titer in comparison with initial values.
3. Seroprotection level – percent of volunteers showing antibody titer exceeding 1:40 on Day 21 post-vaccination.

For these assays, serum specimen collection will occur on Days 0, 28 and 56. Collection must prior to administration of study vaccine or placebo on Days 0 and 28.

8.2.2.6.1 Microneutralization assay procedure

Step 1. Determination of virus infectious activity in MDCK cells

MDCK cell culture in concentration of 200 000 cells/ml will be dispensed into 96-well cell culture plates (Nunc or Sarstedt) and grown up to monolayer in $C0_2$- incubator at temperature (36 ± 0,5)°C over 1-2 days. Prior to titration procedure, cells will be washed 2 times with a serum-free Eagle medium and after that 100 μl of Eagle medium containing TPCK-trypsin (2 μg/ml) will be added into each well.

10-fold virus dilutions in a serum-free Eagle medium containing TPCK-trypsin in concentration of 2 μg/ml will be prepared in a row of 6 vials each containing 450 μl of medium supplemented with TPCK-trypsin. The virus in a volume of 50 μl will be added into the first vial and

after thorough mixing 50 μl of dilution will be transferred into each next vial changing pipettes or tips with each dilution.

50 μl of each prepared virus dilution starting from the last one will be transferred into 4 rows of culture plate wells coated by MDCK cell monolayer.

For control of cell culture (CC) condition, 50 μl of the Eagle medium containing TPCK-trypsin (2 μg/ml) will be added into each of 4 plate wells.

Plates are place into C02 incubator at temperature (36 ± 0,5)°C.

Virus titer will be estimated in ELISA after 48 h. For this purpose cultural fluid will be removed from all wells, cells will be twice washed with phosphate buffered saline (PBS), pH 7.2-7.4, and fixed with 80% cold acetone (during 5-20 min at 0 °C).

100 μl of peroxidase conjugated monoclonal antibodies to influenza A virus nucleoprotein (NP) diluted 1/4000 by PBS containing 5% non-fat cow milk (PBS-M) will be added into each well. Plates will be incubated for 1 h at 37°, followed by 4-fold washing with PBS and to reveal peroxidase reaction 100 μl of substrate mixture containing 0.02% H_20_2 and 0.1 mg/ml of 3,3',5,5'-tetramethylbenzidine (TMB) in acetate-citrate buffer (pH 5.0) will be added per well. Plates will be incubated for 15 min. After the reaction is stopped (by adding 50 μl of 2N H_2SO_4 per well) optical density will be measured by spectrophotometer at a wavelength of 450 nm (OD450).

The virus titer will be defined as the last dilution with OD450 values at least twice exceeding CC ones.

The virus titer will be calculated in accordance with the Reed-Muench method.

Virus dilution containing 100 TID_{50} of the virus in 50 μl of the medium (ELISA data) will be determined.

Step 2. Neutralization assay procedure

Tested sera will be diluted 10-fold with trypsin-free Eagle medium and heated during 30 min at temperature 56°C. Two-fold serial dilutions of heated sera in Eagle medium will be prepared in 100 μl aliquots (1:20-1:1280). In addition, virus working dilution (VWD) containing 100 TID_{50} in 50 μl of Eagle medium supplemented with TPCK-trypsin in concentration of 4 μkg/μl will be prepared. Equal volumes of serum dilutions ranging from 1:10 to 1:1280 and VWD will be mixed. Obtained mixtures will be incubated over 1 h at 37°C and then added in amount of 100 μl into each well coated with monolayer of preliminary washed MDCK cells. A total of 50 μl of Eagle medium supplemented with TPCK-trypsin (2 μkg/μl) will be added previously into each well.

The following control tests should be performed for each plate:

1. CC- 4 wells coated with cell monolayer containing 150 μkg of virus-free Eagle medium supplementated with TPCK-trypsin (2 μkg/μl).
2. Virus dose control (VDC). Determination will be performed by repeated titration of VWD in initial condition and in dilutions $10^{"1}$, $10^{'2}$, $10^{'3}$. VWD dilutions will be dispensed in 50 μl per well into 2 rows of wells with addition of 100 μl of Eagle medium supplemented with TPCK-trypsin (2 μkg/μl)).
3. Serum control (SC). Each of tested sera in dilution of 1/10 (50 μl) will be dispensed into wells containing cell monolayer with addition of 100 μl of Eagle medium (virus-free).

Plates will be incubated in $C0_2$ incubator at temperature (36 ± 0,5)°C during 48 h. Medium will be completely removed, monolayer will be fixed with 80% cold acetone over 15-20 min, and ELISA will be performed as indicated in Step 1.

Step 3. Data analysis

Neutralizing antibody titer is defined as the last serum dilution when OD450 value is lower than cut-off value (CV) determined according to the following formula:

$$CV = \frac{VWD\ OD450 - CC\ OD450 + CC\ OD450 + KK}{2},\ \text{where}$$

VWD OD450 - OD450 mid-value in control wells containing virus working dilution, CC OD450 - OD450 mid-value in control wells containing non-infected cell culture

Four-fold and greater increase of antibody titers in reconvalescent sera in comparison with a serum sample obtained during acute illness stage is considered as a diagnostically significant antibody level.

Notes: When determination of virus dose is correct, positive signal must be registered at VWD dilutions 10 ~^{1}, 10 ~ ^{2} and be missing at dilution 10~^{3}.

In case virus dose control fails to confirm 100 TID_{50} of virus dose, the test will be repeated.

8.2.3 Nasal and Conjunctival Swab Specimens

8.2.3.1 Collection of Nasal Swab Specimens

Nasal swab specimens for both rRTPCR assays and virus isolation on chicken embryos will be collected using Dacron flocked swabs with a nylon shaft, since these swabs do not interfere with PCR assays. The swab should be inserted into the nostril, parallel to the palate, and left in place for a few seconds. The swab should then be slowly withdrawn with a rotating motion. Specimens should be obtained from both nostrils using two swabs. After collection, the tips of the swabs should be placed into a plastic vial or tube containing 1.0 ml of virus transport medium and the applicator sticks broken off or bent into the tube so that the tube can be sealed. Swab specimens will be affixed with the subject's study participant number that links the specimen to the subject and labeled as to specimen type, specimen collection date, and specimen number and time-point. All specimen collection will be conducted by trained individuals.

8.2.3.2 Collection of Conjunctival Swab Specimens

Conjunctival swab specimens will also be collected using Dacron flocked swabs with a nylon shaft. The subject should be seated with the head comfortably supported and titled back. The subject should be instructed to look up to prevent corneal damage. Ensuring that the swab does not touch the subject's skin surface by pulling down slightly on the lower eyelid or cheek, the swab should be passed firmly along the fornix, from inner to outer canthus, with a rotating motion. After collection, the tip of the swab should be placed into a plastic vial or tube containing 2-3 ml of virus transport medium and the applicator sticks broken off or bent into the tube so that the tube can be sealed. Each swab specimens will be affixed with the subject's study participant number that links the specimen to the subject and labeled as to

specimen type, specimen collection date, and specimen number and time-point. All specimen collection will be conducted by trained individuals.

8.2.3.3 Conditions for Transport, Processing and Storage of Swab Specimens

Collected nasal and conjunctival swab specimens will be stored at refrigerator temperatures in the isolation unit until transport to the RII NIC laboratory. Specimens will be securely transported on ice to the RII NIC laboratory each day. In the RII NIC laboratory, vials/tubes containing swab specimens will be opened and swab specimen applicator sticks will be agitated to remove collected material, fluid will be expressed, and swabs discarded. Only for swab specimens collected on Days 1, 2, 3, 5, 7, 29 and 31, each swab specimen will be divided into two aliquots of approximately 0.5 ml, one tube for RII and one tube for IEM. For specimens to be tested by rRTPCR at RII, testing should be done within 30 hours of collection. Specimen material not used for virus identification will be stored at –70˚C in each laboratory. Handling and storage of specimens will emphasize avoidance of freeze-thaw cycles.

8.2.4 Nasal Wick Specimens

8.2.4.1 Collection of Nasal Wick Specimens

For nasal wick specimen collection, Merocel® (biocompatible polyvinyl-alcohol sponge) wicks will be used. With the subject sitting in a comfortable position with the head tilted slightly back, wicks will be inserted 2-3 cm into the subject’s nostrils along the lateral nasal walls up to the inferior nasal conches. Wicks should remain in the nasal passages for 5 minutes before being slowly withdrawn using the attached drawstring. The wicks will then be placed into a plastic vial containing 0.5 ml of sterile phosphate-buffered saline and the drawstrings trimmed to allow closing of the vial.

8.2.4.2 Conditions for Transport, Processing and Storage of Nasal Wick Specimens

Vials containing wicks will be transported to the IEM laboratory at refrigerator temperatures (+2°C to +8°C). The wick will be pulled out of the vial and placed into a standard 1 ml pipette tip. The tip will be inserted back into the vial and centrifuged for 10 minutes at 3000 rpm, +4ºC. After centrifugation the tip with the wick will be discarded. The vial containing the residual nasal wick specimen will be stored at –70ºC until analysis (but no longer than 3 months). Handling and storage of nasal wick specimens will emphasize avoidance of freeze-thaw cycles.

8.2.5 Blood Specimens

Blood will be collected for testing in multiple assays (serum for the following: CMP; HIV, HBV and HCV serology; anti-influenza IgA and IgG serology; HAI antibody serology; and neutralizing antibody serology. Whole blood for the following: CBC with differential and isolation of PBMCs for cellular immune response studies.).

8.2.5.1 Collection of Blood

Following universal precautions, blood will be collected from the forearm by venipuncture into vacutainer tubes. Blood for CMP, serology for chronic viral infections, and serology for anti-influenza antibodies must be collected in tubes appropriate for collection of serum (serum separator tubes). Blood for CBC with differential and isolation of PBMCs must be collected in tubes appropriate for collection of whole blood (tubes containing potassium EDTA or appropriate anti-coagulant). Blood should be held at room temperature (+18°C to +25°C until processing). Volumes of blood required for the different categories of assays at different time-points are show in the below table.

	Collection Tube	Volume of blood	S1: Day of 1st Screening	S2/D0 (pre-vac)	D7	D28 (pre-vac)	D35	D56
Whole blood for CBC with differential	Anti-coagulant	3 ml tube	✓	✓	✓	✓	✓	✓
Serum for serology for HIV, HBV, HCV infection	Serum separator	5 ml tube	✓					
Serum for CMP	Serum separator	3 ml tube	✓	✓	✓	✓	✓	✓
Serum for anti-influenza serologic assays	Serum separator	7 ml tube		✓		✓		✓
Whole blood for isolation of PBMCs	Anti-coagulant	40 ml		✓		✓		✓

(The total volume of blood planned to be collected from each subject during the course of the study will be less than 200 milliliters.)

Processing of Whole Blood for CBC with Differential

Immediately after collection, the blood specimen tube will should be gently inverted 7 or 8 times, labeled with the subject's study participant number, the collection date, the blood specimen number, and submitted immediately to the RII laboratory for processing. Whole blood for CBC with differential will not be divided.

8.2.5.2 Processing of Whole Blood for Isolation of PBMCs

For PBMCs isolation from relatively large volumes of blood (>30 ml) Leucosep tubes will be used. The tubes contain a porous barrier that enables the blood sample to be poured onto the Ficoll gradient, thereby eliminating the need to gently layer on the sample. RII will process whole blood for isolation of PBMCs using a strict SOP. Collected PBMCs will be immediately resuspended in a freezing solution, placed in cryovials and the cryovials placed into a freezing container filled with isopropyl alcohol before freezing to -80°C. Freezing of PBMCs for storage and thawing of PBMCs for testing will also strictly follow an SOP.

8.2.5.3 Processing of Sera

Immediately after collection, the blood specimen tube will be inverted 5 times, labeled with the subject's study participant number, the collection date, the blood specimen number, and stood upright to clot for at least 30 minutes at room temperature before transport to the RII

laboratory for processing. At the laboratory, specimens will be centrifuged at 3000 rpm for 10 minutes before division.

8.2.5.3.1 Method for assessment of celluar immunity response

Cellular immunity response will be assessed by significant post-vaccination increase (%) of CD4 and CD8 T-cell level (higher than 2 standard deviations from mean pre-vaccination level).

Test will be performed using peripheral blood lymphocyte samples collected from volunteers to be assessed in BD FACS Canto2 flow cytofluorimeter (USA). Levels of vaccine strain specific cells will be measured by standard intracellular cytokine staining assay (Current Protocols in Immunology / Ed. John E. Coligan, Ada M. Kruisbeek, David H. Margulies. Wiley press. - 2004) following in vitro stimulation with the vaccine strain A/17/Mallard/ Netherlands/00/95 (H7N3).

For assay the following will be used:

1. Antigen - 16 HAU of the vaccine strain A/17/Mallard/ Netherlands/00/95 (H7N3);
2. Phosphate buffer solution (PBS) (“Biolot”, Russia);
3. RPMI 1640 nutrient medium (“Biolot”, Russia);
4. Fetal bovine serum (FBS) (“Biolot”, Russia);
5. Histopaque-1077 gradient (Sigma, USA);
6. Paraformaldehyde (PFA) («Vecton)», Russia);
7. BD Cytofix/Cytoperm™ intracelluar staining kit («BD Bioscience», USA);
8. Fluorochrome labeled monoclonal antibodies to CD3, CD4, CD8, IFN-г, CCR-7, CD45RA («BD Bioscience», USA; «Beckman Coulter», USA).

8.2.5.4 Division of Sera

Serum specimens for anti-influenza serologic assays will be divided. Specimen division should be performed participant by participant to avoid mixing blood tubes. The procedure should be carried out as follows:

- After centrifugation, the person in charge of dividing specimens should carry out the operation by taking the tubes one by one from the centrifuge.
- The operator will only place in a rack the number of tubes (5 tubes) necessary for the division of one subject’s specific blood specimen and will affix the completed labels onto the tubes checking the study participant number, collection date and blood specimen number.

- Each serum specimen will be divided into 6 aliquots as follows:
  - 1st aliquot: 0.5 ml for IgA and IgG EIA assays
  - 2nd aliquot: 0.5 ml for HAI assays
  - 3rd aliquot: 0.5 ml for microneutralization assays
  - 4th aliquot: 0.5 ml as first back-up
  - 5th aliquot: remaining serum as second backup
- The study participant number, date of collection, blood specimen number, number of divisions obtained, and the date and time of division will be specified on a serologic specimen log form. On this form, comments may be made on the quality of specimens (e.g., hemolyzed, contaminated, etc.).
- The next blood tube should be taken out of the centrifuge at the end of this procedure.

8.2.5.5 Conditions for Transport and Storage of Sera

Serum specimens for anti-influenza serologic assays will be immediately frozen to –20˚C after division. Frozen sera will be at all times maintained in a frozen state during transport between RII and IEM. All handling will be done to prevent unnecessary freeze-thaw cycles.

8.2.6 Urine Specimens

Urine will be collected for urinalysis via dipstick and for pregnancy testing. For urinalysis, a clean-catch, midstream urine specimen collected after cleansing the external urethral meatus should be obtained.

No urine specimens will be stored after testing.

9 Assessment of Safety

9.1 Specification of Safety Parameters

The safety profile will be parameterized as the proportion of subjects experiencing adverse events (AEs) of the following four categories:

- Immediate reactions occurring within two hours of administration of any dose, measured as observed by study staff or reported by the subject to study staff.
- Adverse events commonly associated with intranasal vaccination (solicited local and systemic reactions) occurring greater than two hours after administration of any dose of study vaccine or placebo through 7 days following any dose, measured as observed by study staff or reported by the subject to study staff.
- All other adverse events (including unsolicited events) occurring during the 7 days following any dose, measured as observed by study staff or reported by the subject to study staff. This includes abnormal laboratory findings from blood and urine specimens collected on Days 7 and 35.

- All serious adverse events (SAEs) occurring within 4 weeks of receipt of any dose, as observed by study staff, reported by the subject to study staff, or noted by the subject on a diary card. This includes abnormal laboratory findings from blood and urine specimens collected on Days 28 (pre-vaccination) and 56.

9.2 Methods and Timing for Assessing and Recording Safety Parameters

9.2.1 Post-administration Reactions

9.2.1.1 Immediate Reactions

All participants will be observed for immediate reactions for two hours after administration of study vaccine or placebo, with appropriate medical treatment readily available in case of an anaphylactic reaction following the administration of study vaccine or placebo. Immediate reactions will be assessed by a study physician or appropriately trained medical staff. All reactions that occur during this time will be recorded on the CRF. Any immediate reaction which meets the criteria for an SAE must also be documented on an SAE form.

As deemed appropriate by a study clinician, subjects with immediate post-administration reactions will be excluded from receipt of secondary doses of study vaccine or placebo and withdrawn from the study.

9.2.1.2 Local and Systemic Reactions

Specific local and systemic reactions will be solicited while subjects are admitted to the isolation unit. These specific reactions (signs and symptoms) will be graded by a study clinician using a standardized data collection instrument. Grading will occur using predefined scales based on functional assessment or magnitude of reaction, where available. Where grading scales are not provided, the reaction will be graded for severity based on interference with subject functionally, as for all other AEs (See Section 9.2.2). Absence of the sign or symptom will always be explicitly noted and graded as a zero.

Local reactions:

- Dryness of the nose
- Nose bleeds
- Ticklish throat
- Nasal congestion
- Runny nose
- Catarrhal nasopharynx

Systemic Reactions:

- Body temperature (and body location of measurement)
- Feverishness/subjective fever
- Chills
- Cough (and whether productive or nonproductive)

- Difficulty breathing
- Sore throat
- Headache
- Confusion
- Convulsions/seizures
- Fatigue/malaise
- Joint aches
- Muscle aches
- Pink or red eyes
- Draining eyes
- Swollen eyelids (conjunctivitis)
- Ear pain or discharge
- Rash
- Abdominal pain
- Diarrhea
- Vomiting

9.2.2 Adverse Events

An AE is defined as any untoward medical occurrence in a patient or clinical investigation participant administered a pharmaceutical product, which does not necessarily have a causal relationship with this treatment. An AE can therefore be any unfavorable and unintended sign (including an abnormal laboratory finding), symptom, or disease temporally associated with the use of a medicinal product, whether or not considered related to the medicinal product. The occurrence of an AE might come to the attention of study personnel during study visits and interviews of a study participant presenting for medical care.

Information to be collected on AEs includes event description, time of onset, clinician's assessment of severity, relationship to study product (assessed only by those with the training and authority to make a diagnosis), and time of resolution/stabilization of the event. During hospitalization, in case of fever >38.0°C the body temperature should be measured every 2 hours until resolution (i.e. ≤38.0°C).

Any medical condition, including any Grade 1 physical examination finding or clinical laboratory test result, that was present at the time that the subject was enrolled should not be reported as an AE. However, if this condition deteriorates (becomes Grade 2 or higher) during the study, it should be recorded as an AE.

AEs should always be graded for severity and relationship to vaccine.

Severity of Event: All AEs, including clinical laboratory test results, will be assessed by a study clinician to quantify severity using a protocol-defined grading system. For this protocol, the US Food and Drug Administration “Toxicity Grading Scale for Healthy Adult and Adolescent Volunteers Enrolled in Preventive Vaccine Clinical Trials” will be used (see Attachments), except for fever (see below). For events not included in this grading system, the following guidelines will be used to quantify intensity:

0=None; lack of sign or symptom; normal

1=Mild: events require minimal or no treatment and do not interfere with the subject's daily activities.

2=Moderate: events result in a low level of inconvenience or concern to the subject with therapeutic measures. Moderate events might cause some interference with functioning.

3=Severe: events interrupt the subject's functioning and might require systemic drug therapy or other treatment. Severe events are usually incapacitating.

4=Life threatening: any adverse experience that places the subject, in the view of the investigator, at immediate risk of death from the reaction *as it occurred*. (The investigator should *not* grade a reaction as life-threatening that had it occurred in a more severe form, might have caused death.)

For fever, as measured by axillary body temperature, the grading scale will be as follows:

0=none:	<37°C
1=mild:	37°C to 37.5°C
2=moderate:	37.6°C to 38.5°C
3=severe:	38.6°C to 40°C
4=life threatening:	>40°C

Changes in the severity of an AE should be documented to allow an assessment of the duration of the event at each level of intensity to be performed. AEs characterized as intermittent require documentation of onset and duration of each episode.

Relationship to Study Vaccines: The clinician's assessment of an AE's relationship to study vaccine is part of the documentation process, but it is not a factor in determining what is or is not recorded in the study. If there is any doubt as to whether a clinical observation is an AE, the event should be recorded. All AEs must have their relationship to study vaccine assessed using the terms "related" or "not related." To help assess, the following guidelines will used:

Related – There is a reasonable possibility that the study vaccine caused the AE. Reasonable possibility means that there is evidence to suggest a causal relationship between the study vaccine and the AE.

Not Related – There is not a reasonable possibility that the administration of the study vaccine caused the event.

All solicited and unsolicited AEs occurring during the study will be assessed and recorded. For clarity, unsolicited events includes those observed by study staff while the subject in admitted to the isolation unit and all AEs either recorded by the subject on diary card or reported by the subject during review of interim medical histories or clinical examinations.

9.2.3 Serious Adverse Events

All SAEs that occur during the trial will be captured on the CRF.

Serious Adverse Event: An SAE is defined as an AE that meets one of the following conditions:

- Death.
- Life threatening (subject at immediate risk of death.) *(The term "life-threatening" in the definition of "serious" refers to an event in which the patient was at risk of death at the time of the event; it does not refer to an event that hypothetically might have caused death if it were more severe).*
- Requires inpatient hospitalization or prolongation of existing hospitalization. *(Continuation of stay in the isolation unit beyond 7 days post-administration of either dose of study vaccine or placebo because of late detected viral shedding in a subject shall not be considered a "prolongation of existing hospitalization" for SAE recording or reporting.)*
- Results in congenital anomaly/birth defect. *(Only in the case of a woman becoming pregnant during the study period after administration of at least one dose of study vaccine. All pregnancies must be followed to term and outcome reported to the Sponsor, PVS and regulatory agencies.)*
- Results in a persistent or significant disability or incapacity.
- Important medical events that might not result in death, be life threatening, or require hospitalization might be considered SAEs when, based upon appropriate medical judgment, the event might jeopardize the well-being of the subject and require medical or surgical intervention to prevent one of the outcomes listed above. *(Medical and scientific judgment should be exercised in deciding whether reporting these events is appropriate.)*

All SAEs occurring within one month of any study vaccination must be reviewed and evaluated by a study clinician (SAE relationship to study vaccine must be evaluated as outlined in Section 9.2.3) and recorded on an SAE form and reported (as specified in Section 9.3). All such SAEs should also be followed until satisfactory resolution or until the investigator deems the event to be chronic or the patient to be stable.

9.2.4 Procedures to be Followed in the Event that of Abnormal Laboratory Test Values

To the extent possible, all normal ranges for clinical laboratory test results will be pre-specified. All abnormal laboratory test values must be documented as an AE, unless that value was Grade 1 at study entry (Day 0) and has not increased in severity grade. Abnormal test values may be considered an SAE if the value rises to the level of severe (Grade 4). Any value not normalizing by the end of the study (Day 56) should be followed by the investigator, as would be done under normal clinical care circumstances. After termination of the trial, the investigator should assure that the subject is referred for medical follow-up, as appropriate.

9.3 Reporting Procedures

9.3.1 Serious Adverse Events

All SAEs occurring within one month of any study vaccination must be documented and reported to PVS or its designate, even if the investigator considers that the SAE is not related to treatment. The study clinician will complete a **Serious Adverse Event Form** within the following timelines of such events:

- All deaths and immediately life-threatening events, whether related or unrelated, will be recorded on the Serious Adverse Event Form and sent by fax or email within 24 hours of site awareness.
- SAEs other than death and immediately life-threatening events, regardless of relationship, will be reported via fax by the site within 48 hours of becoming aware of the event.

PVS will be primarily responsible for medical monitoring of serious adverse events documented by the investigator. Details for how review of serious adverse events and other unanticipated problems will be conducted will be contained in an SOP prior to study initiation.

9.3.2 Regulatory Reporting

Collected SAEs and AEs will be reported to responsible ethical review committees according to their requested timelines. An SOP for reporting to the responsible committees will be developed with reporting requirements and timelines prior to study initiation. It will be the investigator's responsibility to assure that all reportable events are reported to the proper authority or to PVS and/or the Sponsor in a timely manner and according to the SOP. PVS and/or the Sponsor may assist the investigator with regulatory reporting, per the finalized SOP.

9.3.3 Reporting of Pregnancy

All pregnancies detected among women enrolled in the trial who receive at least one dose of study vaccine or placebo must be reported. All pregnancies must be followed to term and outcome reported to the Sponsor, PVS and regulatory agencies. Unblinding of allocation of treatment of female subjects who become pregnant will not occur until after study completion. No female subjects who become pregnant after receipt of study dose one will be allowed to receive study dose two, regardless of allocation of treatment. Such a subject should be withdrawn immediately from the trial.

9.4 Type and Duration of Follow-up of Subjects after Adverse Events

SAEs occurring within one month of any study vaccination will be recorded as indicated in the CRF. AEs likely to be related to the product whether serious or not, which persist at the end of the trial will be followed up by the investigator until their complete disappearance. The

investigator will document the date of final disappearance of the AE on a data clarification form.

9.5 Halting Rules

Given the long experience with LAIVs, no safety issues are anticipated which would result in the suspension of the study. However, as this study is a phase 1 trial, the trial might be suspended at any time by the Sponsor, PVS, the Russian Ministry of Health, the SMC, any ethical review committee overseeing this study, or by the investigator for any safety concern. This includes, for example, and without limitation, an SAE resulting in death, an unexpectedly high number of persons shedding virus after receipt of any dose, an unusually high rate of SAEs, or an unexpectedly early onset of substantial seasonal influenza virus circulation in the surrounding community. PVS may suspend the study in the event that study conduct is found to be below GCP standards.

Should an SAE occur which is likely related to administration of study vaccine, the decision whether the study should continue per protocol, proceed with caution, be suspended pending further investigation, be discontinued, or be modified and then proceed will be made by the Sponsor and PVS in consultation with the investigators, the SMC, and/or the RF MoH. However, no rules will be prespecified to define these conditions.

If the study is halted, subjects will be contacted immediately explaining why the study has been halted and the implications for their medical well-being.

In the event of the appearance of new data that indicate an increased level of risk to participating subjects, the clinical trial will be suspended until the Sponsor, PVS, the Russian Ministry of Health, the SMC, and all ethical review committees have reviewed relevant data and agreed that the trial may proceed.

9.6 Safety Oversight by a Safety Monitoring Committee

Additional safety oversight will be conducted by a Safety Monitoring Committee (SMC). The SMC will operate under rules of a SMC-approved charter that will be written or finalized at the organizing meeting of the SMC (first meeting). PVS will draft this charter, and the SMC will approve it, after any modification by the SMC. The draft charter will embody the elements of the following sections.

9.6.1 SMC Roles and Responsibilities

A local SMC will be constituted for independent safety review of this trial. The SMC will review individual and cumulative participant safety data from each study sub-cohort when making recommendations regarding the safe continuation of the study. SMC members will not participate in the study, nor will they fall under the direct supervision of any investigator participating in the study.

PVS will, prior to any review of data, confirm that no conflicts of interest exist with any SMC member. Interests that might create a potential conflict of interest should be disclosed to

PVS prior to any review of safety data. PVS will determine if the relationship is in conflict or gives the appearance of a conflict such that the individual should not serve on the SMC. PVS will determine how to handle such potential conflict. PVS might dismiss a SMC member in the event of an unmanageable potential conflict.

The SMC membership will be considered confidential until after the study is completed.

9.6.2 Study Materials for SMC Review

The primary focus of the SMC will be to independently review all SAEs and thoroughly investigate those considered unexpected. The SMC will accomplish this by evaluating all such events against the known or expected safety profile of the study vaccine. Clinical and laboratory data, clinical records, and other study-related records will be made available for SMC, as appropriate. If necessary, special reports will be prepared by the investigator or PVS.

It is the responsibility of the investigator and PVS to ensure that the SMC is apprised of all new safety information relevant to the study vaccines and the study. The SMC will receive all protocol revisions and might receive other documents related to the study.

9.6.3 Reports from the SMC

The following reports might be submitted by SMC: review report and immediate action report.

9.6.3.1 Review Report

According to pre-specified criteria agreed upon by PVS, the SMC will communicate in writing its findings and any concerns and recommendations to the Sponsor and PVS. Unless otherwise specified, the written report will then be forwarded to the investigator who must, in turn, submit the report as per local IRB and IEC policy.

9.6.3.2 Immediate Action Report

The SMC will notify the Sponsor and PVS of any findings of a serious and immediate nature including any recommendations to discontinue all or part of the trial. PVS will immediately inform the investigator. In addition to any verbal communications, recommendations to discontinue or substantially modify the design or conduct of a study must be conveyed in writing on the day of the SMC meeting where this recommendation is formally made. This written, confidential report might contain unmasked supporting data and should include the SMC’s rationale for the recommendations.

10 Clinical Monitoring

10.1 Site Monitoring Plan

Qualified and appropriately trained individuals from PVS or designated by PVS will carefully monitor the study. The study monitors will periodically contact the site and perform on-site visits. The extent, nature and frequency of site visits will be based on such considerations as study objectives, study design and complexity, and enrollment rate; periodicity and nature of monitoring activities will be described in the *Monitoring Plan.* PVS might also include representatives of IEM and/or Microgen in site contacts and visits, as appropriate, but will nevertheless keep officials from IEM and Microgen apprised of study progress.

10.1.1 Set-up Visit

The study monitor or PVS representative will contact the site prior to the start of the study to discuss the protocol and data collection procedures with site personnel. Prior to enrollment of subjects at the study site, specific regulatory documents must be available, including approvals from the independent ethics committee (IEC) at the Russian Ministry of Health and from institutional review boards of the participating institutions (see Section 1). Curriculum vitae for key investigators must also be available. PVS will inform the investigator of any additional documents that need to be provided.

10.1.2 Follow-up Visits

Monitoring will be conducted according to PVS's requirements. The individuals responsible for monitoring the study will periodically review the progress of the study and should have access to all records necessary to ensure the ethical and safety conduct of the study and the integrity/validity of the recorded data.

During sites visits and contacts, the monitor will:

- Check and assess the progress of the study.
- Review study data collected.
- Perform source data verification.
- Review regulatory files.
- Identify any issues and address their resolution.

This will be done in order to verify that:

- The data are authentic, accurate and complete.
- The safety and rights of subjects are being protected.
- The study is conducted in accordance with the approved protocol (and any subsequent amendment) and all applicable regulatory requirements.

As part of study conduct, the principal investigator agrees to allow the monitor or PVS representative direct access to all relevant documents and to allocate his/her time and the time of his/her staff to the monitor to discuss findings and any relevant issues.

The principal investigator also agrees to allow representatives of IEM and/or Microgen to occasionally accompany the monitor or PVS representative during site visits.

10.1.3 Close-out Visit

Upon completion of the study, the study monitor or PVS representative and the investigator will conduct the following activities:

- Data clarification and/or resolution
- Accounting, reconciliation and destruction at sites of used and unused vaccines
- Review of site study records for completeness
- Return of all study data to PVS

PVS and Sponsor retain the right to temporarily suspend or prematurely discontinue this study at any time related to safety. If the study is stopped or suspended prematurely, PVS will inform the local principal investigator as well as regulatory authorities about the decision and the reasons for termination or suspension. If such action is taken, all efforts must be made to ensure the safety of the subjects enrolled in the study and the safety of the local community. The principal investigator will assist the Sponsor, Microgen, in informing the responsible IEC and provide the reason for the suspension or termination. In case of premature study or study site closure, the monitor or PVS representative will conduct all activities as indicated above.

10.2 Audits and Inspections

For the purpose of compliance with applicable regulatory guidelines it might be necessary for PVS or national or foreign regulatory authorities to conduct a site audit. This could occur at any time from start to after conclusion of the study.

The principal investigator agrees to allow the auditor direct access to all relevant documents and to allocate his/her time and the time of his/her staff to the auditor to discuss findings and any relevant issues.

National and foreign regulatory authorities may conduct a regulatory inspection of this study. If a regulatory authority requests an inspection, the principal investigator must inform PVS immediately about this request. The principal investigator agrees to allow the inspector(s) direct access to all relevant documents and to allocate his/her time and the time of his/her staff to the inspector(s) to discuss findings and any relevant issues.

10.3 Archiving

In accordance with applicable regulatory requirements, following closure of the study, the investigator/institution will maintain a copy of study documents in a secure and designated location at the study site. Essential documents shall be retained for at least five (5) years after the completion or discontinuation of the study.

11 Statistical Considerations

The data analysis for this study will be conducted primarily by PVS in Seattle, Washington. For the purpose of analysis of the primary safety objectives, the clinical trial database will not be unblinded until the database has been frozen, and the statistical report table shells generated and completed. The Sponsor and PI will participate in the review and approval of the data analyses.

11.1 Study Hypotheses

There are no pre-set statistical hypotheses to be tested in this trial. All objectives are descriptive. As such, no sample size is calculated. Total numbers to be enrolled in the trial are consistent with those of phase 1 vaccine trials.

11.2 Definition of Analysis Sets

All primary safety descriptions and summaries will be performed on an intention-to-treat basis. Supportive per-protocol analyses will also be conducted on all enrolled participants who are randomized, receive two doses of study vaccines and complete the Day 56 visit.

11.3 Analysis Plan

A complete Data Analysis Plan with table shells will be generated and finalized prior to freezing of the database and prior to unblinding of the statistician.

11.3.1 Safety

The safety profile will be parameterized as the proportion of subjects experiencing adverse events (AEs) of the following four categories:

- Immediate reactions occurring within two hours of administration of any dose, measured as observed by study staff or reported by the subject to study staff.
- Adverse events commonly associated with intranasal vaccination (solicited local and systemic reactions) occurring greater than two hours after administration of any dose of study vaccine or placebo through 7 days following any dose, measured as observed by study staff or reported by the subject to study staff.
- All other adverse events (including unsolicited events) occurring during the 7 days following any dose, measured as observed by study staff or reported by the subject to study staff. This includes abnormal laboratory findings from blood and urine specimens collected on Days 7 and 35.
- All serious adverse events (SAEs) occurring within 4 weeks of receipt of any dose, as observed by study staff, reported by the subject to study staff, or noted by the subject on a diary card. This includes abnormal laboratory findings from blood and urine specimens collected on Days 28 (pre-vaccination) and 56.

Counts of all events will be reported and summarized according to event severity, as “any local AE”, or “any systemic AE”, and by relationship to administration of study vaccine or placebo, as deemed by a blinded study clinician. Percentages of subjects experiencing each reaction or event or at least one reaction or event will be calculated along with 95% confidence intervals (CIs) using exact statistical methods.

No multiplicity adjustment to the error rate, alpha, will be made because there are no statistical hypotheses and all analyses will be descriptive.

11.3.2 Immunogenicity

Immune responses will be parameterized as the proportion of subjects with at least a four-fold rise after each dose from baseline or as the mean titer after each dose in any of the following:

- Serum hemagglutination inhibition (HAI) antibodies
- Serum neutralizing antibodies using microneutralization assay
- Serum IgA and IgG by EIA
- Mucosal IgA antibodies in nasal wick specimen

Virus shedding will be parameterized as the proportion of subjects shedding virus (detected by real-time reverse transcriptase polymerase chain reaction (rRTPCR) in nasal or conjunctival swabs or by isolation in chicken embryos) at any time-point. Shedding data will be reported for every subject at every measured time-point.

Percentages of subjects with each immune response will be calculated along with 95% CIs using exact statistical methods. Geometric mean titers (GMTs) along with 95% CIs will also be calculated using the t-test. No multiplicity adjustment to the error rate, alpha, will be made because there are no statistical hypotheses and all analyses of immunogenicity will be descriptive. It is not expected that any subject will have measureable baseline titers to H7. Thus, no screening will be done based on serologic status. However, during analysis, any subject with baseline titer to H7 of >1:20 may be excluded from the immunogenicity analysis or the immunogenicity analysis will be stratified.

Cellular immune responses (cytokine and T-cell) will also be measured using isolated PBMCs tested by flow cytometry and/or ELISPOT assay. These analyses will be exploratory and descriptive only.

12 Source Documents and Source Document Access

Prior to the start of the trial it will be determined and documented which documents or data fields completed by the investigative team will be considered source documents. Source documents for this study may be outpatient charts, inpatient charts, laboratory analysis forms, questionnaires, diary cards, and specimen collection logs. For some data fields, the CRF may be the source document. Data fields on the CRF for which there is a separate primary source document will be carefully completed using that named source document.

Only authorized study staff and representatives of PVS, IEM, Microgen, overseeing ethical review committees and regulatory agencies may have direct access to source documents containing study data. Subject identification will be revealed to authorized representatives of these organizations only when necessary.

13 Quality Control and Quality Assurance

The study will be conducted in accordance with the procedures specified in the protocol and staff will be guided by a study manual of procedures. Study data collection forms will be designed to guide staff on study conduct; forms also will include areas for documenting that activities did, in fact, occur (even if these activities did not require recording of data) and in the appropriate sequence. All study staff must attend mandatory training prior to participant enrollment.

Individual SOPs will be developed and documented for key study procedures and refined/revised as necessary. These SOPs will be included in the study Manual of Procedures (MoP) at the site or in the laboratory.

Site monitoring will be conducted to ensure that human subject protection procedures and study procedures, including study vaccine administration and clinical data and biological specimen collection, are of high quality and that the study is conducted in accordance with the protocol.

After data have been entered in the study database, they will be checked systematically by data management staff according to a pre-specified data validation plan. All listings of the database will be reviewed and discussed for assessment of consistency and medical plausibility. After resolution of all issues, the Statistical Analysis Plan (SAP) will be finalized and the database will be locked after resolution of any remaining queries. An audit trail will be kept of all subsequent changes to the data.

14 Ethics/Protection of Human Subjects

14.1 Ethical Standard

The investigators will ensure that this study is conducted in full conformity with the current revision of the Declaration of Helsinki or with local regulatory requirements, whichever affords the greater protection to the subject.

14.2 Institutional Review Boards and Independent Ethics Committee

PATH maintains an institutional review board (IRB), but for clinical trials PATH has subcontracted its ethical review to the Western Institutional Review Board (WIRB). This study will be reviewed and approved by WIRB prior to study initiation. All amendments will also be approved by WIRB before implementation, as appropriate.

RII also maintains an institutional review board. This study will be reviewed and approved by RII's IRB prior to study initiation. All amendments will also be approved by RII's IRB before implementation, as appropriate.

The protocol and all amendments will also be reviewed and approved by an independent ethics committee (IEC) responsible for trials in RF. This IEC is the RF MoH Ethics Committee.

The PI or designate shall forward copies of all IRB and IEC approvals to PVS prior to the start of the study. The approval letters must identify all documents approved and list the study site, the study investigator, protocol title, version number, and date and, ICF version number and date, and the date of IRB or IEC approval. A list of IRB or IEC members shall be attached to the approval letter.

No deviations from, or changes to, the protocol shall be initiated without prior written IRB and IEC approval of an appropriate amendment, except when necessary to eliminate immediate hazards to the subjects or when the changes involve only logistical or administrative aspects of the study (e.g., change of telephone number, etc.).

The PI will sign all approved versions of the protocol.

14.3 Informed Consent Process

Before inclusion in the trial the volunteer will be provided with oral information and written materials on the objectives and methods for the conduct of the trial, as well as the expected benefits and possible risks associated with participation in the trial. The subject will be informed of the voluntary nature of participation in the trial, and that the volunteer has the right to withdraw from the trial (right to refuse to continue participation in the trial) at any time, and that this refusal will not affect the quality of the volunteer's care. Although the trial volunteer is not obligated to report the reasons for premature termination of participation in the trial, the investigator should attempt to discover the reasons without violating the rights of the trial volunteer. Written informed consent of the subject must be obtained before performing any trial procedures. Subjects will be made aware that authorized representatives of health agencies and the Sponsor and PVS will have access to their confidential medical information for the purposes of monitoring trial conduct or performing audits.

Written informed consent will be obtained from each subject in two-stages. A screening "ICF A" will be signed in duplicate on Day S1. This is because this study requires substantial clinical and laboratory screening of potential subjects prior to admittance to the isolation unit and administration of dose one of study vaccine or placebo. On Day 0, after a subject is fully screened and eligibility is confirmed for admittance in to the isolation unit in order to receive dose one of study vaccine or placebo, the investigator must again review the full trial objectives and methods as well as the expected benefits and possible risks. After this review, a separate vaccine study "ICF B" will be signed in duplicate. Only then may the subject be admitted into the isolation unit for trial procedures.

ICFs will embody the elements of consent as described in the Declaration of Helsinki and the ICH Harmonized Tripartite Guidelines for Good Clinical Practice. Original ICFs must be kept on file by the investigator for possible inspection by regulatory authorities, the Sponsor or PVS. The subject must receive a copy (or second original) of the signed and dated ICF(s), and any subsequent updates or amendments to the ICF. The study monitor shall check the documentation of the individual ICFs during each monitoring visit.

Subjects will be informed that they will be compensated for their time and effort for participation in this trial. Each subject who completes all scheduled visits and procedures will be paid a set amount that is the equivalent of $1000. This amount will be prorated by study visit (see Attachments). Subjects who prematurely withdrawal from the study will be paid a cumulative total for visits and procedures up to the time of premature withdrawal. Subjects in each study sub-cohort will all be paid on one date in one lump sum after the study has been terminated, regardless of whether the subject has fully completed participation in the trial. This payment date will be within one month of the date of termination of each study sub-cohort, as defined in Section 5.5.1.

14.4 Inclusion of Women, Minorities and Children

Enrollment in this study is open to healthy adults of any gender and race or ethnicity. No person may be denied enrollment based on gender or race or ethnicity. However, the investigator should attempt to recruit equal numbers of men and women. The investigator may enroll different races and ethnicities in proportion to their presence in the local population; however, no special recruitment methods will be used to ensure certain levels of participation by any specific minorities residing in the source population. Enrollment in sub-cohort one will be closed when 12 subjects have been screened and determined eligible for admittance to the isolation unit; enrollment in sub-cohort two will be closed when 28 additional subjects have been screened and determined eligible for admittance to the isolation unit two weeks later.

The trial is open to adults 18 through 49 years of age only. Children and adolescents below the age of legal consent in Russia may not be enrolled in this phase 1 trial.

14.5 Insurance

Volunteers who participate in the clinical trial are guaranteed medical insurance during the course of their participation in the trial in accordance with the requirements of law in the RF. The PI must inform the volunteer of the provision of medical insurance for trial related health problems as well as to explain to the subject that the subject must notify the PI prior to the performance of any treatment and concomitant therapy *under this insurance policy* during the trial (except for emergency medical services). The subject will be informed that the insurance company may deny coverage for health problems which are not deemed to be related to participation in the trial.

14.6 Financing

PVS will fully fund this trial. Any financial engagement of PVS or possibly the Sponsor, Microgen, with the clinical study site, Research Institute of Influenza, St. Petersburg, Russia, will be regulated in separate agreements. Financing of the study by PVS will be disclosed to study subjects in the ICFs.

14.7 Subject Confidentiality

14.7.1 Confidentiality of Data

By signing the protocol, the PI agrees that the study protocol, documentation, data, and all other information generated regarding the vaccines will be held in strict confidence. The investigator may divulge such information within regulatory restrictions and ethical considerations only to ethical review committees or similar expert boards or committees, and their affiliated institutions and employees, only under an appropriate understanding of confidentiality with such board or committee, and their affiliated institutions and employees. No information concerning the study or the data may be released to any unauthorized third party without prior written approval of PVS or the Sponsor. Any regulatory agency deemed appropriate, may consult study documents in order to verify CRF data. Investigators will ensure that all employees involved in the study respect the same rules.

Medical information about individual subjects obtained during the course of this study is confidential and may not be disclosed to third parties, except authorized monitors, sponsors, auditors or inspectors. Confidentiality will be ensured by the use of study participant numbers for the identification of each subject; these study participant numbers will also be used for subject data in the subject files at the site and for the CRFs.

14.7.2 Confidentiality of Patient Records

Subject confidentiality is strictly held in trust by the participating investigators, their staff, and the Sponsor and PVS and their agents. This confidentiality is extended to cover testing of biological specimens in addition to the clinical information relating to participating subjects.

Study participants should not be identified by name on any data collection form or on any other documentation sent to PVS and will not be reported by name in any report or publication resulting from data collected in this study.

Documents and data pertaining to the study will be kept in a locked room under the responsibility of the Principal Investigator. PVS will conduct periodic monitoring visits to ensure that the data is safe and stored in this secure place and that only those authorized study staff have access to the data. Only study clinicians and study staff will be granted access to the study data and records. Study data will be kept for 5 years after completion of the study.

The investigators will keep individual results confidential to the extent permitted by law. Information will not be released to anyone other than the participant unless required to do so by law.

14.7.3 Notification of Primary Care Physician

The investigator may release subject clinical and clinical laboratory results data to the subject's primary care physician only if the subject agrees in writing to this action.

14.8 Study Discontinuation

Study discontinuation is not expected to occur. However, if the study is discontinued for safety reasons, subjects will be informed of the reasons for discontinuation and of the implications/potential consequences for the subject.

14.9 Sharing of Study Results with Subjects

All results of clinical laboratory testing should be shared with each subject or made available for review by the subject. Specialty laboratory testing results (influenza virologic and immunologic results generated by IEM) need not be shared with each subject.

When the clinical study report is completed, the investigator may share summary results (absent of all personal identifiers) with subjects. This sharing of summary results may only occur through verbal and visual presentation of summary results. No printed materials containing study summary results may be given to subjects. The investigator may determine the forum for such sharing of study results with subjects, as he desires or is required to do by overseeing ethical review committees.

14.10 Future Use of Stored Specimens

Biological specimens will be maintained until the end of the study, as appropriate. This is to allow time for all study-related testing. The specimens will be maintained in the laboratories of RII and IEM. Specimens will not be labeled with any personal-identifying information. Following completion of the study, all specimens will be destroyed unless written informed consent has been obtained to store the subject's specimens for up to 20 years for future use. Stored specimens may become an extremely valuable resource for future development of improved influenza vaccines. Written informed consent for such long-term storage of any subject's specimens will be requested and obtained after screening, at the time that the subject provides written informed consent for participation in the vaccine study. If the subject provides consent for long-term storage, only specimens collected after screening may be stored for future use. However, these specimens will not be used for unrelated, future studies without appropriate ethical review and approval and consent from study subjects, if deemed necessary by any ethical review committee.

15 Data Handling and Record Keeping

15.1 Data Management Responsibilities

The investigator is responsible to ensure the accuracy, completeness, legibility, and timeliness of the data reported. Data collection is the responsibility of the clinical study staff at the site under the supervision of the primary investigator.

AEs must be graded, assessed for severity and causality, and reviewed by the primary investigator or designee.

PVS will hire and oversee a data management contractor who will conduct data management, entry and quality review. Copies of the CRF will be sent to the data management contractor. Analysis and reporting of the created study database will be done by the PVS-designated trial statistician.

15.2 Data Capture Methods

All the information required by the study protocol must be recorded on the CRF provided by PVS. All data must be entered legibly, as described in any *CRF Completion Guidelines*. An explanation must be provided for any missing data.

All source documents and CRFs should be completed in a neat, legible manner to ensure accurate interpretation of data. Black ink is required to ensure clarity of reproduced copies. When making changes or corrections, cross out the original entry with a single line, and initial and date the change. DO NOT ERASE, OVERWRITE, OR USE CORRECTION FLUID OR TAPE ON THE ORIGINAL. All source documents and laboratory reports must be reviewed by the clinical and laboratory teams, who will ensure that they are accurate and complete.

The investigator must sign and date each CRF, attesting to his responsibility for the quality of all data recorded and that the data represent a complete and accurate record of each subject's participation in the study.

Clinical safety data and virologic and immunologic laboratory data will be entered onto study CRFs from laboratory report forms. Visit dates and laboratory procedure dates will be recorded on all forms. All participants will be assigned a unique study participant number at study enrollment – this study participant number will be included on all forms and in the trial database and will serve to link study data to specific individuals. CRFs will be entered, verified for accuracy, linked by study participant number, and managed using database management software.

15.3 Types of Data

Data for this study will include biographical, medical history, clinical (signs, symptoms, prescription and non-prescription medical treatments) safety data, laboratory safety (biochemical and hematologic) data, and virologic and immunologic laboratory data.

15.4 Timing/Reports

The Clinical Study Report is expected to be completed within six months of completion of the last CRF (last laboratory data or last follow-up data for an SAE entered into the CRF).

15.5 Study Records Retention

It is planned that the study data will be kept for 5 years after completion of the study. No records will be destroyed without the written consent of PVS. It is the responsibility of PVS to inform the PI when these documents no longer need to be retained.

15.6 Protocol Deviations

A protocol deviation is any noncompliance with the clinical study protocol, GCP, or the site MoP. The noncompliance may be either on the part of the subject, the investigator, or the study site staff. As a result of any deviations, corrective actions are to be developed by the site and implemented promptly. Examples of protocol deviations include the following:

- Irregular or improper implementation of the trial product;
- Unnecessary repeated performance of any planned procedures or tests;
- Changing the interval between two visits or procedures more than allowed in the protocol
- Incorrect or careless documentation of study procedures on primary documents, required logs, or the CRF.
- Premature withdrawal of a subject for any reason.

Trial procedures shall not be changed without the consent of PVS and the Sponsor. Insignificant violations of the protocol will be examined on an individual basis taking into account recorded information for the reason(s) that the deviation occurred.

It is the responsibility of the site to use continuous vigilance to identify and report deviations to PVS in a timely manner after identification. Reports of protocol deviations must be sent to WIRB, the RII IRB, and the IEC of RF MoH, as required per their respective guidelines. The PI and his staff are responsible for knowing and adhering to their IRB's requirements and those of the IEC in Russia. PVS will assist the investigator in all reporting to WIRB or file reports on behalf of the investigator.

16 Final Report on the Trial and Publication Policy

A Clinical Study Report comprised of text and results tables reflecting all safety and immunogenicity data will be generated. The CRFs of all subjects will be attached to the CSR. The CRS will be reviewed, approved and signed by the Principal Investigator. The CSR will be compliant with ICH guidelines and composed in accordance with the “Rules of Clinical Practice in the Russian Federation.” Where any minor differences exist between these rules, Russian standards will take precedence.

All data, documents, any recordings and information transferred by the Sponsor to any contractor or obtained or prepared by any contractor, his consultants or persons associated by contractual relationships with any contractor during the trials, belong to the Sponsor.

All confidential information communicated to the Principal Investigator by PVS, IEM or Microgen shall be kept strictly confidential by him/her or any other person connected with the study and shall not be disclosed, either orally or in written form, by him/her or such person to any third party without prior written consent of the organization of which the information is the exclusive property.

Following completion of the CRS, the investigators, working with PVS, IEM and Microgen representatives, are expected to publish the results of this research in peer-reviewed scientific journal(s). In no way may the Sponsor prohibit the public dissemination of the results of this trial; details of the publication plan are specified in the Agreement between Microgen, PVS, IEM, and RII.

The International Committee of Medical Journal Editors (ICMJE) member journals have adopted a trials-registration policy as a condition for publication. This policy requires that all clinical trials be registered in a public trials registry. It will be the responsibility of PVS representatives to register this trial in an acceptable registry. ICMJE authorship criteria will be strictly followed for publication of any manuscript(s) arising from this trial.

17 Literature References

[1] Kieny, MP and Fukuda K. The pandemic influenza vaccine challenge. Vaccine. 2008; 26S:D3-D4.

[2] Sambhara S and Poland GA. H5N1 avian influenza: preventive and therapeutic strategies against a pandemic. Annu Rev Med. 2010; 61:187-198.

[3] Osterhaus ADME and Poland GA. Vaccines against seasonal and avian influenza: recent advances. Vaccine. 2008; 26S:D1-D2.

[4] Regulatory Preparedness for Human Pandemic Influenza Vaccines. In WHO Expert Committee on Biological Standardization. Geneva, World Health Organization, 2007. http://www.who.int/biologicals/publications/trs/areas/vaccines/influenza/Human_pandemic_Influenza_Vaccines_BS2074_01Feb08.pdf

[5] WHO Department of Communicable Disease. WHO global influenza preparedness plan: the role of WHO and recommendations for national measures before and during pandemics. Geneva, World Health Organization, 2005. http://www.who.int/csr/resources/publications/influenza/WHO_CDS_CSR_GIP_2005_5.pdf

[6] National Institute of Allergy and Infectious Diseases (NIAID). Meeting summary for HHS workshop on pandemic influenza vaccines. "Development of a Clinical Trial Plan for Pandemic Influenza Vaccines." http://www.niaid.nih.gov/about/organization/dmid/Documents/pansummary.pdf NIAID website, accessed 08 July 2011.

[7] WHO Immunization, Vaccines and Biologicals. Global action pandemic influenza action plan to increase vaccine supply. Geneva, World Health Organization, 2006. http://whqlibdoc.who.int/hq/2006/WHO_IVB_06.13_eng.pdf

[8] Rudenko L, Desheva J, Korovkin S, Mironov A, Rekstin A, Grigorieva E, et al. Safety and immunogenicity of live attenuated influenza reassortant H5 vaccine (phase I-II clinical trials). Influenza and other Respiratory Viruses. 2008; 2:203-209.

[9] Global pandemic influenza action plan to increase vaccine supply: progress report 2008. In WHO Immunization, Vaccines and Biologicals. Geneva, World Health Organization, 2009. http://www.who.int/vaccine_research/Global_Pandemic_Influenza.pdf

[10] Petukhova G, Naikhin A, Chirkova T, Donina S, Korenkov D, and Rudenko L. Comparative studies of local antibody and cellular immune responses to influenza infection and vaccination with live attenuated reassortant influenza vaccine (LAIV) utilising a mouse nasal-associated lymphoid tissue (NALT) separation method. Vaccine. 2009;27:2580–2587.

[11] Karron RA, Talaat K, Luke C, Callahan K, Thumar B, DiLorenzo S, et al. Evaluation of two live attenuated cold adapted H5N1 influenza virus vaccines in healthy adults. Vaccine. 2009; 27:4953-4960.

[12] Talaat KR, Karron RA, Luke CJ, Thumar B, McMahon BA, Chen GL, et al. An open label Phase I trial of a live attenuated H6N1 influenza virus vaccine in healthy adults. Vaccine. 2011;29:3144-3148.

[13] Talaat KR, Karron RA, Callahan KA, Luke CJ, DiLorenzo SC, Chen GL, et al. A live attenuated H7N3 influenza virus vaccine is well tolerated and immunogenic in a Phase I trial in health adults. Vaccine. 2009;28:3744-3753.

[14] Karron RA, Callahan K, Luke C, Thumar B, McAuliffe J, Schappell E, et al. A live attenuated H9N2 influenza vaccine is well tolerated and immunogenic in healthy adults. Journal of Infectious Diseases. 2009; 199:711-716.

[15] David Wood. WHO meeting on development and evaluation of influenza pandemic vaccines. “Regulatory issues – comments from WHO.” http://www.who.int/vaccine_research/diseases/influenza/Wood.pdf WHO website, accessed 09 June 2011.

18 Attachments

Attachment 1. Example schedule of events for subjects on Day 28.

Example schedule of events for subjects on Day 28.

Study Procedure	08:00	09:00	10:00	11:00	12:00	13:00	14:00	15:00	16:00	17:00	18:00	19:00	20:00	21:00	22:00	23:00
Subjects present to study center	✓															
Review diary cards/interim medical history		✓			l						d		s			l
Collect blood specimens for CMP and CBC			✓		u						i		u			i
Collect urine sample for testing			✓		n						n		p			g
Perform physical examination				✓	c						n		p			h
Perform ENT examination				✓	h						e		e			t
Confirm eligibility						✓					r		r			s
Re-admit subject to isolation unit						✓										
Collect nasal swab specimen						✓										o
Collect nasal wick specimen (for IgA testing)							✓									u
Collect blood specimens for immunogenicity							✓									t
Collect blood for cellular immunity studies							✓									
Administer study vaccine or placebo								✓								
Monitor for immediate reactions								✓								
Record AEs/SAEs								✓	✓	✓	✓	✓	✓	✓	✓	
Collect nasal swab specimen														✓		
