# Supplementary material for: Assessment of Human Immune Responses to H7 Avian Influenza Virus of Pandemic Potential: Results from a Placebo–Controlled, Randomized Double–Blind Phase I Study of Live Attenuated H7N3 Influenza Vaccine
Source: PLoS One. 2014 Feb 12;9(2):e87962. doi: 10.1371/journal.pone.0087962 (PMC3922724; doi:10.1371/journal.pone.0087962)
Supplement: Randomization S1 — Randomization Plan for PVS Protocol LAIV-H7N3-01. (PDF) [file pone.0087962.s004.pdf]

Randomization Plan for PVS Protocol LAIV-H7N3-01

1. Introduction

The study will use a "random permuted block" design to assure more equal spacing of the two treatments in the allocation sequence. This will also assure a 3:1 vaccine and placebo balance for each study cohort, should we need to stop the trial early for any reason before all subjects are enrolled and vaccinated. Since we only have two treatments, the block size should be a whole number multiple of two and the sum of the treatment ratio (3+1=4) to assure balance across the treatment groups . The advantage to having larger block sizes is that it reduces "predictability" and hence reduces the possible introduction of bias into the study. The smaller the block size, the more likely some study administrator, researcher or physician will be able to figure out the distribution of treatments within a block. The larger the block size, the less likely that someone is able to figure out the treatment allocation for everyone in the block based on some "partial information", i.e. what the treatment allocation was for the first or second individual within a block.

We will randomize treatment in blocks of four, which is the smallest possible block size. This is reasonable for a small trial such as this with only 40 participants. For a block size of four, there are four possible permutations (of sequential order of allocation):

1. VACCINE VACCINE VACCINE placebo
2. VACCINE VACCINE placebo VACCINE
3. VACCINE placebo VACCINE VACCINE
4. placebo VACCINE VACCINE VACCINE

Since the unit of randomization is the individual, this should not alter statistical efficiency for a trial of n=40 in any substantial way, although there are no pre-stated statistical hypotheses to be tested. Using such a block size will help reduce the unlikely possibility that blinded investigators (at RII, PATH, or PSI staff) could easily become unblinded. Since the blinded staff will only know that there is an ordered list of subjects allocation codes from 101 to 140, if staff find out that one person received vaccine and know who is in a particular block of four, they still will be unable to know which of the above four permutations of vaccine and placebo were assigned to the others in the block. If they find out that one person received placebo, however, they could become unblinded to the other three persons in the block. This is one reason that PATH will not communicate the block size to either RII or PSI blinded staff.

2. Responsibility for Generation of the Allocation Sequence

The allocation (randomization) sequence for vaccine and placebo will be generated by a PATH staff scientist, Dr. Justin Ortiz. Dr. Ortiz will make final pdfs of the allocation sequence as follows: one for study cohort 1 (n=12), dose 1 and one for study cohort 1, dose 2; one for study cohort 2 (n=28), dose 1 and one for study cohort 2, dose 2. The four allocation sequence pdfs will be verified by another PATH staff scientist (Kristen Lewis). Neither Dr. Ortiz nor Ms. Lewis will have any involvement with any trial follow-up, monitoring or analysis. One original of the allocation sequences will be delivered by blinded PATH clinical trial lead, Dr. John Victor, and one initialed, scanned and printed copy will be stored at the PATH office in Seattle in a secure location. In Russia, only unblinded RII and PSI staff will be permitted to access the allocation sequences; in Seattle, only limited and designated staff not involved in study execution and analysis will be permitted access; before being permitted access, any other PATH staff (besides Dr. Ortiz and Ms. Lewis) must be first designated in writing and approved by PATH clinical trial lead, Dr. John Victor.

3. Generation of the Allocation Sequence

The following mechanics will be used by Dr. Ortiz for generating codes:

1. Dr. Ortiz will use the generator at randomization.com.
2. Dr. Ortiz will click the link for <first (and original) generator>: http://www.jerrydallal.com/random/random_block_size.htm
3. Dr. Ortiz will enter “Vaccine”, “Vaccine”, “Vaccine” and “Placebo” in the first four blank white boxes.
4. Dr. Ortiz will enter “4” and “10” in the two blank white boxes for the first line of “Number of subjects per block/number of blocks”, respectively. Subsequent lines will be left blank. Initial subject ID number should be entered as “101”. This will generate a randomization list of 40 vaccine and placebo allocations with 40 unique allocation codes, 101 through 140. See example screenshot below.
5. Dr. Ortiz will click the “Generate Plan” button.

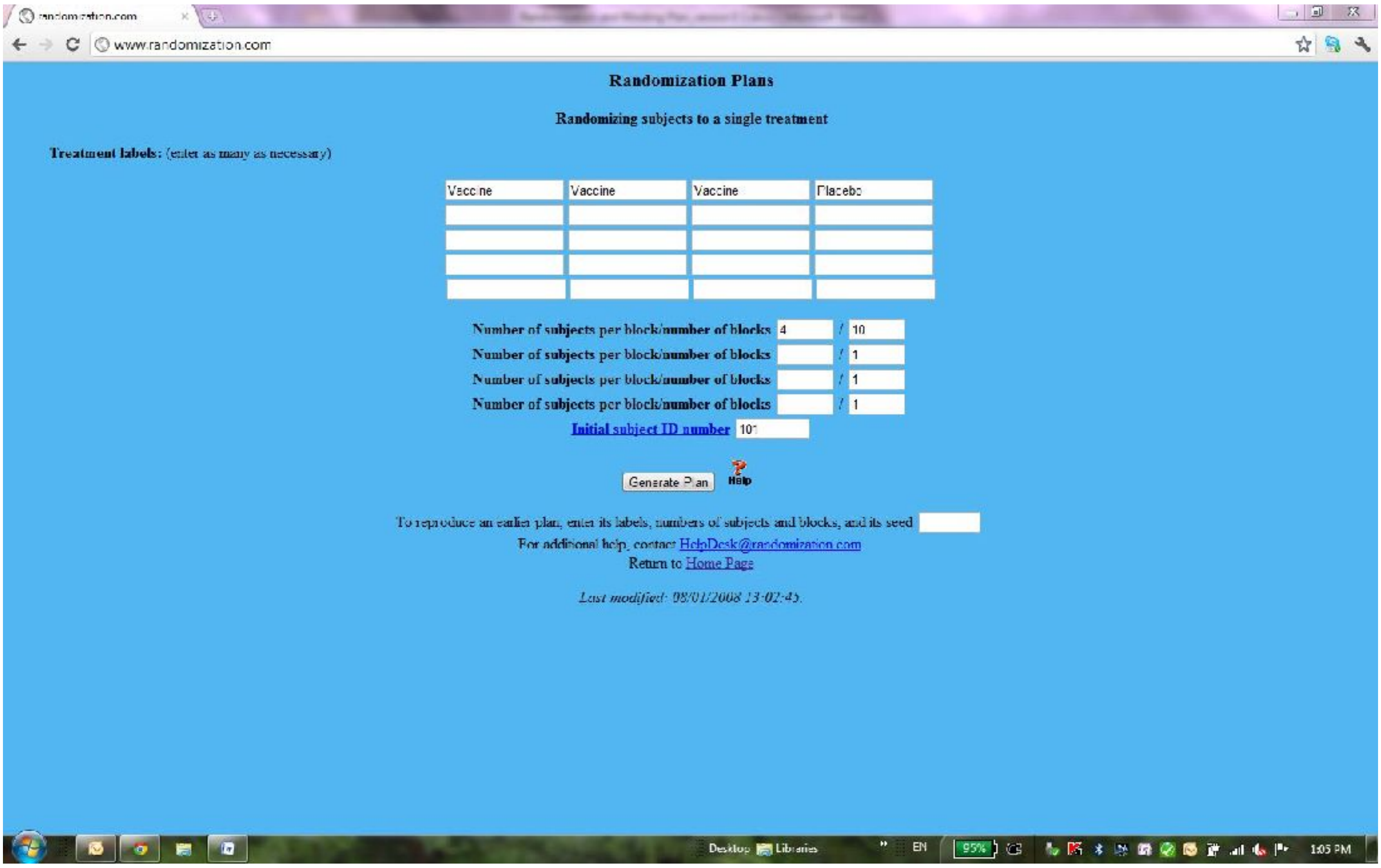

The resulting allocation sequence will be generated (see example screenshot below). This web page will be printed to a pdf, to be named “Allocation Sequence_LAIV-H7N3-01.pdf”, assuring that all 40 allocations are clearly printed and that the footnote to the sequence, which shows the “seed” to reproduce the plan and the date and time that the plan was generated, is clearly visible in the saved pdf.

(Each run of a randomization can later be re-generated by entering the "seed" number into the online program. Dr. Ortiz and Ms. Lewis will not reveal this seed to anyone else at PATH office, unless that person has been designated in writing to view the sequence and know the seed which could be used to re-generate it.)

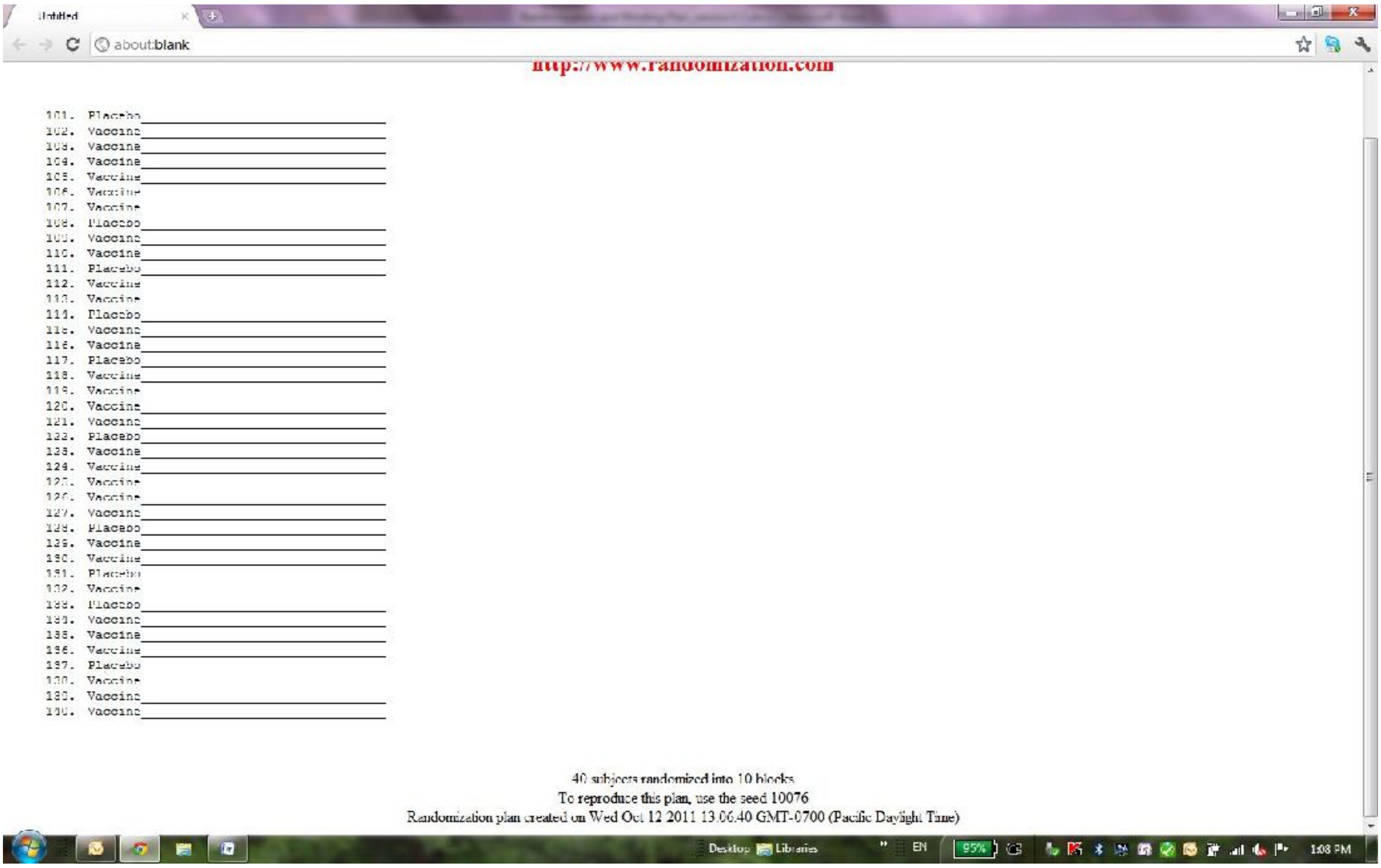

4. Soft Printing of the Allocation Sequence

Using the pdf of the web page with the output of the randomization generator and the Excel file "Allocation Numbers_LAIV-H7N3-01.xlsx" (attached) and in column D, Dr. Ortiz will adjust the allocations of LAIV or placebo by copying and pasting in Excel until the list of allocations and allocation numbers matches that on the web page pdf. The file will then be saved to his hard drive.

Dr. Ortiz will then copy and paste the list identically from the primary file into each of the following four Excel files, which are labeled and set to print the list for cohort 1 or cohort 2 and for dose 1 or dose 2.

Each of these Excel files should then be printed to an identically named pdf file. (Note, only the first 12 allocations OR the following 28 allocations should print on any one sheet so that documents are generated for cohort 1 or cohort 2—and dose 1 or dose 2—on each document.)

5. Physical Printing of the Allocation Sequences

Dr. Ortiz will then verify the contents of each of the four Allocation Number pdfs against the Allocation Sequence pdf print-out of the original **web page**. After his verification, he will send to Ms. Lewis the 5 pdfs (Allocation Sequence and Allocation Numbers), and Ms. Lewis will verify the Allocation Number pdfs against the pdf of the print-out of the original **web page**.

When Dr. Ortiz and Ms. Lewis have concurred on the veracity of the four Allocation Number pdfs, Dr. Ortiz will print one copy of each of the four Allocation Number pdfs, and Dr. Ortiz and Ms. Lewis will each initial, using blue ink, every printed page at the bottom of the page in the space provided.

Dr. Ortiz will then scan the four printed and signed Allocation Number documents together into one non-manipulatable pdf (meaning, scan it as an image and not as computer readable text), ordering the four signed documents as follows: 1) cohort 1, dose 1; 2) cohort 2, dose 1; 3) cohort 1, dose 2, and 4) cohort 2, dose 2. This file will be named "FINAL Allocation Sequence.pdf" and will be saved securely on the computer with the other generated files. This file should then be printed to hardcopy.

Besides the four printed Allocation Number documents and the Final Allocation Sequence document, any other paper copies of documents printed during the verification process must be destroyed using the secure bins at PATH.

6. Sealing of the Allocation Sequence

After printing and signing of the verified four Allocation Number pdfs, Dr. Ortiz will place these documents between several blank sheets of paper and place them into one envelope which will be labeled "LAIV-H7N3 Allocation Sequence – Originals". **This envelope will be sealed and signed and dated by Dr. Ortiz across the flap.** Dr. Ortiz will maintain this envelop in a secure location at the PATH office in Seattle.

The printed single document (print of "FINAL Allocation Sequence.pdf") which is the scanned copy of the printed, signed and scanned four Allocation Number pdfs, should be also placed between several blank sheets of paper and placed into an envelope which will be labeled "LAIV-H7N3 Allocation Sequence _TO RUSSIA". **This envelope will also be sealed and signed and dated by Dr. Ortiz across the flap.** This envelop will be given to Dr. Victor, blinded PATH scientist, who will hand-carry the envelop to Russia for use at the field site.

7. Maintenance of the Allocation Sequences at PATH

Dr. Ortiz will maintain all electronic documents generated as part of the randomization in a secure location not accessible to other PATH staff involved in the trial. Dr. Ortiz should maintain rapid access to this information at all times in case that any unblinding is required during the course of the trial. In case that Dr. Ortiz is on travel or leave and unable to access the documents, Ms. Lewis will be his backup. In case that Ms. Lewis is also on travel or leave, Ms. Kristin Bedell will be a 3rd backup. Further plans will be made depending on the exact timing of dosing in the trial when trial initiation dates approach. Dr. Ortiz should instruct Ms. Lewis and Ms. Bedell on how to access the documents in case of such emergency or request from Dr. Victor. Once opened, the "LAIV-H7N3 Allocation Sequence – Originals" documents should be resealed with the person's signature and date written across the seal and again stored securely.

The allocation sequences will not be opened or linked to the dataset until after the dataset is declared locked and the key tables are generated. In case of urgency, treatment allocation to a subject may be communicated to the investigator only if that information is deemed necessary to properly treat the subject for the SAE. Treatment allocation may also be communicated to the trial Safety Monitoring Committee only if they request this information. Dr. Ortiz will directly communicate this information to the required person at the advice of Dr. Victor. Dr. Ortiz will document all communications in writing or email, even if primary communication is via telephone. If masking is broken, it must be properly documented by all parties. Under no circumstances will treatment allocation be communicated by Dr. Ortiz to Dr. Victor.
