# Supplementary material for: Assessment of Human Immune Responses to H7 Avian Influenza Virus of Pandemic Potential: Results from a Placebo–Controlled, Randomized Double–Blind Phase I Study of Live Attenuated H7N3 Influenza Vaccine
Source: PLoS One. 2014 Feb 12;9(2):e87962. doi: 10.1371/journal.pone.0087962 (PMC3922724; doi:10.1371/journal.pone.0087962)
Supplement: Supplement S1 — Representative results of partial sequencing of four H7N3 LAIV clinical isolates from universal primers designed by Hoffmann et al [7] . (PDF) [file pone.0087962.s005.pdf]

PB2 gene

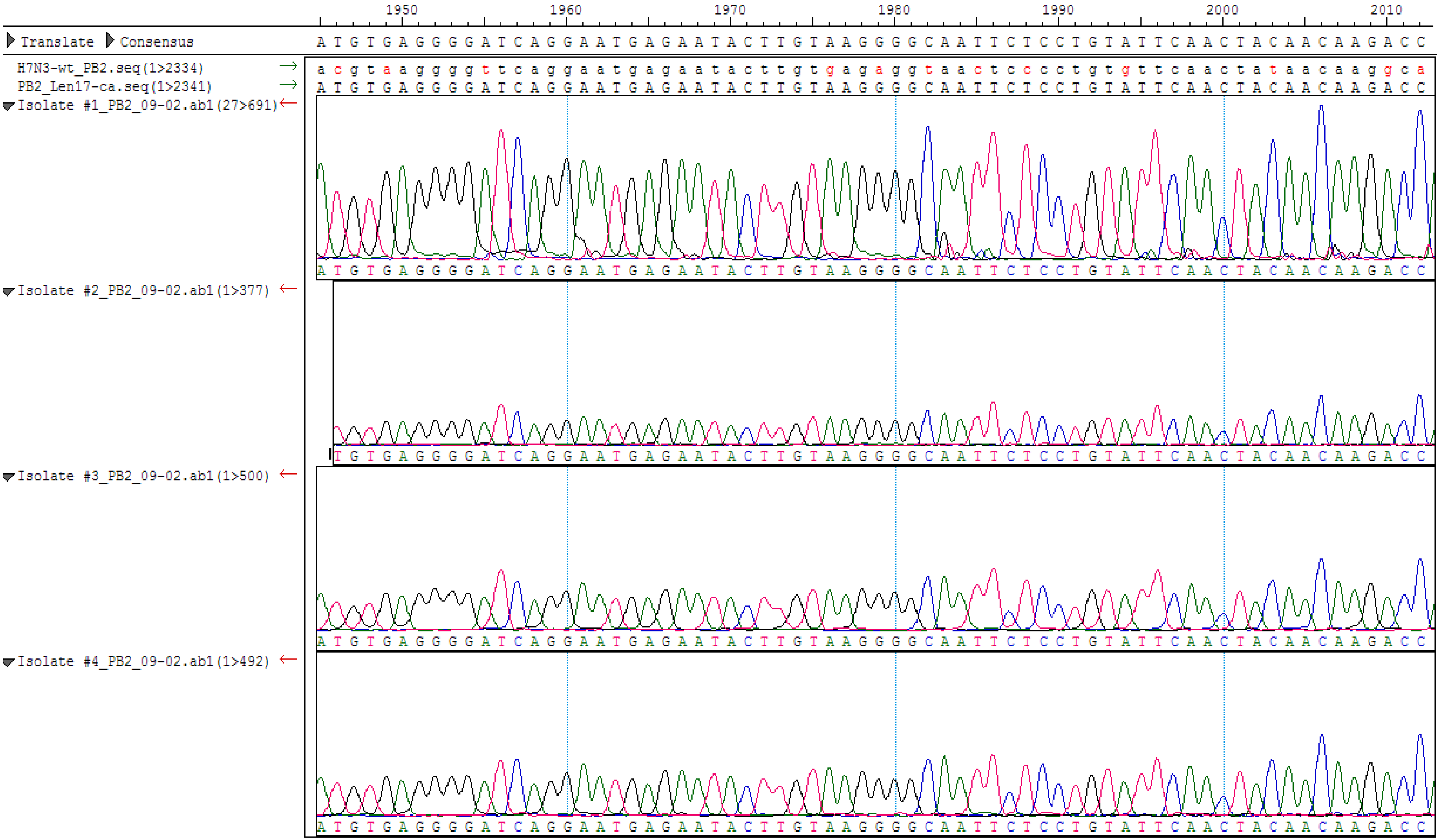

H7N3-wt: A/mallard/Netherlands /12/2000 (H7N3) wild-type virus;
Len17-ca: A/Leningrad/134/17/57 (H2N2) master donor virus;

PB1 gene

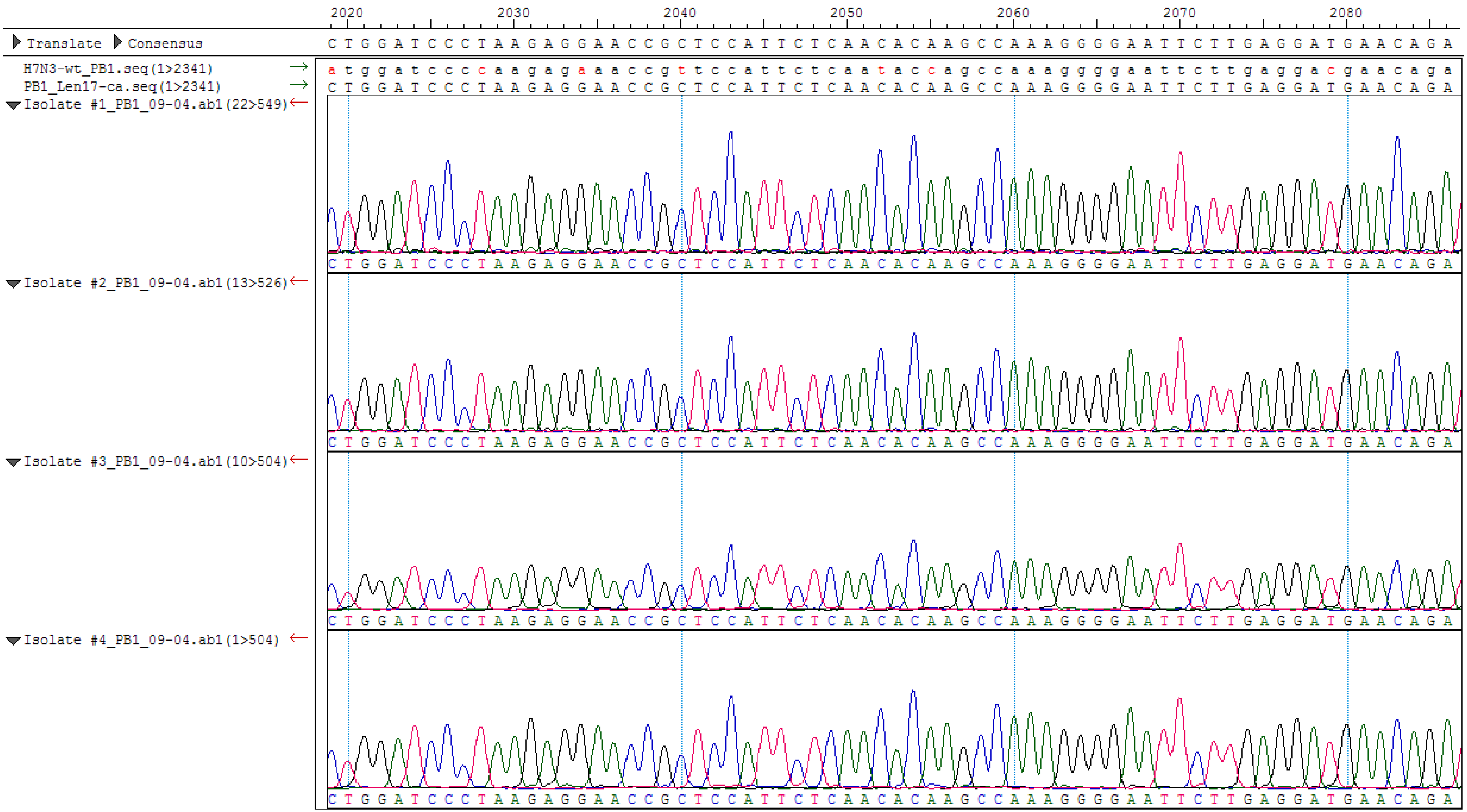

H7N3-wt: A/mallard/Netherlands /12/2000 (H7N3) wild-type virus;
Len17-ca: A/Leningrad/134/17/57 (H2N2) master donor virus;

PA gene

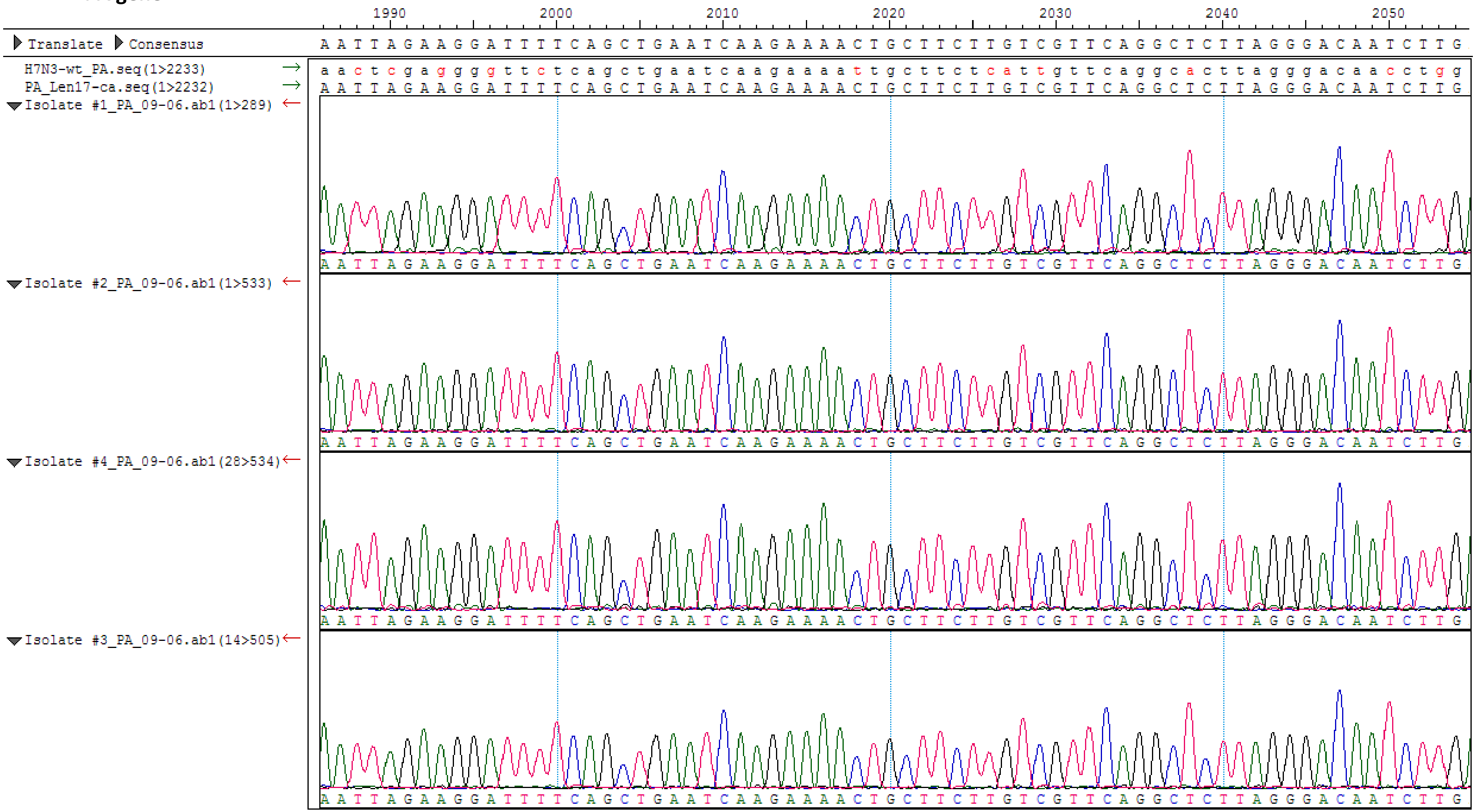

H7N3-wt: A/mallard/Netherlands /12/2000 (H7N3) wild-type virus;
Len17-ca: A/Leningrad/134/17/57 (H2N2) master donor virus;

HA gene

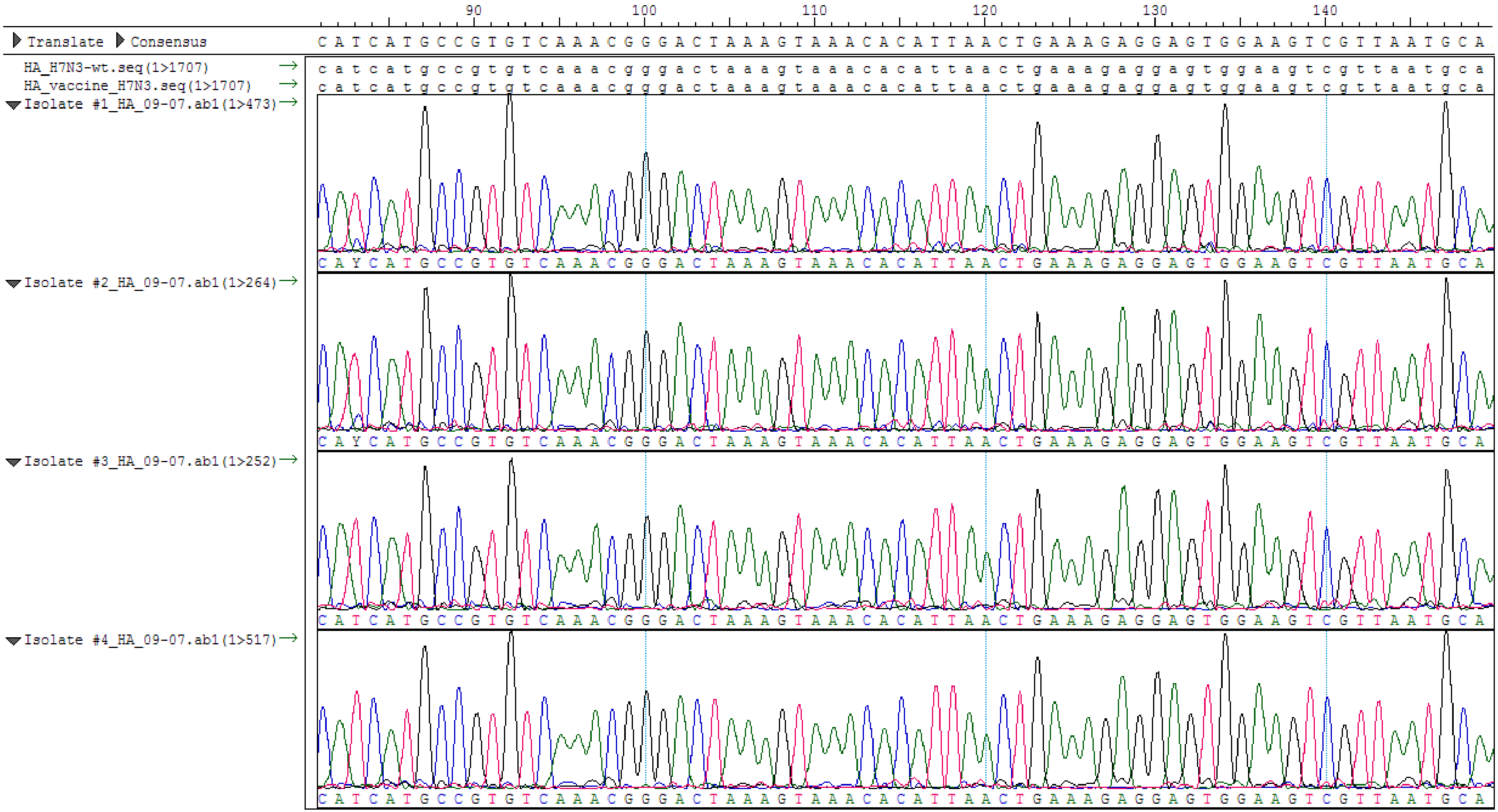

H7N3-wt: A/mallard/Netherlands /12/2000 (H7N3) wild-type virus;
Vaccine_H7N3: A/17/mallard/Netherlands/00/95 (H7N3) LAIV

NP gene

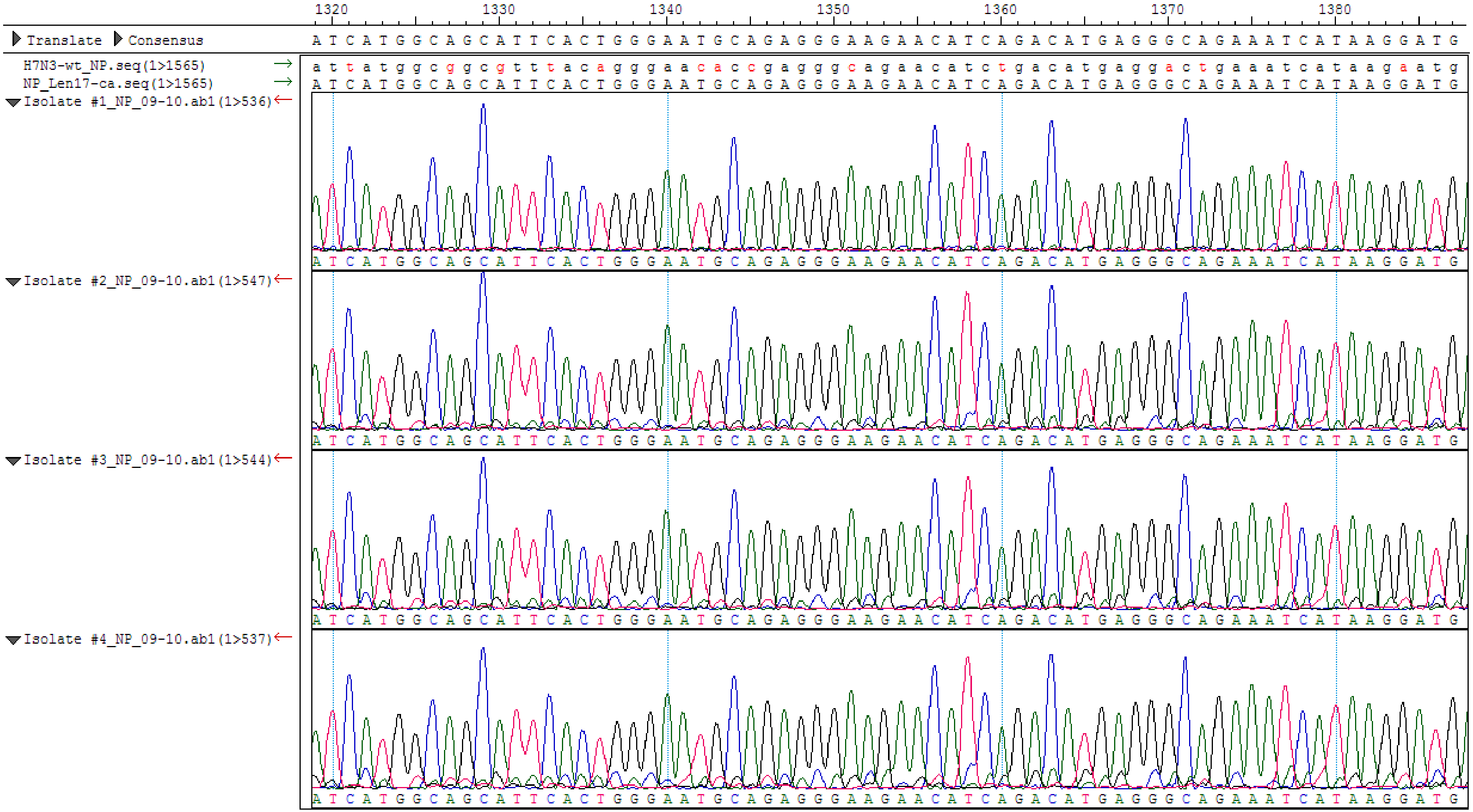

H7N3-wt: A/mallard/Netherlands /12/2000 (H7N3) wild-type virus;
Len17-ca: A/Leningrad/134/17/57 (H2N2) master donor virus;

NA gene

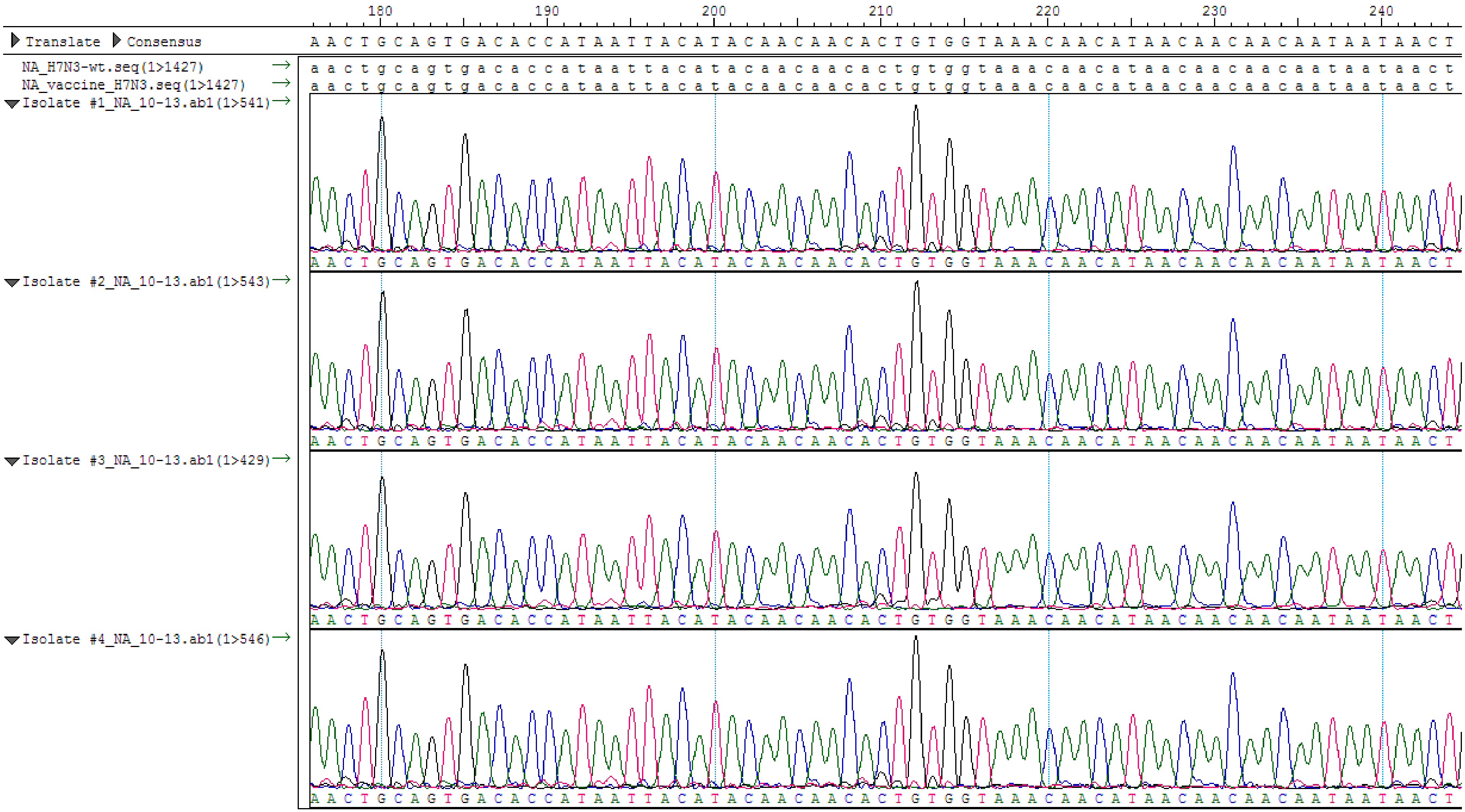

H7N3-wt: A/mallard/Netherlands /12/2000 (H7N3) wild-type virus;
Vaccine_H7N3: A/17/mallard/Netherlands/00/95 (H7N3) LAIV

M gene

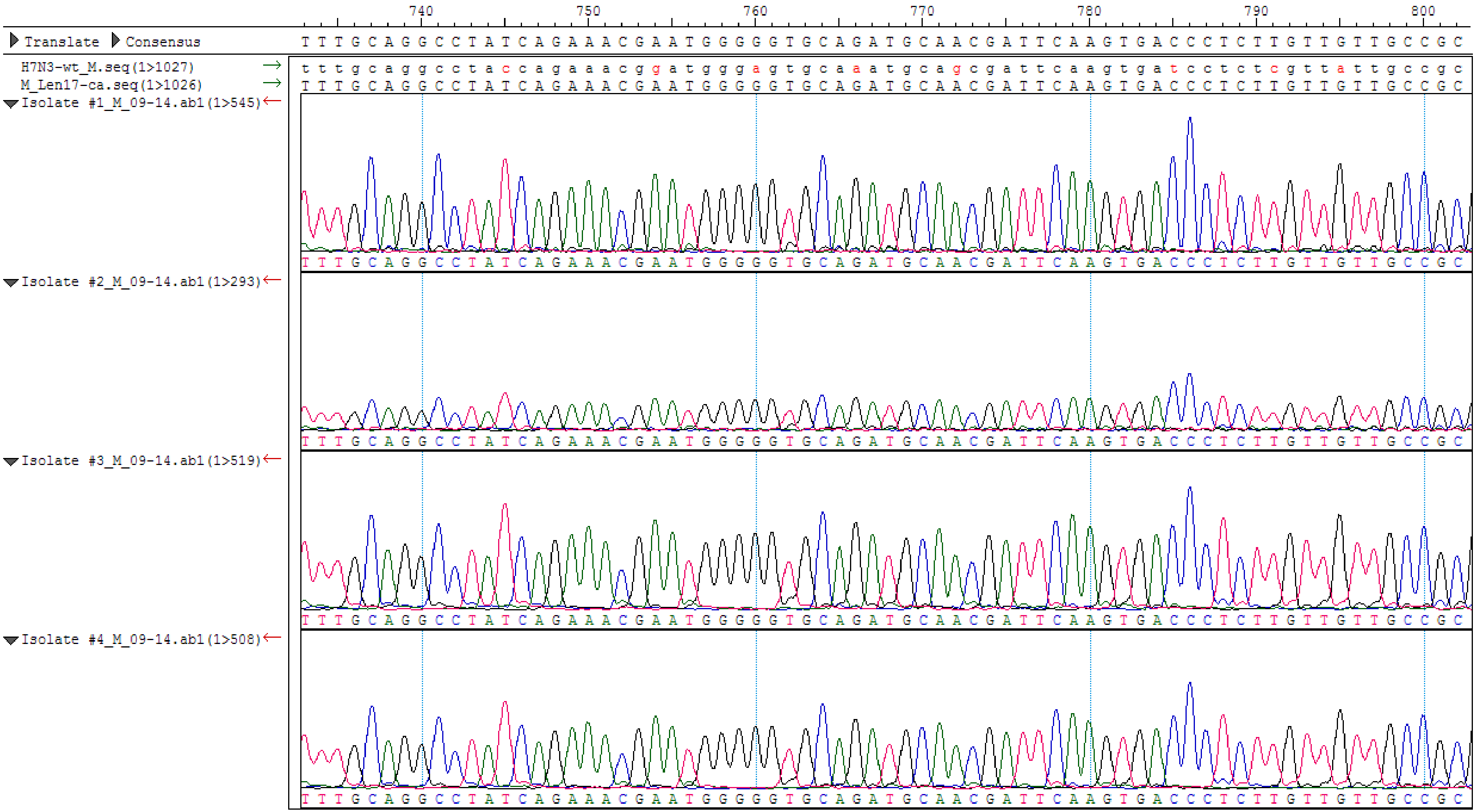

H7N3-wt: A/mallard/Netherlands /12/2000 (H7N3) wild-type virus;
Len17-ca: A/Leningrad/134/17/57 (H2N2) master donor virus;

NS gene

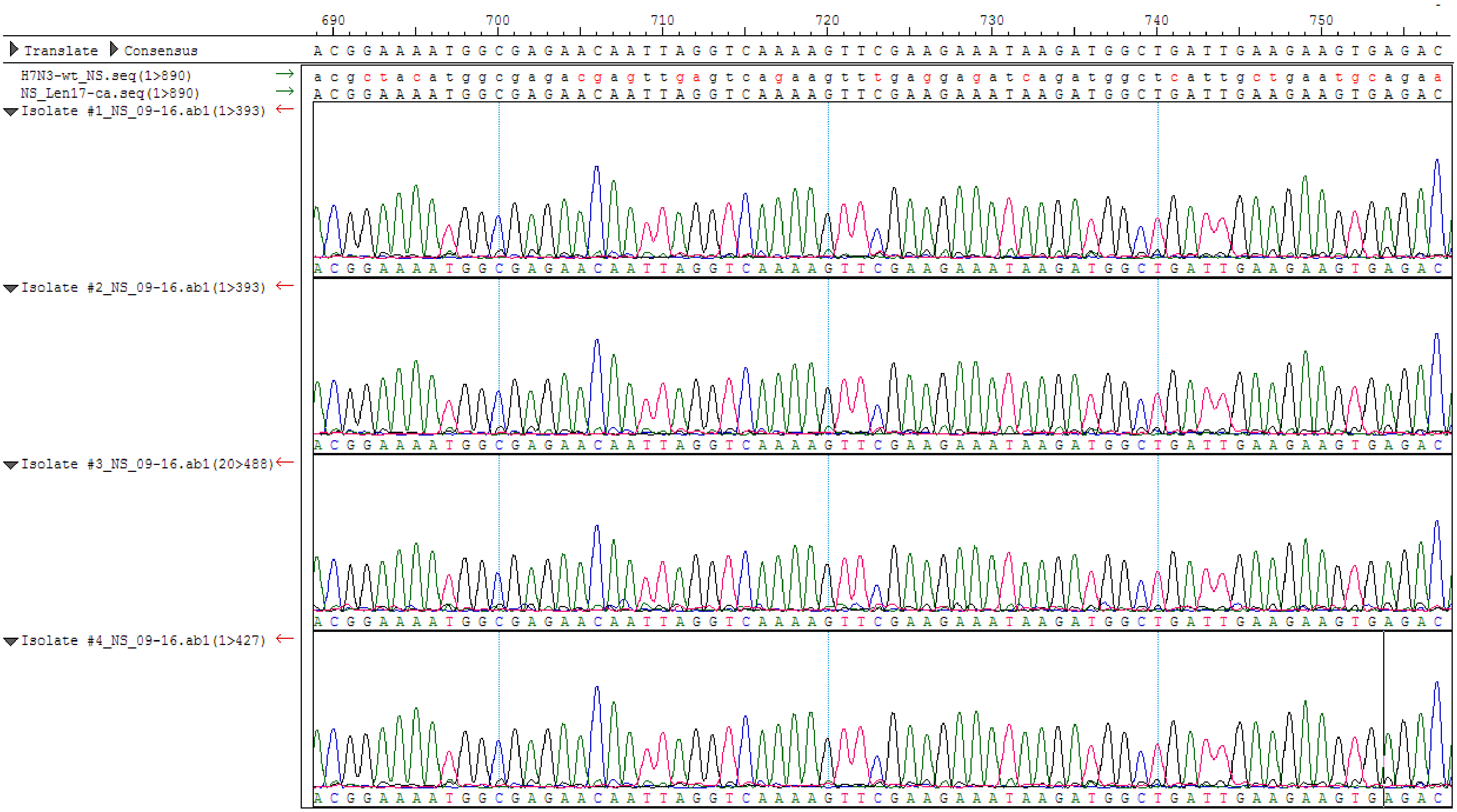

H7N3-wt: A/mallard/Netherlands /12/2000 (H7N3) wild-type virus;
Len17-ca: A/Leningrad/134/17/57 (H2N2) master donor virus;
