# Supplementary material for: Assessment of Human Immune Responses to H7 Avian Influenza Virus of Pandemic Potential: Results from a Placebo–Controlled, Randomized Double–Blind Phase I Study of Live Attenuated H7N3 Influenza Vaccine
Source: PLoS One. 2014 Feb 12;9(2):e87962. doi: 10.1371/journal.pone.0087962 (PMC3922724; doi:10.1371/journal.pone.0087962)
Supplement: Supplement S2 — Results of detection of attenuating mutations in internal genes of four H7N3 LAIV clinical isolates using partial sequencing. (PDF) [file pone.0087962.s006.pdf]

Mutation in PB2 gene: G-1459-T (Val-478-Leu)

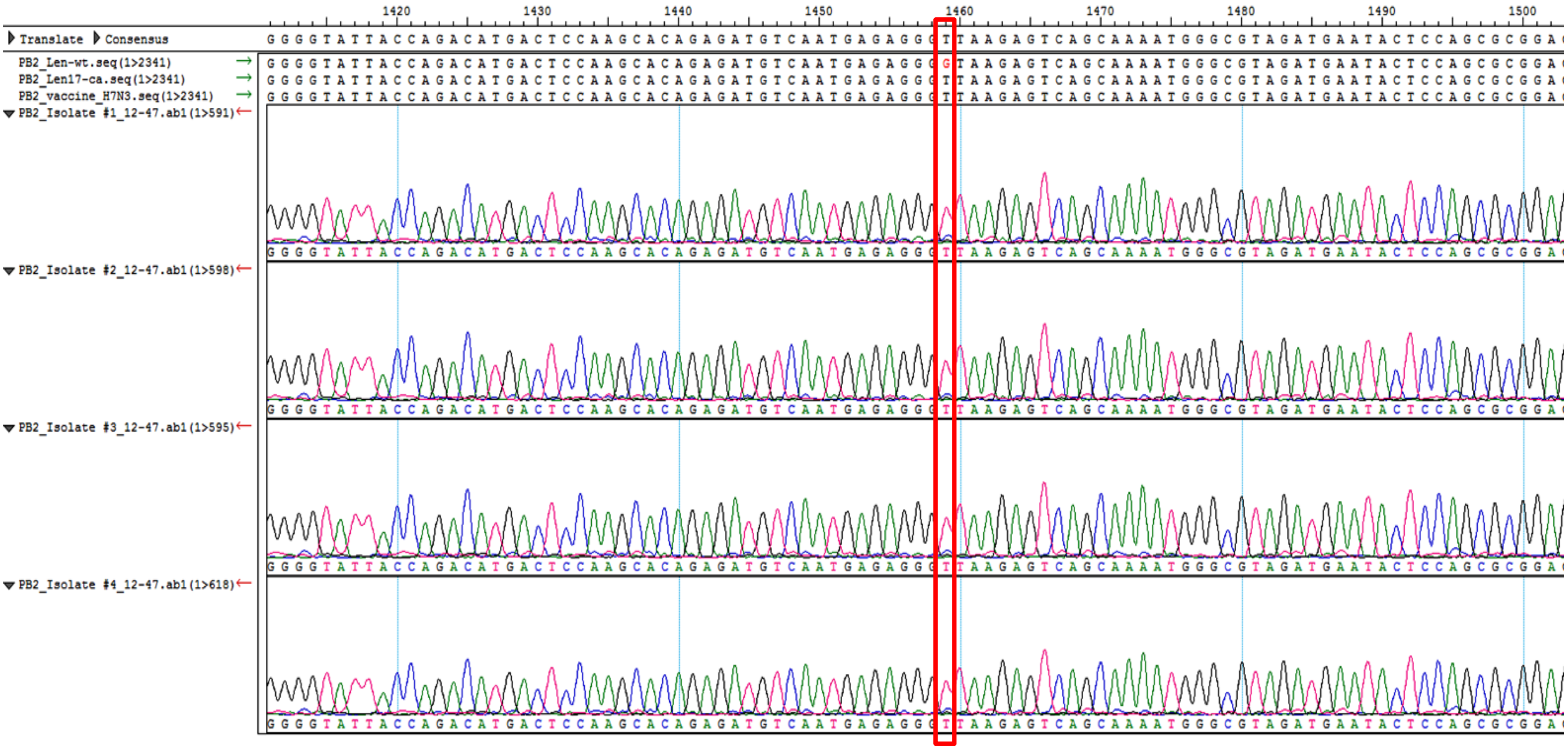

Len-wt: A/Leningrad/134/57 (H2N2) wild-type virus;
Len17-ca: A/Leningrad/134/17/57 (H2N2) master donor virus;
Vaccine_H7N3: A/17/mallard/Netherlands/00/95 (H7N3) LAIV

Mutation in PB1 gene: G-819-T (Lys-265-Asn)

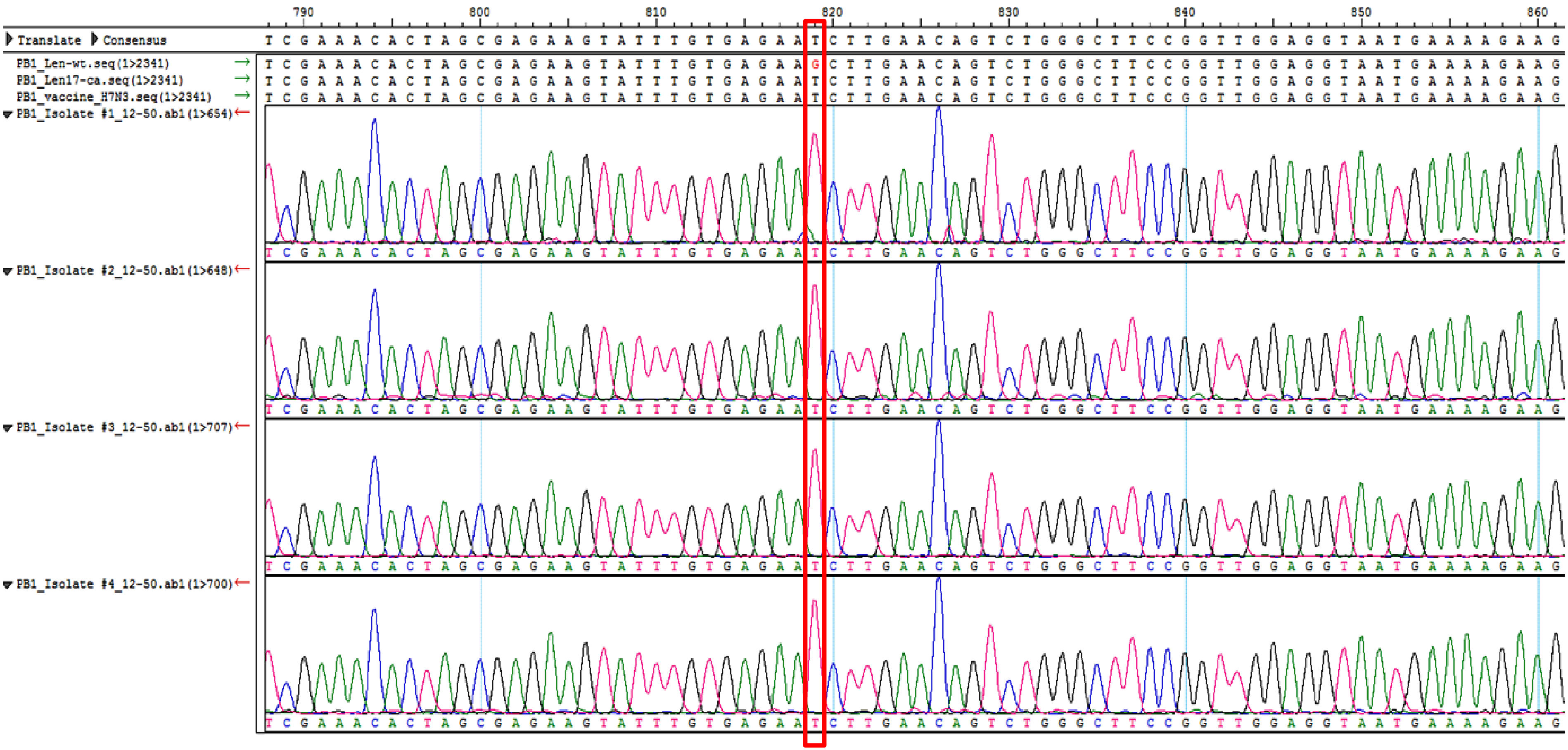

Len-wt: A/Leningrad/134/57 (H2N2) wild-type virus;
Len17-ca: A/Leningrad/134/17/57 (H2N2) master donor virus;
Vaccine_H7N3: A/17/mallard/Netherlands/00/95 (H7N3) LAIV

Mutation in PB1 gene: G-1795-A (Val-591-Ile)

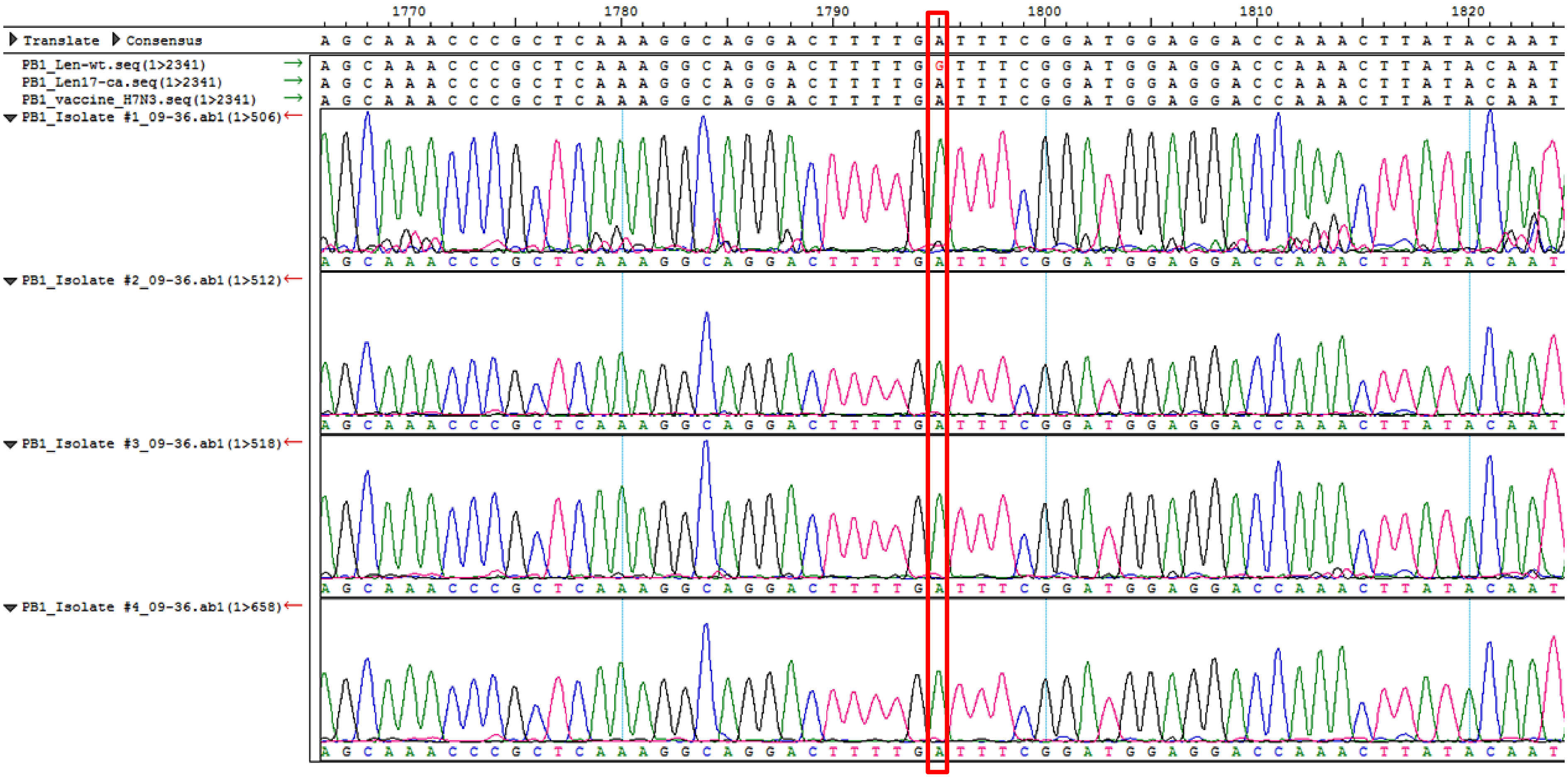

Len-wt: A/Leningrad/134/57 (H2N2) wild-type virus;
Len17-ca: A/Leningrad/134/17/57 (H2N2) master donor virus;
Vaccine_H7N3: A/17/mallard/Netherlands/00/95 (H7N3) LAIV

Mutation in PA gene: T-107-C (Leu-28-Pro)

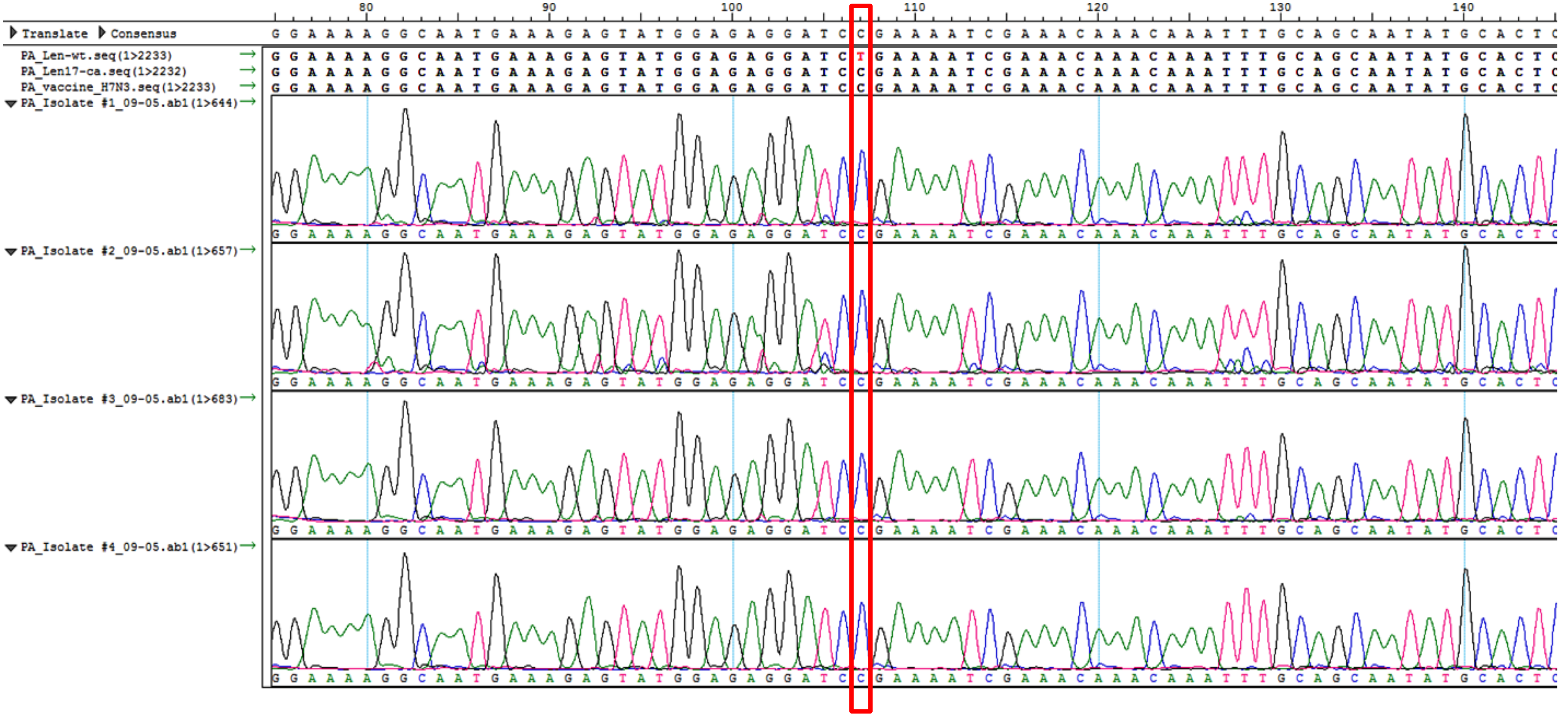

Len-wt: A/Leningrad/134/57 (H2N2) wild-type virus;
Len17-ca: A/Leningrad/134/17/57 (H2N2) master donor virus;
Vaccine_H7N3: A/17/mallard/Netherlands/00/95 (H7N3) LAIV

Mutation in PA gene: G-1045-T (Val-341-Leu)

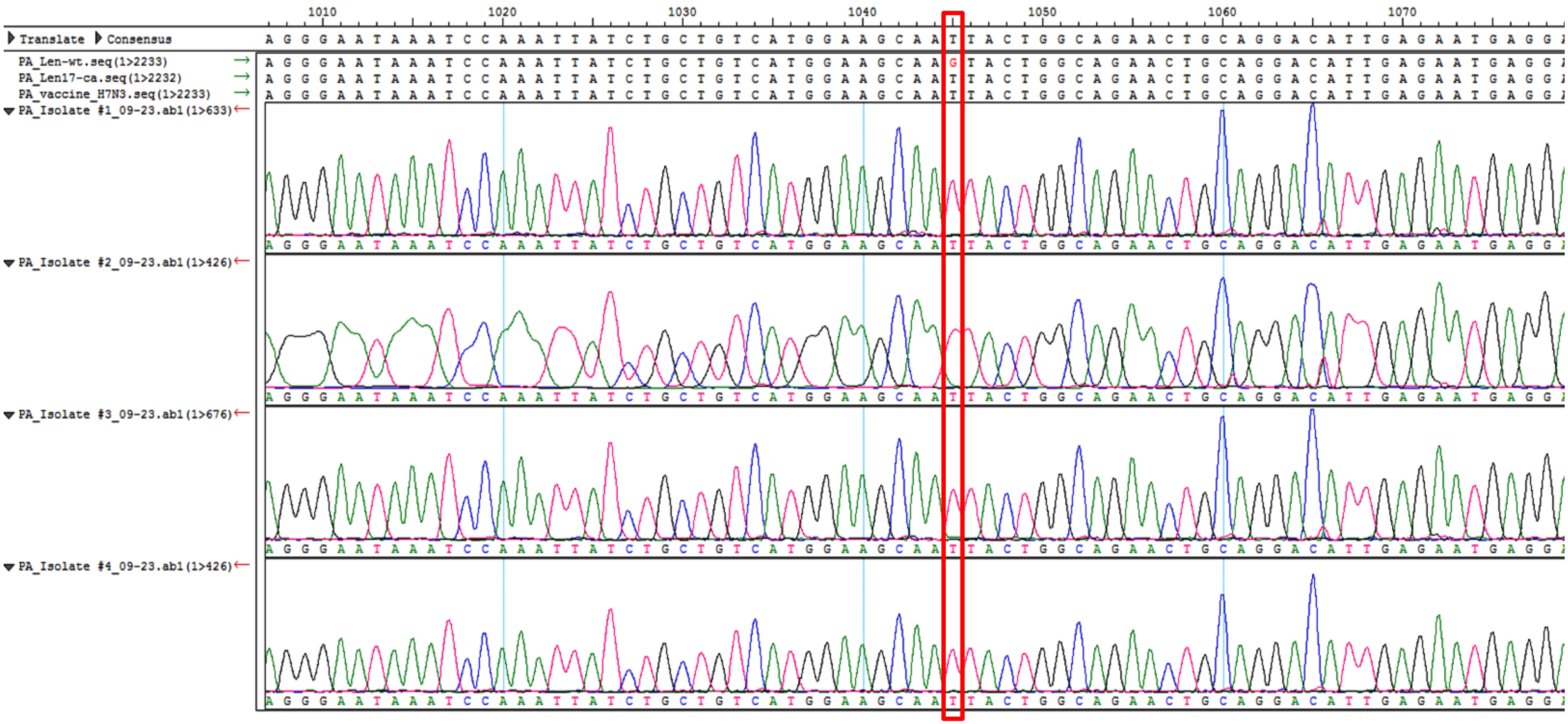

Len-wt: A/Leningrad/134/57 (H2N2) wild-type virus;
Len17-ca: A/Leningrad/134/17/57 (H2N2) master donor virus;
Vaccine_H7N3: A/17/mallard/Netherlands/00/95 (H7N3) LAIV

Mutation in NP gene: C-1066-A (Leu-341-Ile)

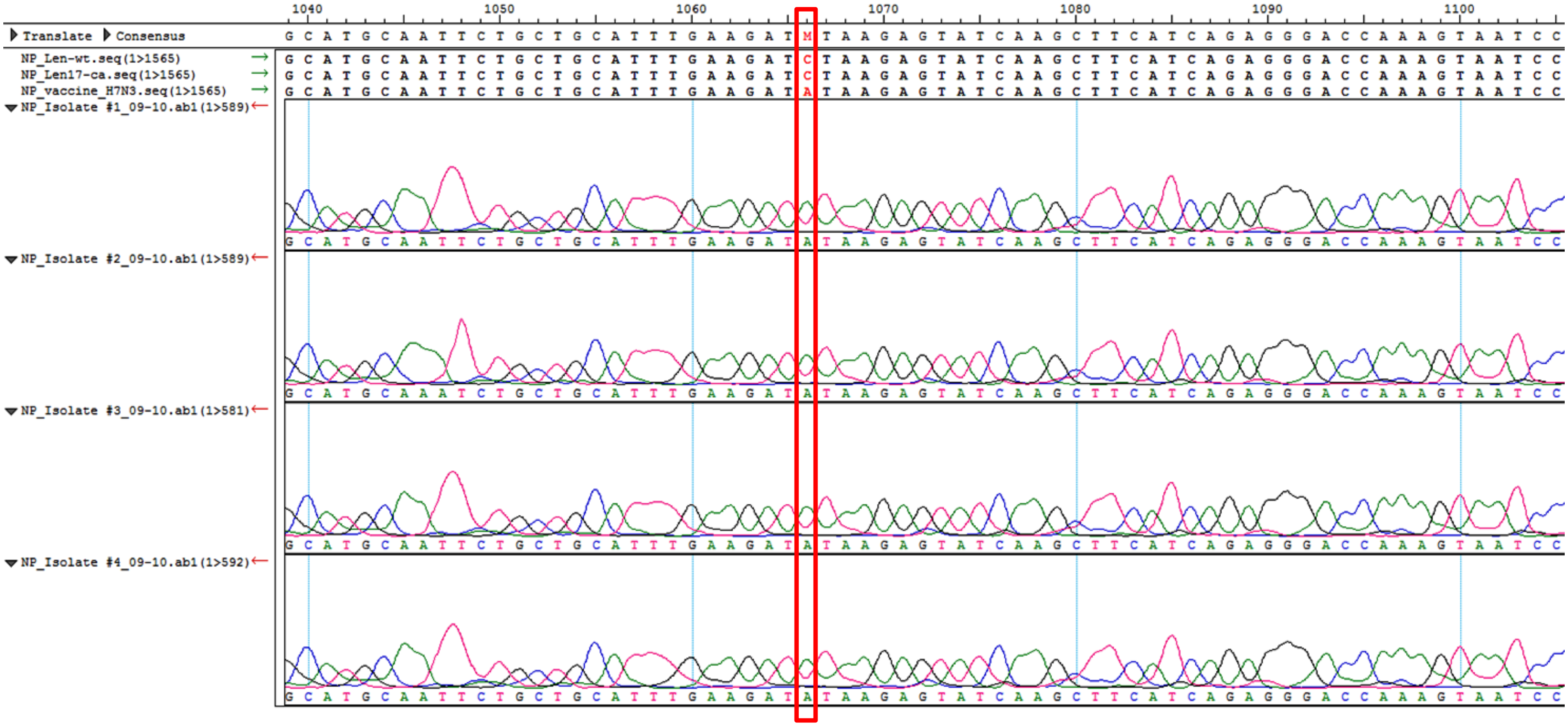

Len-wt: A/Leningrad/134/57 (H2N2) wild-type virus;
Len17-ca: A/Leningrad/134/17/57 (H2N2) master donor virus;
Vaccine_H7N3: A/17/mallard/Netherlands/00/95 (H7N3) LAIV

Mutation in M gene: A-68-G (M1: Ile-15-Val)

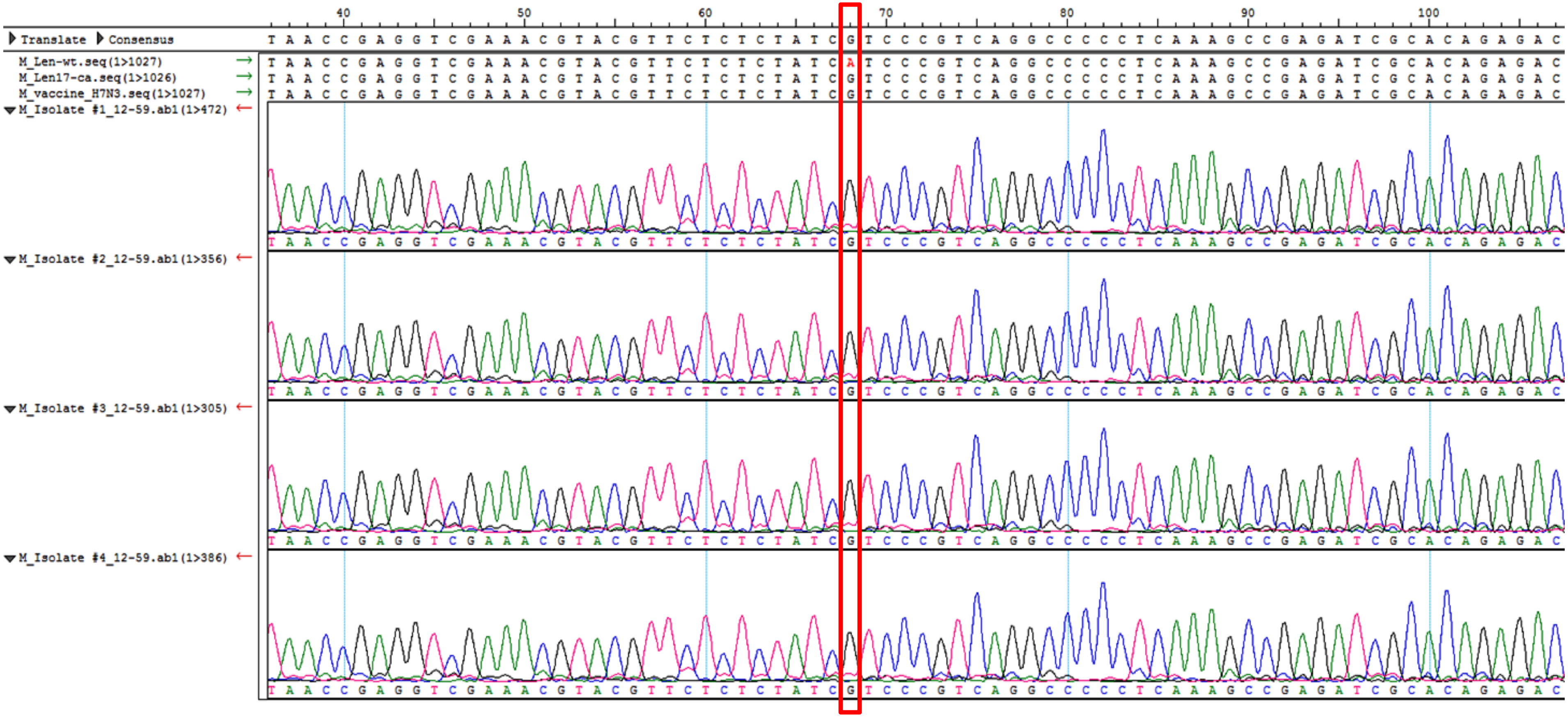

Len-wt: A/Leningrad/134/57 (H2N2) wild-type virus;
Len17-ca: A/Leningrad/134/17/57 (H2N2) master donor virus;
Vaccine_H7N3: A/17/mallard/Netherlands/00/95 (H7N3) LAIV

Mutation in M gene: T-457-G (M1: Phe-144-Leu)

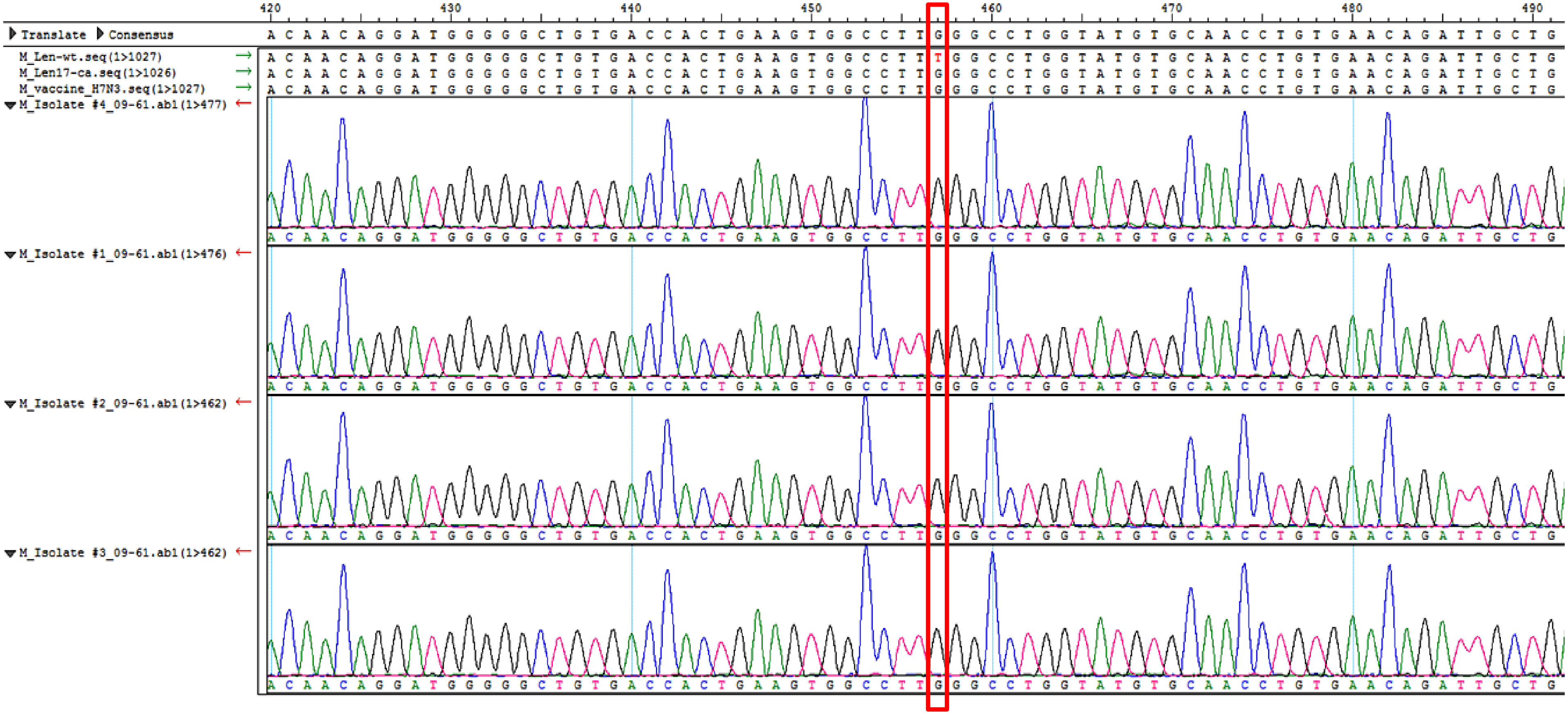

Len-wt: A/Leningrad/134/57 (H2N2) wild-type virus;
Len17-ca: A/Leningrad/134/17/57 (H2N2) master donor virus;
Vaccine_H7N3: A/17/mallard/Netherlands/00/95 (H7N3) LAIV

Mutation in NS gene: G-798-A (NS2: Met-100-Ile)

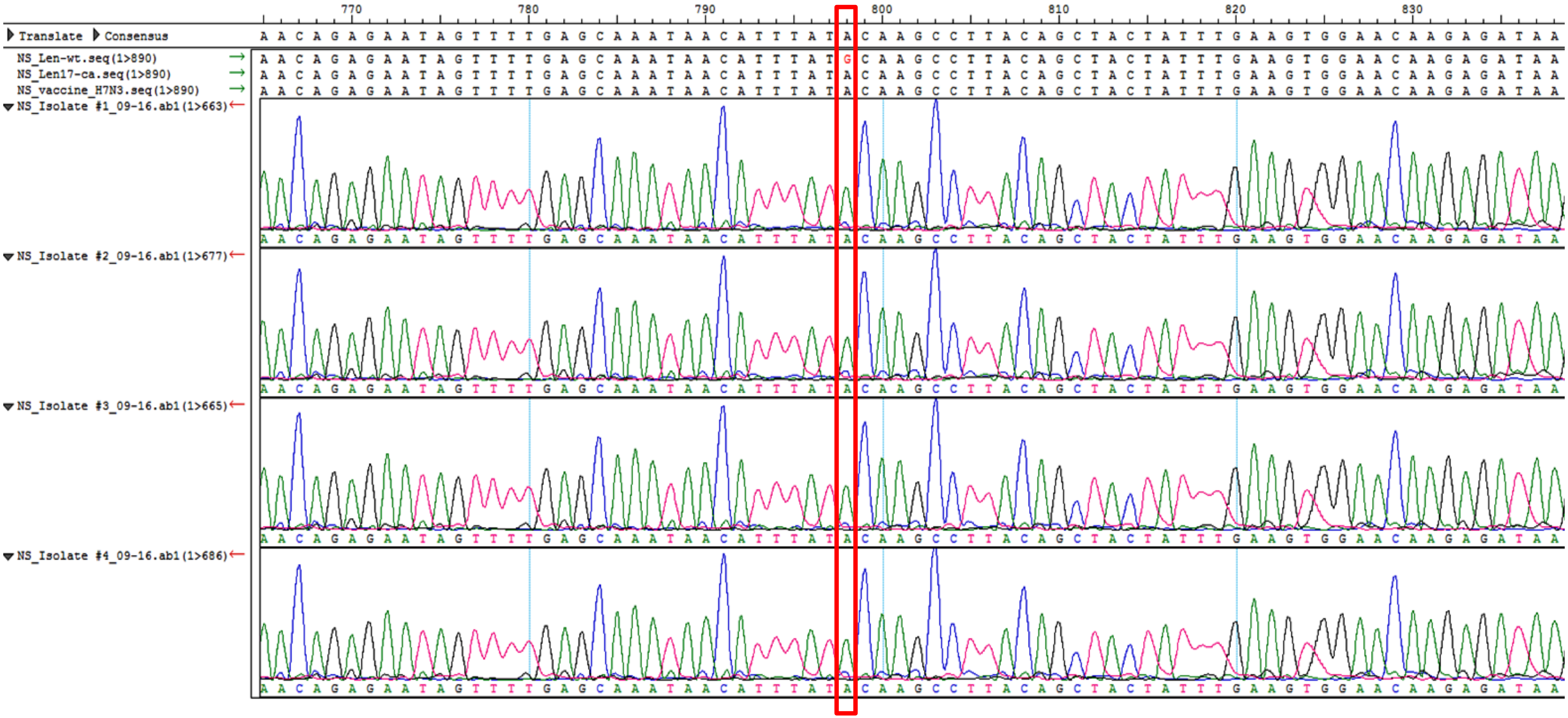

Len-wt: A/Leningrad/134/57 (H2N2) wild-type virus;
Len17-ca: A/Leningrad/134/17/57 (H2N2) master donor virus;
Vaccine_H7N3: A/17/mallard/Netherlands/00/95 (H7N3) LAIV
