# Supplementary material for: Assessment of Human Immune Responses to H7 Avian Influenza Virus of Pandemic Potential: Results from a Placebo–Controlled, Randomized Double–Blind Phase I Study of Live Attenuated H7N3 Influenza Vaccine
Source: PLoS One. 2014 Feb 12;9(2):e87962. doi: 10.1371/journal.pone.0087962 (PMC3922724; doi:10.1371/journal.pone.0087962)
Supplement: Table S1 — List of primers used for RT–PCR and sequencing analysis of H7N3 LAIV clinical isolates. (PDF) [file pone.0087962.s007.pdf]

List of primers used for RT–PCR and sequencing of H7N3 LAIV clinical isolates

#	Gene	Start position	Sequence	Note
1	PB2	F1	GATCGCTCTTCAGGGAGCGAAAGCAGGTC	Hoffmann*
2	PB2	R2341	ACTGGCTCTTCTATTAGTAGAAACAAGGTCGTTT	Hoffmann
3	PB1	F1	GATCGCTCTTCAGGGAGCGAAAGCAGGCA	Hoffmann
4	PB1	R2341	ACTGGCTCTTCTATTAGTAGAAACAAGGCATTTT	Hoffmann
5	PA	F1	GATCGCTCTTCAGGGAGCGAAAGCAGGTAC	Hoffmann
6	PA	R2233	ACTGGCTCTTCTATTAGTAGAAACAAGGTACTT	Hoffmann
7	HA	F1	GATCGCTCTTCAGGGAGCAAAAGCAGGGG	Hoffmann
8	HA	R1776	ACTGGCTCTTCTATTAGTAGAAACAAGGGTGTTTT	Hoffmann
9	NP	F1	GATCGCTCTTCAGGGAGCAAAAGCAGGGTA	Hoffmann
10	NP	R1565	ACTGGCTCTTCTATTAGTAGAAACAAGGGTATTTT	Hoffmann
11	NA	F1	GATCGCTCTTCAGGGAGCAAAAGCAGGTGC	Hoffmann
12	NA	R1466	ACTGGCTCTTCTATTAGTAGAAACAAGGAGTTTTTT	Hoffmann
13	M	F1	GATCGCTCTTCAGGGAGCAAAAGCAGGTAG	Hoffmann
14	M	R1027	ACTGGCTCTTCTATTAGTAGAAACAAGGTAGTTTTT	Hoffmann
15	NS	F1	GATCGCTCTTCAGGGAGCAAAAGCAGGGTG	Hoffmann
16	NS	R890	ACTGGCTCTTCTATTAGTAGAAACAAGGGTGTTTTT	Hoffmann
17	PB2	F1226	TGGCCATGGTATTTTCACA	Len/17**
18	PB2	R1745	TTGTATAGCATTGTAGGATTCTGA	Len/17
19	PB1	R1071	TGCCATTTTATTTGAGAACATTAT	Len/17
20	PB1	F1448	ATCAATATGAGCAAAAAGAAG	Len/17
21	PB1	F549	TAAAGAGGAGATGGAAATAACAAC	Len/17
22	PB1	F1586	CTGATATGAGCATTGGGGTAAC	Len/17
23	PB1	R1983	TTGGCTGGACCGTGAGCTGGC	Len/17
24	PA	R856	AGAGCAAGGAGGCCCATCCGG	Len/17
25	PA	R1834	CCTTGACAGAGGACTCGGCTT	Len/17
26	PA	F838	GATGGGCCTCCTTGCTCT	Len/17
27	PA	R372	CTATTCTCCTTGTAATCATAC	Len/17
28	PA	R1286	TTGAATCGGTCAGCTCGCATG	Len/17
29	NP	F745	GCACAAAGAGCAATGATGGAT	Len/17
30	M	R313	ACTGCTCTGTCCATGTTATTTG	Len/17
31	M	R616	CTCACTCGATCCAGCCATTT	Len/17
32	NS	F470	GGCTTTCACCGAAGAGGGAGC	Len/17

* Universal primers designed by E.Hoffmann et al. [7].

** Primers specific for A/Leningrad/134/17/57 (H2N2) MDV
